# A Fluorescent Cage for Supramolecular Sensing of 3‐Nitrotyrosine in Human Blood Serum

**DOI:** 10.1002/anie.202205403

**Published:** 2022-05-23

**Authors:** Lidia A. Pérez‐Márquez, Marcelle D. Perretti, Raúl García‐Rodríguez, Fernando Lahoz, Romen Carrillo

**Affiliations:** ^1^ Instituto de Productos Naturales y Agrobiología (IPNA-CSIC) Avda. Astrofísico Fco. Sánchez 3 38206 La Laguna Spain; ^2^ GIR MIOMeT-IU Cinquima-Química Inorgánica Facultad de Ciencias Campus Miguel Delibes Universidad de Valladolid 47011 Valladolid Spain; ^3^ Departamento de Física, IUdEA Universidad de La Laguna 38200 San Cristóbal de La Laguna Tenerife Spain

**Keywords:** Molecular Cages, Molecular Recognition, Nitrotyrosine, Sensors, Tetraphenylethene

## Abstract

3‐Nitrotyrosine (NT) is generated by the action of peroxynitrite and other reactive nitrogen species (RNS), and as a consequence it is accumulated in inflammation‐associated conditions. This is particularly relevant in kidney disease, where NT concentration in blood is considerably high. Therefore, NT is a crucial biomarker of renal damage, although it has been underestimated in clinical diagnosis due to the lack of an appropriate sensing method. Herein we report the first fluorescent supramolecular sensor for such a relevant compound: Fluorescence by rotational restriction of tetraphenylethenes (TPE) in a covalent cage is selectively quenched in human blood serum by 3‐nitrotyrosine (NT) that binds to the cage with high affinity, allowing a limit of detection within the reported physiological concentrations of NT in chronic kidney disease.

## Graphical abstract

Fluorescence by rotational restriction of two tetraphenylethenes (TPE) in a covalent cage is selectively quenched in human blood serum by 3‐nitrotyrosine, a key biomarker of kidney injury. This cage, with a limit of detection under physiologically relevant concentrations, is the first reported fluorescent supramolecular sensor for such a relevant compound.

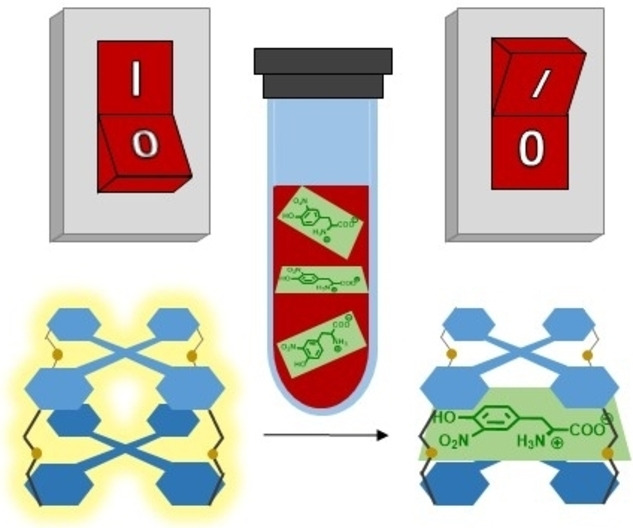

## Introduction

**Figure 1:**
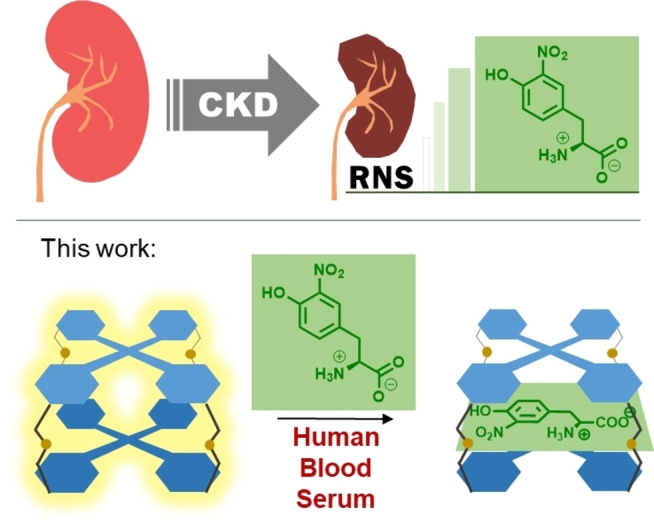
Chronic Kidney Disease (CKD) has been reported to increase 3‐nitrotyrosine (NT) in blood due to nitration of tyrosine by Reactive Nitrogen Species (RNS). Herein we have developed the first reported supramolecular sensor of NT: A tetraphenylethene molecular cage turn‐off sensor that works in human blood serum.

Chronic kidney disease (CKD) is considered as a leading public health problem worldwide and its high prevalence is expected to increase even more due to unhealthy habits, obesity and aging of the world's population.^[1]^ Unfortunately, CKD is silent during the initial stages and therefore a reliable and early detection of renal damage is an essential strategy to reduce the disease burden. The standard procedure for the clinical assessment of kidney function relies on changes in serum creatinine. Several approaches have been recently reported for the quantification of creatinine. Particularly elegant are the supramolecular sensing displays designed by Ballester,^[2]^ Pischel and co‐workers,^[3]^ which show an extraordinary simplicity and sensibility. However, in many cases serum creatinine levels are not well correlated to a lack of renal function,[^4^, ^5^] and therefore diagnosis and treatment of kidney disease could benefit from the evaluation of alternative biomarkers. In this regard, it has been reported that chronic renal failure patients show distinctively high levels of 3‐nitrotyrosine (NT) in blood.^[6]^ Even when tyrosine nitration is closely related to several human pathologies[^7^, ^8^, ^9^, ^10^, ^11^, ^12^, ^13^] and aging,^[14]^ high blood concentrations of NT have only been reported for renal damage (Figure 1, top). One of the reasons for such distinctive high levels of NT is probably related to the decreased ability of damaged kidneys to excrete it in the urine. But the most important factor might be the constitutive expression of inducible NO synthase (iNOS) in the kidney,^[15]^ which enables the rapid upregulation of its expression upon systemic inflammations in such an organ,^[16]^ and as a consequence, the generated peroxynitrite converts tyrosine residues into 3‐nitrotyrosine (NT). Therefore, NT accumulation is an excellent biomarker for kidney disease. Unfortunately, current methods for NT detection and quantification lack simplicity and affordability and therefore they prevent any fast and routine analysis of such a biomarker.[^17^, ^18^, ^19^, ^20^, ^21^]

An optimal approach to low‐cost and highly sensitive sensing methods is the development of fluorescent sensors,[^22^, ^23^, ^24^, ^25^] and in fact, excellent examples of fluorescent supramolecular sensing of relevant biomarkers in water or biofluids have proven their importance.[^3^, ^26^, ^27^, ^28^, ^29^, ^30^, ^31^, ^32^, ^33^] In this regard, a turn‐off fluorescent sensor for NT seems quite reasonable for two reasons: Firstly, nitroaromatics are efficient quenchers of fluorescence,[^34^, ^35^, ^36^, ^37^] as has been already proven in the sensing of explosives such as TNT or picric acid;[^38^, ^39^] secondly, nitroaromatics are a very rare class of biological compounds or natural products,^[40]^ and therefore no interference or false positives are expected.^[41]^

We envisioned a high sensitivity tetraphenylethene (TPE) molecular cage turn‐off sensor (Figure 1, bottom).[^42^, ^43^] Indeed, TPE is the most paradigmatic example of the well‐known aggregation‐induced emission (AIE) phenomenon.[^44^, ^45^] The fluorescence of this kind of compounds is extinguished when phenyls rings have freedom of rotation, whereas restriction of such rotation remarkably increases fluorescence. Therefore, confined TPE within the cage walls, will lead to fluorescence, which will be quenched by supramolecular encapsulation of nitro‐aromatic compounds such as NT, probably due to an electron transfer towards the electron‐poor aromatic compound.^[46]^ Additionally, supramolecular binding should be reinforced in water, particularly for NT, which is known to display a considerable hydrophobic character.^[47]^

Herein we have synthesized a TPE‐based fluorescent molecular cage that binds NT with high affinity in aqueous media. Such binding concomitantly quenches the fluorescence of the cage with no interferences and high selectivity, and therefore, it can be used as a chemosensor for NT even in human blood serum.

## Results and Discussion

**Scheme 1:**
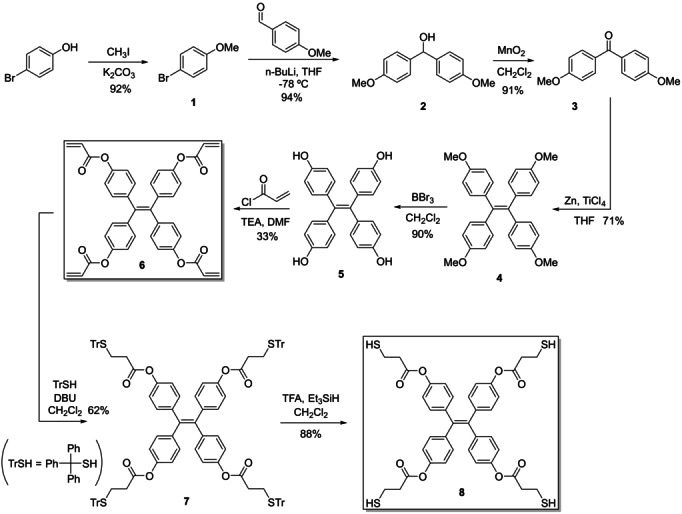
Synthesis of cage precursors.

The fluorescent cage was synthesized by a sequential thiol‐Michael addition methodology developed by our group.^[48]^ First, TPE building block **6** (Scheme 1) was synthetized by a McMurry reaction from benzophenone derivative **3**.^[49]^ Then, after a fourfold thiol‐Michael addition with triphenylmethanethiol (TrSH) followed by removal of the trityl group, **8** was obtained.

**Scheme 2:**
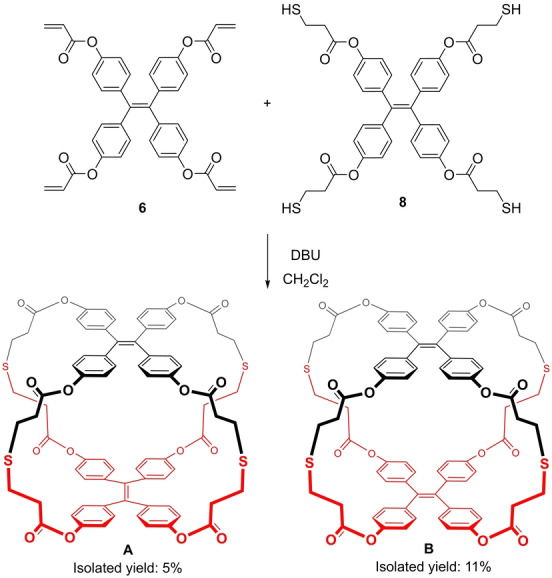
Synthesis of the fluorescent cage. Two orientational isomers are obtained.

The final fourfold thiol‐Michael addition between precursors **6** and **8** gave two main products (Scheme 2), which were named cage **A** (less polar) and cage **B** (more polar). They were isolated by chromatography in silica gel with a mixture of ethyl acetate–toluene (4 : 6) in 5 % and 11 % yield respectively. Both compounds showed very similar ^1^H‐NMR and ^13^C‐NMR spectra and the same mass spectrum (ESI), which led us to hypothesize that they were two orientational isomers: considering the relative orientation of the ethenes in each TPE, the parallel isomer and the orthogonal one (Scheme 2). Actually, there are precedents reporting different orientational isomers for TPE cages.[^32^, ^42b^]

**Figure 2:**
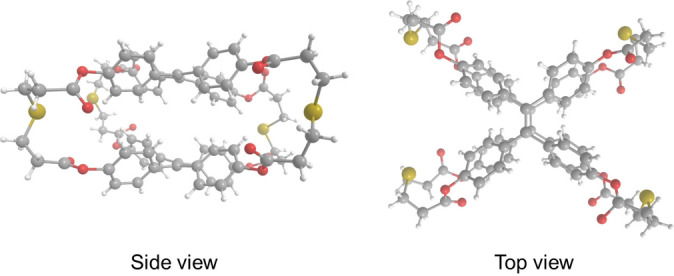
X‐ray structure of cage **B**.

In order to assign the correct isomeric structure to each cage, we tried to grow crystals for both of them. We only obtained crystals suitable for X‐ray studies for cage **B**, and its structure was measured and solved (Figure 2). Although the single‐crystal X‐ray data were of poor quality, it was sufficient to unambiguously determine the connectivity of the structure, showing that it displays a parallel geometry. As a consequence, it indirectly confirms that cage **A** is the orthogonal isomer.^[50]^

Detection of different stereoisomers for each orientational isomer was not possible, probably due to a rapid racemization in solution at room temperature, indicating that TPE rotational enantiomers could interconvert and therefore phenyl rotation is not fully inhibited.^[51]^

**Figure 3:**
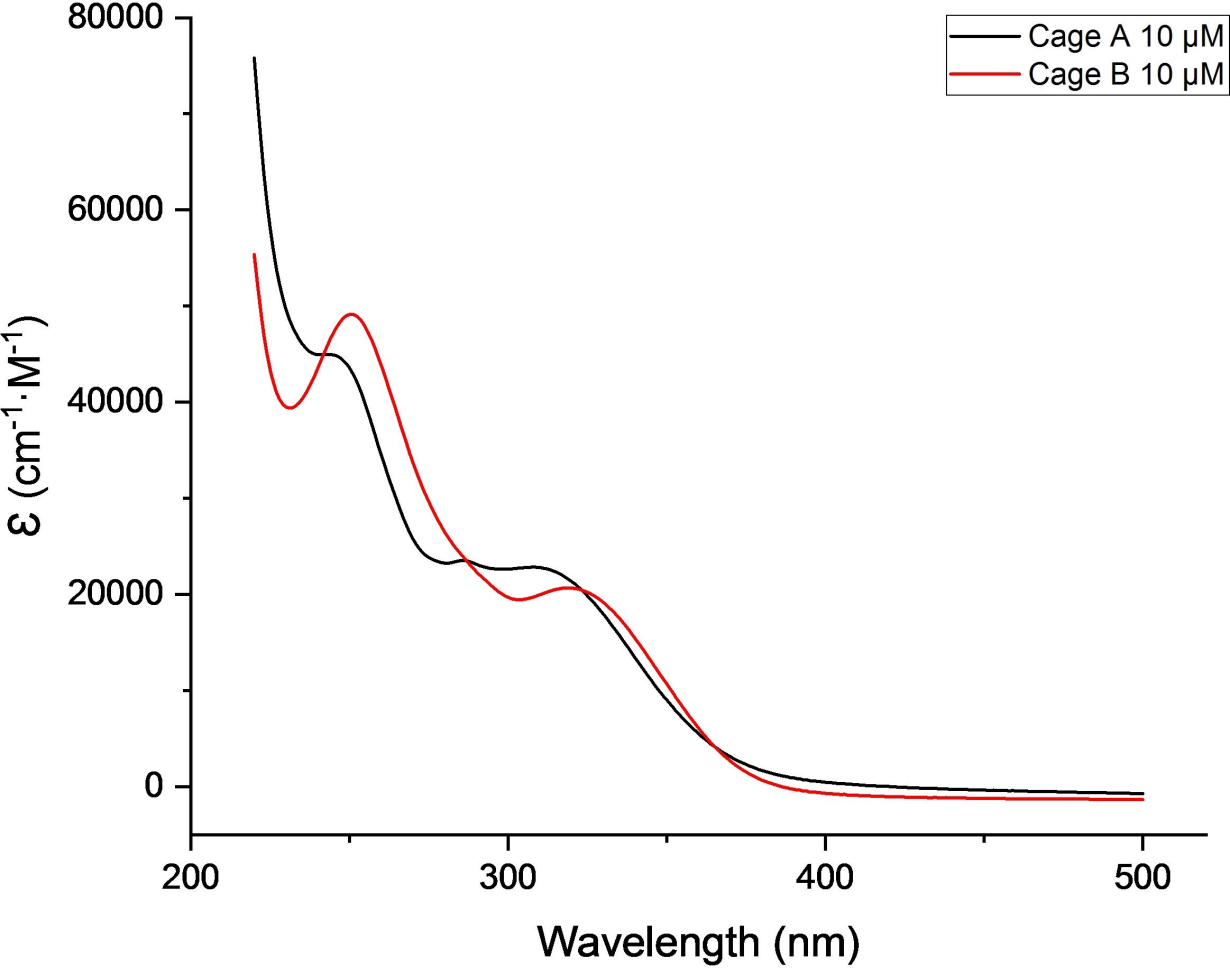
UV spectra of cages **A** and **B** (10 μM) in 2 : 8 THF/H_2_O.

UV/Vis absorption spectra were obtained for both cages. Figure 3 shows the molar extinction coefficient for cages **A** and **B** (10 μM) in 2 : 8 THF/H_2_O. Two main bands, centered at about 250 and 320 nm, are observed in cage **B**, which are more intense and better resolved than those of cage **A**. Moreover, the absorption bands of cage **B** are slightly red shifted as compared to those of cage **A**.

**Figure 4:**
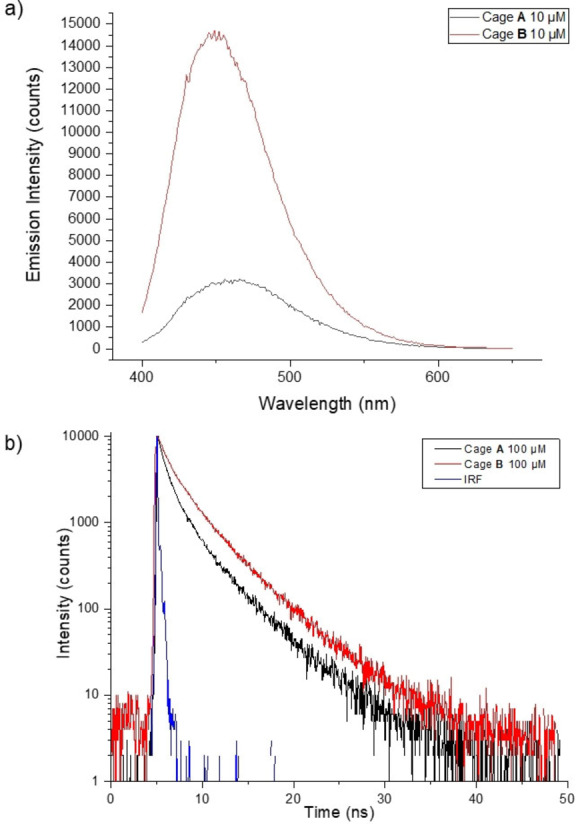
a) Fluorescence spectra of cage **A** (10 μM) and cage **B** (10 μM) in 2 : 8 THF/H_2_O. b) Fluorescence lifetime profile (excited at 375 nm) of cage **A** (100 μM) and **B** (100 μM) in 2 : 8 THF/H_2_O. IRF: instrument response function.

As expected, cages **A** and **B** emit an intense blue fluorescence when excited in the UV absorption bands, due to rotational restriction of both tetraphenylethene (TPE) units once they are incorporated into the structure of the cage. The fluorescence emission spectra of cages **A** and **B** under UV excitation are shown in Figure 4a. The excitation wavelength was chosen at the high wavelength tail of the absorption band (*λ*_exc_=375 nm) to avoid intrinsic absorption of proteins when measuring biofluids. Both spectra display a maximum roughly at 460 nm and a broad full width at half maximum of about 75 nm. Notably, the emission intensity of cage **B** was higher than that of cage **A**. Fluorescence quantum yields (Φ_fluo_) of both cages were calculated using the single point method, which basically consists on the comparison of the integrated emission intensity of the sample to that of a known standard.[^49^, ^52^] The obtained values were Φ_fluo_=0.2±0.03 and 0.4±0.06, for cage **A** and **B**, respectively.

Time‐resolved fluorescence was also measured, at the maximum of their emission bands, under UV pulsed laser excitation at 375 nm (Figure 4b). A slower decay was obtained for cage **B**. In both cases, the fluorescence decay curves were not monoexponential. However, the experimental decay curves could be fitted to a bi‐exponential function and the average lifetimes were calculated, providing a value of *τ*_fluo_=2.0±0.2 ns for cage **A**, while a large average lifetime around *τ*_fluo_=3.1±0.3 ns was obtained for cage **B**, which is in the same range as many commercially available dyes in aqueous solvents.^[53]^ The shorter average lifetime detected for cage **A** may be related to non‐radiative relaxation processes, which could explain its smaller quantum yield.

Once the cages had been properly characterized, we examined the supramolecular properties of these receptors. Obviously, due to the much better fluorescence parameters displayed by cage **B**, this cage was chosen for all the supramolecular and sensing studies.

**Figure 5:**
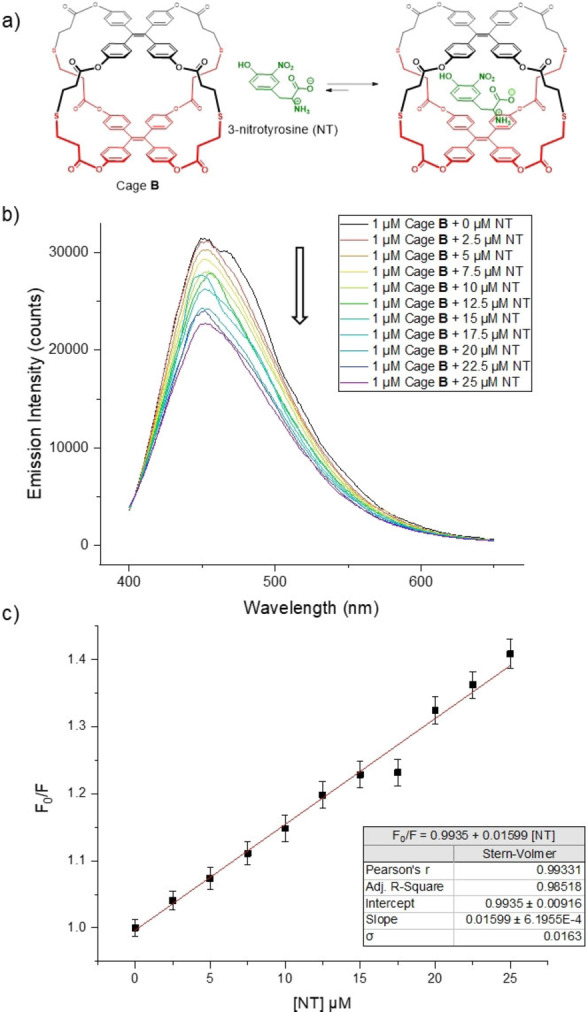
a) Supramolecular binding of NT inside cage **B**. b) Fluorescence quenching of cage **B** (1 μM) at different NT concentrations in 2 : 8 of THF/H_2_O. c) Stern–Volmer plot of cage **B** fluorescence quenching with NT in 2 : 8 of THF/H_2_O. Error bars correspond to the standard deviation of three measurements.

It is worth mentioning that our aim to detect NT in serum by supramolecular sensing is far from easy. Indeed, host–guest binding in water or biological fluids remains a major challenge.^[54]^ In our case, we hypothesized that the stacked geometry of TPE walls and the appropriate size of the cavity would allow the insertion of NT (Figure 5a). Such encapsulation could be favored by a clear complementary electronic nature between host and guest, a plausible cation–π interaction with the ammonium from the NT, and obviously, by the hydrophobic effect, which is very likely to play a central role in a such binding event,[^55^, ^56^] particularly considering the increase in hydrophobicity of tyrosine after nitration.^[47]^ Fluorescence titrations between cage **B** and NT were performed, in order to explore the supramolecular association as well as the fluorescence quenching upon interaction between NT and the cage.^[57]^

As already mentioned, quenching of the fluorescence is expected as the concentration of the quencher increases.[^34^, ^35^, ^36^, ^37^] Figure 5b shows the fluorescence emission spectra of cage **B** (1 μM) as a function of the concentration of NT. There is a clear quenching of the fluorescence intensity as the concentration of the biomarker increases.

In order to unravel the nature of the supramolecular binding between NT and cage **B**, fluorescence quenching can be analyzed using the Stern–Volmer equation 1:

(1) $$F{}_{0}/F=1-K{}_{\mathrm{SV}}\left[Q\right]$$

where *F*_0_ and *F* are the fluorescence intensities before and after the addition of the quencher, respectively; [Q] is the concentration of the quencher (in our case NT); and *K*_SV_ is the Stern–Volmer quenching constant, which indicates the sensitivity of the cage to the quencher.^[52]^ A linear dependence of the ratio *F*_0_/*F* on the quencher concentration can be observed in Figure 5c, which means there is only one fluorophore in the solution, namely cage **B**. The slope of the linear regression between *F*_0_/*F* and [NT] gave a value of *K*_SV_=(1.60±0.06)⋅10^4^ M^−1^.

**Figure 6:**
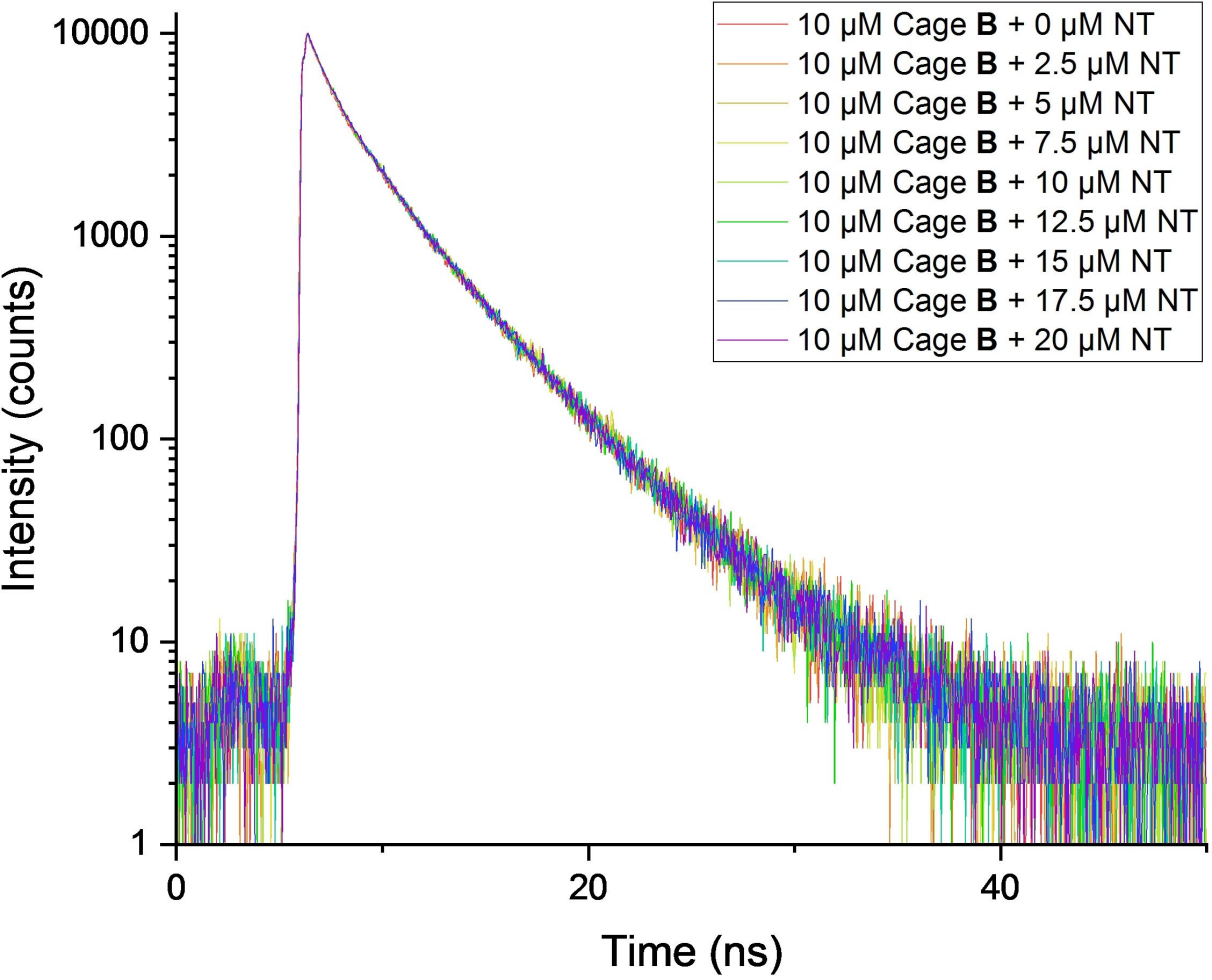
Fluorescence lifetime profile (excited at 375 nm) of cage **B** (10 μM) in 2 : 8 of THF/H_2_O and different NT concentrations.

We can assume that *K*_SV_ is equivalent to the association constant for the supramolecular binding (*K*_a_) only if two conditions are met: 1) The supramolecular complex formed is fluorescently silent; 2) quenching is static in nature.[^58^, ^59^] First of all, we have just mentioned that the linear Stern–Volmer plot obtained implies only one emissive specie (cage **B**) and therefore, we can assume that the supramolecular complex is fluorescently silent. On the other hand, to confirm the static quenching mechanism, an evaluation of the fluorescence lifetime at different concentrations of NT is required.^[48]^ Indeed, if static quenching is occurring, a non‐fluorescent supramolecular complex is formed, and therefore, the fluorescence detected would only come from free cage **B** molecules. Consequently, the fluorescence lifetime would remain the same, irrespective of the concentration of NT. However, in the case of dynamic quenching a shortening of the lifetime would be expected as the NT concentration increases. As clearly seen in Figure 6, similar decay curves are obtained regardless of the quencher concentration. This result indicates that static quenching is responsible for the fluorescence quenching observed.^[60]^ Therefore, after all these photophysical experiments, we can conclude that there is a proper encapsulation of NT inside cage **B** in THF/water (2 : 8), with an association constant of *K*_a_=(1.60±0.06)×10^4^ M^−1^. Actually, UV titration in the same solvent mixture led to a very similar association constant.^[49]^

Another important parameter to characterize the sensor is the limit of detection (LoD), which refers to the smallest concentration of an analyte from which it is possible to deduce its presence in the test sample.^[61]^ It is given by:

(2) LoD=3.3σ/b

where *σ* represents the standard error of the linear regression between *F*_0_/*F* and [NT] (Figure 5c), and *b* is its slope, or in other words, the *K*_SV_ constant.^[62]^ Considering that the standard deviation of the linear regression is *σ*=0.0163, then the limit of detection of cage **B** is LoD=3 μM. Reported serum concentrations of NT are around 28 μM in renal failure patients without septic shock, and 118 μM in patients with septic shock.^[6]^ Therefore this sensor could potentially detect NT concentrations which are relevant for the clinical practice.

**Figure 7:**
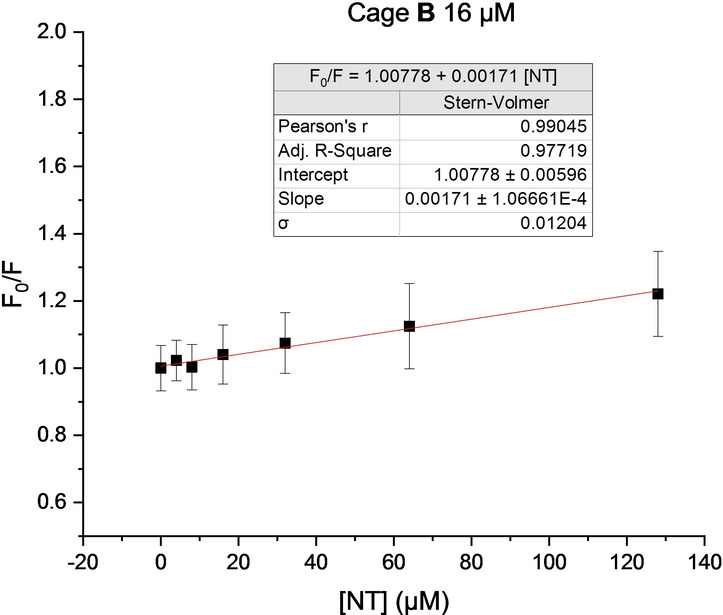
Regression analysis of 16 μM of cage **B** in the presence of increasing amounts of NT in human serum. Concentrations of NT are those of the final mixture analyzed. Error bars correspond to the standard deviation of eighteen measurements.

Encouraged by the promising results obtained in water, we applied this fluorescent cage to the determination of NT in human blood serum. Three different commercially available human sera from clotted whole blood collected from volunteer donors were employed.^[49]^ They are produced by allowing the whole blood to clot naturally, and the serum is extracted from the resultant fractionation. One part of the serum (20 μL) was diluted with three parts of PBS buffer (pH 7.4) containing various amounts of NT, and finally one part of a solution of cage **B** in DMSO at different concentrations was added. Fluorescence quenching was evaluated in 96‐well black polystyrene plates and analyzed using a microplate reader with an excitation filter wavelength of 380 nm, and an emission filter wavelength of 470 nm. Different concentrations of cage **B** were evaluated to optimize the amount of sensor required to detect increasing concentrations of NT in the serum, and all the data obtained was statistically analyzed.^[49]^ Gratifyingly, a good linear correlation was observed in all the experiments, although the best data were obtained for a cage concentration of 16 μM (Figure 7). According to the slope, a decrease in the association constant, *K*_a_=(1.71±0.11)×10^3^ M^−1^, is also observed compared to that in the initial experiments in THF/water, which is most likely due to the use of DMSO as organic solvent in the serum experiments for practical and technical reasons. Importantly, no relevant interferences were observed even in a complex matrix such as human blood serum, probably due to the scarcity of other nitro‐aromatic compounds or potential quenchers.^[63]^ Indeed, we tested several other bioanalytes such as tyrosine, creatinine, urea, tryptophan, or glucose, and none of them induced quenching of the cage fluorescence.^[49]^ Finally the limit of detection in the diluted mixture could be calculated by Equation (2),^[49]^ yielding LoD=23 μM.^[64]^ Further studies aimed to increase the affinity of the receptor for NT will help to enhance the detection limit and the applicability of this kind of sensors.

## Conclusion

The first supramolecular sensor of 3‐nitrotyrosine (NT) has been developed. A fluorescent cage, easily synthesized by two sequential thiol‐Michael click reactions, is able to encapsulate NT with high affinity in aqueous media. Such supramolecular interaction concomitantly induces a fluorescence quenching, which is linearly correlated with the concentration of NT in human blood serum, with a limit of detection within the reported values found in patients with chronic kidney disease. Therefore, this kind of chemosensor might help clinical assessment of renal injuries, in combination with other routinely examined bio‐markers. Obviously, further studies designed to improve the affinity of the sensor for NT in human serum will enhance the analytical detection of this relevant biomarker. We really hope this work also encourages other groups to find different and better approaches for the specific and efficient supramolecular sensing of nitrotyrosine either by reevaluating all the plethora of chemosensors for nitroaromatic explosives,[^38^, ^39^] or by improving and modulating the properties of TPE cages by means of dynamic covalent chemistry.[^42f^, ^42g^, ^42h^, ^42i^] All those combined research efforts will definitely have a profound impact on the diagnosis and treatment of renal diseases.

## Conflict of interest

The authors declare no conflict of interest.

1

## Supporting information

As a service to our authors and readers, this journal provides supporting information supplied by the authors. Such materials are peer reviewed and may be re‐organized for online delivery, but are not copy‐edited or typeset. Technical support issues arising from supporting information (other than missing files) should be addressed to the authors.

Supporting Information

## Data Availability

The data that support the findings of this study are available in the Supporting Information of this article.
