# Supplementary material for: A Fluorescent Cage for Supramolecular Sensing of 3‐Nitrotyrosine in Human Blood Serum
Source: Angew Chem Int Ed Engl. 2022 May 23;61(28):e202205403. doi: 10.1002/anie.202205403 (PMC9401051; doi:10.1002/anie.202205403)
Supplement: Supplementary file 1 — Supporting Information [file ANIE-61-0-s001.pdf]

Angewandte Chemie
Eine Zeitschrift der Gesellschaft Deutscher Chemiker

Supporting Information

A Fluorescent Cage for Supramolecular Sensing of 3-Nitrotyrosine in Human Blood Serum

L. A. Pérez-Márquez, M. D. Perretti, R. García-Rodríguez, F. Lahoz, R. Carrillo**

SUPPLEMENTARY INFORMATION

TABLE OF CONTENTS

1. General experimental details	S2
2. Synthesis and characterization of precursors	S2
3. Synthesis and characterization of cages	S5
4. X-ray structure determination	S6
5. Photophysical properties of cages<br>5.1. Absorption<br>5.2. Emission<br>5.3. Quantum yield<br>5.4. Lifetime	S12
6. Binding of 3-nitrotyrosine (NT) to cage **B** in THF/Water (2:8)<br>6.1 Limit of detection based on the standard deviation of the blank<br>6.2 Confirmation of the static quenching mechanism<br>6.3 UV-titration	S18
7. Binding of 3-nitrotyrosine (NT) to cage **B** determined by fluorescence quenching in human blood serum<br>7.1 Determination of limit of detection in human serum	S21
8. Binding of cage **B** with 1,3,5-trinitrotoluene (TNT) in ethyl acetate.	S41
9. Selectivity measurements	S42
10. NMR spectra	S43
11. References	S55

1. General experimental details.

^{1}H NMR spectra were recorded at 600 and 500 MHz, and ^{13}C NMR spectra were recorded at 150 MHz. Chemical shifts were reported in units (ppm) by assigning TMS resonance in the ^{1}H NMR spectrum as 0.00 ppm (chloroform, 7.26 ppm; dimethyl sulfoxide-d^6 2.50 ppm). Data were reported as follows: chemical shift, multiplicity (s = singlet, d = doublet, dd = double doublet, t = triplet, ddd = double double doublet, m = multiplet, and br = broad), coupling constant (J values) in Hz, and integration. Chemical shifts for ^{13}C NMR spectra were recorded in ppm from tetramethylsilane using the central peak of $CDCl_3$ (77.16 ppm), dimethyl sulfoxide (39.52 ppm) as the internal standard. Accurate mass values (HRMS) were determined by electronic impact (EI-TOF). UV-Vis spectroscopy was performed on a Double beam spectrophotometer (Cary Series UV-VIS-NIR Spectrophotometer from Agilent Technologies). Measurements of anisotropy, lifetime and emission spectra have been made with the Edinburgh Instruments LifeSpec II spectrofluorometer with a 375 nm laser. The measurement and control software connected to LifeSpec II is called F900. The spectra corresponding to the quenching experiments have been measured with an Edinburgh Instruments FLS1000 series spectrometer, also controlled with F900. As an excitation source, a continuous spectrum Xe lamp has been used, whose wavelength has been chosen with an input monochromator. The graphical analysis of the data obtained was carried out with Origin 7.0 from OriginLab. Plate reader fluorescence intensities were measured by FLUOstar Omega (BGM Labtech). The lecture of multiwell were made using Omega Control software and data processing was done using MARS 3.31 software. Heating of reactions was always performed by Heidolph Heat-on packs. Flash column chromatography was performed using silica gel, 60 Å and 0.2−0.5 mm with the indicated solvent system according to standard techniques. Compounds were visualized on TLC plates by use of UV light. All the solvents were treated according to reported methods.

2. Synthesis and Characterization of Precursors.

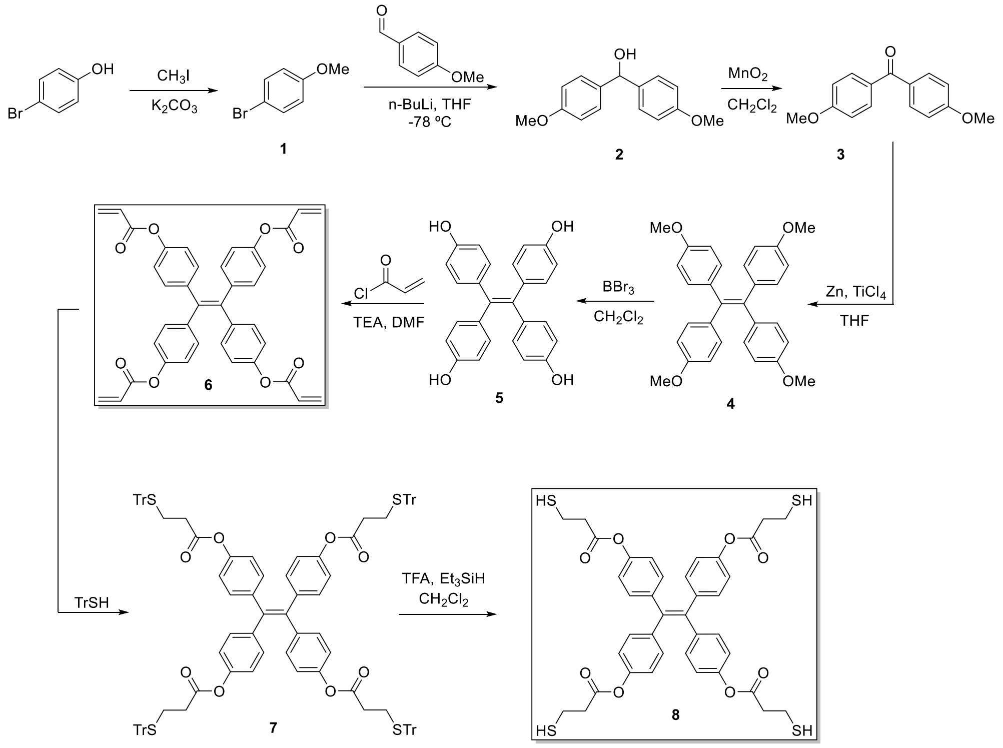

Figure S1. Synthetic route of fluorescent cage precursors

1-bromo-4-methoxybenzene (1). Based on a literature procedure,[1] 4-bromophenol (2.00 g, 11.56 mmol) was solved in acetone (95 mL). K_2CO_3 (8.00 g, 57.80 mmol) was added and stirred for 10 min at room temperature under inert atmosphere. Iodomethane (0.94 mL, 15.03 mmol) was added into the flask and the mixture was kept refluxing overnight. Then, the mixture was filtered and concentrated in vacuo. The final compound was purified by flash chromatography (SiO_2, ethyl acetate/hexane, 10:90) to provide product **1** (1.65 g, 92%) as a colourless oil. **^{1}H NMR** (500 MHz, $CDCl_3$, 298 K): δ ppm 7.37 (d, *J* = 8.9 Hz, 2H), 6.78 (d, *J* = 8.9 Hz, 2H), 3.78 (s, 3H). **^{13}C NMR** (150 MHz, $CDCl_3$, 298 K): δ ppm 158.84, 132.37, 115.87, 112.96, 55.57. **HR-MS** (EI) m/z: calcd for $C_7H_7O^{81}Br$ [M^+]: 187.9660, found: 187.9659; calcd for $C_7H_7O^{79}Br$ [M^+]: 185.9680, found: 185.9682.

Bis(4-methoxyphenyl)methanol (2). Based on a literature procedure,[2] compound **1** (0.7871 g, 4.2 mmol) was solved in THF (30 mL) at -78 ^oC under a nitrogen atmosphere. n-BuLi (1.62 mL, 4.21 mmol) was added dropwise, and the mixture was stirred for 30 min at the same temperature. 4-methoxybenzaldehyde (0.43 mL, 3.51 mmol) in THF (10 mL) was added dropwise to reaction mixture. The progress of the reaction was monitored by TLC. When TLC showed no starting material (4 h later approximately), MeOH (35 mL) was added to the solution at -78°C which was allowed to stir at room temperature for 15 min. After evaporating the solvent in vacuo, reaction mixture was dissolved in CH_2Cl_2 and washed with saturated $NaHCO_3$ solution. Combined organic phases were dried over anhydrous $MgSO_4$, filtered and concentrated in vacuo. Obtained crude mixture was purified by flash chromatography (SiO_2, ethyl acetate/hexane, 20:80) to provide product **2** (0.76 g, 94%). **^{1}H NMR** (500 MHz, $CDCl_3$, 298 K): δ ppm 7.28 (d, *J* = 8.7 Hz, 4H), 6.86 (d, *J* = 8.7 Hz, 4H), 5.78 (s, 1H), 3.79 (s, 6H), 2.08 (s, 1H). **^{13}C NMR** (150 MHz, $CDCl_3$, 298 K): δ ppm 158.97, 134.95, 128.58, 113.85, 79.02, 55.39. **HR-MS** (EI) m/z: calcd for $C_{15}H_{16}O_3$ [M^+]: 244.1099, found: 244.1098.

Bis(4-methoxyphenyl)methanone (3). Based on a literature procedure,[2] MnO_2 (0.54 g, 6.20 mmol) was added to a solution of **2** (0.7572 g, 3.10 mmol) solved in CH_2Cl_2 (30 mL) under a nitrogen atmosphere at room temperature. Reaction was allowed to stir overnight. The progress of the reaction was monitored by TLC and when it showed no starting material, reaction mixture was filtered over celite. Then the crude was concentrated in vacuo to provide product **3** (0.68 g, 91%). **^{1}H NMR** (500 MHz, $CDCl_3$, 298 K): δ ppm 7.79 (d, *J* = 8.8 Hz, 4H), 6.96 (d, *J* = 8.8 Hz, 4H), 3.89 (s, 6H). **^{13}C NMR** (150 MHz, $CDCl_3$, 298 K): δ ppm 194.61, 162.99, 132.38, 130.94, 113.61, 55.62. **HR-MS** (EI) m/z: calcd for $C_{15}H_{14}O_3$ [M^+]: 242.0943, found: 242.0953.

1,1,2,2-tetrakis(4-methoxyphenyl)ethane (4). Based on a literature procedure,[2] $TiCl_4$ (1.91 mL, 17.42 mmol) was added dropwise to a solution of **3** (1.2789g, 5.28 mmol) and zinc powder (1.67 g, 25.87 mmol) in THF (50 mL) at room temperature under inert atmosphere. The mixture was refluxed until all the starting material was consumed (2 h). After cooling to room temperature, reaction mixture was hydrolysed by the addition of H_2O (50 mL). After removing most of the THF in the rotavapor, the resulting mixture was extracted with CH_2Cl_2. Combined organic phases were dried over anhydrous $MgSO_4$, filtered and concentrated in vacuo. The crude mixture was purified by flash chromatography (SiO_2, ethyl acetate/hexane, 15:85) to provide product **4** (0.84 g, 71%). **^{1}H NMR** (500 MHz, $CDCl_3$, 298 K): δ ppm 6.92 (d, *J* = 8.7 Hz, 8H), 6.63 (d, *J* = 8.7 Hz, 8H),

3.74 (s, 12H). **^{13}C NMR** (150 MHz, $CDCl_3$, 298 K): δ ppm 157.93, 138.52, 137.05, 132.68, 113.17, 55.23. **HR-MS** (EI) m/z: calcd for $C_{30}H_{28}O_4$ [M^+]: 452.1988, found: 452.1995.

4,4',4'',4'''-(ethene-1,1,2,2-tetrayl)tetraphenol (5). Based on a literature procedure,[3] **4** (1.5029 g, 3.32 mmol) was solved in CH_2Cl_2 (22 mL) and cooled with an ice-salt bath. Then, a 1 M CH_2Cl_2 solution of BBr_3 (13.28 mL, 13.28 mmol) was added dropwise under inert atmosphere. After the addition was completed, the cooling bath was removed, and the resulting solution was stirred at room temperature overnight. Then the reaction mixture was hydrolysed by dropwise addition of H_2O (11 mL). The precipitate was collected by filtration, washed with H_2O and solved in MeOH. The obtained crude was purified by flash chromatography (SiO_2, methanol/dichloromethane, 10:90) to provide product **5** (0.83 g, 90%).**^{1}H NMR** (500 MHz, DMSO, 298 K): δ ppm 9.28 (s, 4H), 6.69 (d, *J* = 8.5 Hz, 8H), 6.47 (d, *J* = 8.5 Hz, 8H). **^{13}C NMR** (150 MHz, DMSO, 298 K): δ ppm 155.44, 137.84, 135.21, 132.08, 114.62. **HR-MS** (EI) m/z: calcd for $C_{26}H_{20}O_4$ [M^+]: 396.1362, found: 396.1362.

Ethene-1,1,2,2-tetrayltetrakis(benzene-4,1-diyl) tetraacrylate (6). To a solution of **5** (1.0417 g, 2.63 mmol) in DMF (25 mL) at 0 °C thanks to an ice-water bath, was added TEA (2.55 mL, 18.41 mmol) and then acryloyl chloride (1.3 mL, 15.78 mmol) dropwise under inert atmosphere. After removal of the cooling bath, the resulting solution was stirred at room temperature overnight. Then a mixture of water/hexane 50:50 (10 mL) was added. An orange solid was formed, and it was filtered and washed with more water/hexane. Solid was redissolved in dichloromethane, dried and then purified by flash chromatography (SiO_2, ethyl acetate/hexane, 20:80) to provide product **6** (0.54 g, 33%). **^{1}H NMR** (500 MHz, $CDCl_3$, 298 K): δ ppm 7.05 (d, J = 8.6 Hz, 8H), 6.92 (d, J = 8.6 Hz, 8H), 6.57 (dd, *J* = 17.3 Hz, *J* = 1.1 Hz, 4H), 6.23 (dd, *J* = 17.3 Hz, *J* = 10.4 Hz, 4H), 5.99 (dd, *J* = 10.4 Hz, *J* = 1.1 Hz, 4H). **^{13}C NMR** (150 MHz, $CDCl_3$, 298 K): δ ppm 164.43, 149.44, 140.85, 139.95, 132.60, 132.50, 128.12, 121.05. **HR-MS** (EI) m/z: calcd for $C_{36}H_{28}O_8$ [M^+]: 612.1784, found: 612.1777.

Ethene - 1, 1, 2, 2 – tetrayltetrakis (benzene-4,1-diyl) tetrakis (3-(tritylthio)propanoate) (7). Based on a procedure optimized by the group,[4] compound **6** (0.1000 g, 0.16 mmol) was solved in CH_2Cl_2 (2 mL) under inert atmosphere. Then triphenylmethanethiol (TrSH) (0.2027 g, 0.73 mmol) and DBU (0.1 mL, 0.65 mmol) were added. After 1 min of reaction, 2 mL of NH_4Cl is added and the aqueous phase was extracted with CH_2Cl_2. The combined organic phases were dried over anhydrous $MgSO_4$, filtered and concentrated in vacuo. The crude mixture was purified by flash chromatography (SiO_2, ethyl acetate/hexane, 30:70) to provide the final product (174.7 mg, 62%). **^{1}H NMR** (500 MHz, $CDCl_3$, 298 K): δ ppm 7.45-7.44 (m, 24H), 7.30-7.27 (m, 24H), 7.23-7.20 (m, 12H), 6.96 (d, *J* = 8.3 Hz, 8H), 6.80 (d, *J* = 8.3 Hz, 8H), 2.54 (t, *J* = 7.2 Hz, 8H), 2.41 (t, *J* = 7.2 Hz, 8H). **^{13}C NMR** (150 MHz, $CDCl_3$, 298 K): δ ppm 170.15, 149.33, 144.71, 140.74, 139.74, 132.38, 129.71, 128.10, 126.87, 120.98, 67.08, 33.87, 26.92. **HR-MS** (EI) m/z: calcd for $C_{114}H_{92}O_8S_4Na$ [$M+Na]^+$: 1739.57, found: 1739.5716.

Ethene – 1, 1, 2, 2 – tetrayltetrakis (benzene – 4, 1 - diyl) tetrakis (3 - mercaptopropanoate) (8). Compound **7** (1.0697 g, 0.62 mmol) was solved in CH_2Cl_2 (12 mL) under inert atmosphere and

brought to 0 °C. Then, TFA (10.0 mL, 130.6 mmol) was added. The mixture was stirred for 5 min. Then Et_3SiH (0.79 mL, 4.96 mmol) was added and stirred again for another 5 min. The resulting mixture was concentrated in vacuo. The residue was solved in CH_2Cl_2 and washed with a saturated aqueous solution of $NaHCO_3$. The organic phase was dried with $MgSO_4$, filtered and the solvent was removed in the rotary evaporator. The crude was purified by flash chromatography (SiO_2. First: ethyl acetate/hexane, 30:70. Later: methanol/dichloromethane, 1:99) to give the corresponding product **8** (408.6 mg, 88%). **^{1}H NMR** (500 MHz, $CDCl_3$, 298 K): δ ppm 7.01 (d, *J* = 8.6 Hz, 8H), 6.87 (d, *J* = 8.6 Hz, 8H), 2.87-2.83 (m, 16H), 1.72-1.69 (m, 4H). **^{13}C NMR** (150 MHz, $CDCl_3$, 298 K): δ ppm 170.05, 149.34, 140.85, 139.83, 132.47, 121.06, 38.71, 19.80. **HR-MS** (EI) m/z: calcd for $C_{38}H_{36}O_8S_4$ [M^+]: 748.1293, found: 748.1281.

3. Synthesis and Characterization of Cages

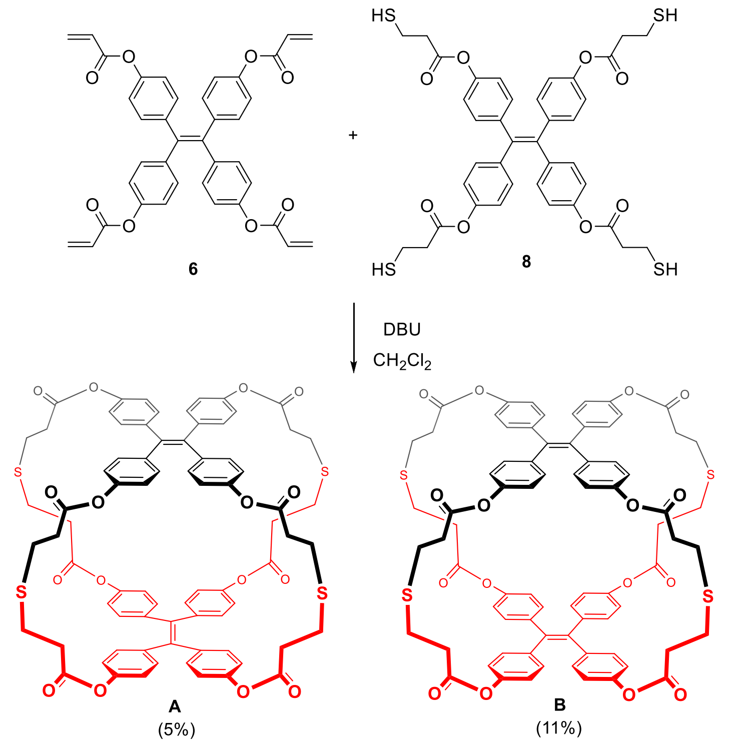

Figure S2. Synthesis of cages

Based on a procedure optimized by the group,[4] compound **8** (0.0660 g, 0.09 mmol) was solved in CH_2Cl_2 (18 mL) under inert atmosphere. Then **6** (0.0551 g, 0.09 mmol) and DBU (0.03 mL, 0.18 mmol) were sequentially added. After 1 min of reaction, 18 mL of a saturated aqueous solution of NH_4Cl were added and the aqueous phase was extracted with CH_2Cl_2. Combined organic phases were dried over anhydrous $MgSO_4$, filtered and concentrated in vacuo. The obtained crude mixture was purified by flash chromatography (SiO_2, ethyl acetate/hexane, 50:50) to provide the two isomers of the cage (Cage **A**, less polar: 6.5 mg, 5 %; Cage **B**, more polar: 13.0 mg, 11 %).

Cage A: **^{1}H NMR** (500 MHz, $CDCl_3$, 298 K): δ ppm 6.90 (d, *J* = 8.5 Hz, 16H), 6.82 (d, *J* = 8.5 Hz, 16H), 2.96 (t, *J* = 6.1 Hz, 16H), 2.83 (t, *J* = 6.1 Hz, 16H). **^{13}C NMR** (150 MHz, $CDCl_3$, 298 K): δ ppm 170.49, 149.52, 140.55, 139.10, 132.21, 121.45, 34.44, 27.64. **HR-MS** (ESI) m/z: calcd for $C_{76}H_{64}O_{16}S_4Na$ [$M+Na]^+$: 1383.2975, found: 1383.2982.

Cage B: ^{1}**H NMR** (500 MHz, $CDCl_3$, 298 K): δ ppm 6.91 (d, *J* = 8.5 Hz, 16H), 6.80 (d, *J* = 8.5 Hz, 16H), 2.99-2.93 (m, 16H), 2.90-2.84 (m, 16H). ^{13}**C NMR** (150 MHz, $CDCl_3$, 298 K): δ ppm 170.43, 149.50, 140.53, 139.82, 132.26, 121.51, 34.47, 26.78. **HR-MS** (ESI) *m/z*: calcd for $C_{76}H_{64}O_{16}S_4Na$ $[M+Na]^+$: 1383.2975, found: 1383.2961.

4. X-ray structure determination

Cage **B** crystallizes in the monoclinic crystal system with space group *P*21/c. As noted in the main text of the paper, the connectivity of the structure was determined unambiguously despite the low quality of the crystals and difficulties with the refinement.

Despite several crystallization attempts, all crystals obtained for Cage **B** were of very low quality and very weakly diffracting. Figure S3 shows the structure of the best of five datasets collected from three different samples. The diffraction intensity dropped off sharply as a function of the diffraction angle, so that I/sigma(I) was well below 3.0 at around 1.0 Å resolution. The data was therefore truncated to 0.92 Å, resulting in a low-precision structure, as reflected in the A alert in the checkcif shown on page S7. One of the S atoms was disordered and was refined over two positions. An incipient disorder seems to be also present in some further S atoms and ester groups resulting in rather elongated ADPs. RIGU and SADI restraints were used to keep a sensible geometry and the ADPs to a reasonable value. Although the final residual values are quite high (R1 = 0.1348 ($I > 2\sigma(I)$) and wR2 = 0.4486 for all reflections), the molecular geometry is shown unambiguously and clearly demonstrates the formation of the cage.

The checkcif file is shown on page S7 and the CIF file can be obtained from the authors on request.

Crystal Data for $C_{76}H_{64}O_{16}S_4$ (M =1361.51 g/mol): monoclinic, space group *P*21/c (no. 14), a = 14.6562(10) Å, b = 18.5340(11) Å, c = 25.141(2) Å, β = 95.367(7)°, V = 6799.2(8) Å3, Z = 4, T = 293(2) K, μ(CuKα) = 1.860 mm-1, Dcalc = 1.330 g/cm3, 19789 reflections measured (7.064° ≤ 2Θ ≤ 113.982°), 9129 unique (Rint = 0.0588, Rsigma = 0.0771) which were used in all calculations. The final R1 was 0.1348 ($I > 2\sigma(I)$) and wR2 was 0.4486 (all data)

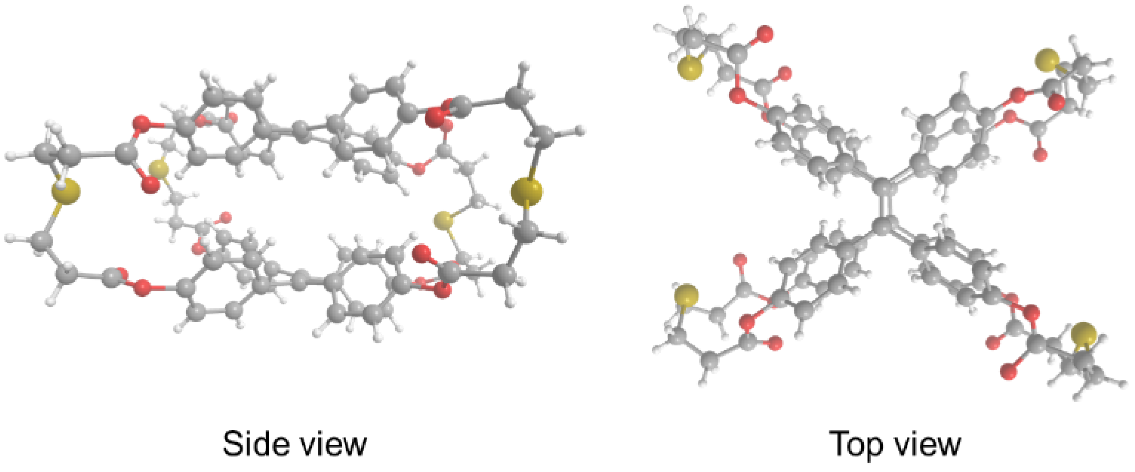

Figure S3. Structure of cage **B**

checkCIF/PLATON report

Structure factors have been supplied for datablock(s) exp_356

THIS REPORT IS FOR GUIDANCE ONLY. IF USED AS PART OF A REVIEW PROCEDURE FOR PUBLICATION, IT SHOULD NOT REPLACE THE EXPERTISE OF AN EXPERIENCED CRYSTALLOGRAPHIC REFEREE.

No syntax errors found. CIF dictionary Interpreting this report

Datablock: exp_356

Bond precision:	C-C = 0.0181 A		Wavelength=1.54184
Cell:	a=14.6562(10)	b=18.5340(11)	c=25.141(2)
	alpha=90	beta=95.367(7)	gamma=90
Temperature:	293 K		

	Calculated	Reported
Volume	6799.3(8)	6799.2(8)
Space group	P 21/c	P 1 21/c 1
Hall group	-P 2ybc	-P 2ybc
Moiety formula	C76 H64 O16 S4	C76 H64 O16 S4
Sum formula	C76 H64 O16 S4	C76 H64 O16 S4
Mr	1361.52	1361.51
Dx,g cm-3	1.330	1.330
Z	4	4
Mu (mm-1)	1.860	1.860
F000	2848.0	2848.0
F000'	2861.72	
h,k,lmax	15,20,27	15,20,27
Nref	9149	9129
Tmin,Tmax	0.768,0.847	0.772,1.000
Tmin'	0.695	

Correction method= # Reported T Limits: Tmin=0.772 Tmax=1.000
AbsCorr = GAUSSIAN

Data completeness= 0.998 Theta(max)= 56.991

R(reflections)= 0.1348(3075) wR2(reflections)= 0.4486(9129)

S = 1.094 Npar= 875

The following ALERTS were generated. Each ALERT has the format
test-name_ALERT_alert-type_alert-level.
Click on the hyperlinks for more details of the test.

Alert level A

```
THETM01_ALERT_3_A  The value of sine(theta_max)/wavelength is less than 0.550
            Calculated sin(theta_max)/wavelength =    0.5439
```

Alert level B

```
PLAT026_ALERT_3_B Ratio Observed / Unique Reflections (too) Low ..        34% Check
PLAT084_ALERT_3_B High wR2 Value (i.e. > 0.25) ..................       0.45 Report
PLAT230_ALERT_2_B Hirshfeld Test Diff for    S3        --C25      .       10.0 s.u.
PLAT230_ALERT_2_B Hirshfeld Test Diff for    S4        --C5       .        7.2 s.u.
PLAT241_ALERT_2_B High   'MainMol' Ueq as Compared to Neighbors of        C52 Check
PLAT241_ALERT_2_B High   'MainMol' Ueq as Compared to Neighbors of        C53 Check
PLAT241_ALERT_2_B High   'MainMol' Ueq as Compared to Neighbors of        C70 Check
PLAT340_ALERT_3_B Low Bond Precision on  C-C Bonds ...............    0.01811 Ang.
```

Alert level C

```
PLAT082_ALERT_2_C High R1 Value ..................................       0.13 Report
PLAT220_ALERT_2_C NonSolvent   Resd 1  C   Ueq(max)/Ueq(min) Range        3.1 Ratio
PLAT234_ALERT_4_C Large Hirshfeld Difference S1        --C71      .       0.20 Ang.
PLAT234_ALERT_4_C Large Hirshfeld Difference S2B       --C53      .       0.24 Ang.
PLAT234_ALERT_4_C Large Hirshfeld Difference O2        --C50      .       0.18 Ang.
PLAT234_ALERT_4_C Large Hirshfeld Difference O14       --C68      .       0.20 Ang.
PLAT234_ALERT_4_C Large Hirshfeld Difference C9        --C14      .       0.19 Ang.
PLAT234_ALERT_4_C Large Hirshfeld Difference C17       --C21      .       0.16 Ang.
PLAT234_ALERT_4_C Large Hirshfeld Difference C29       --C31      .       0.17 Ang.
PLAT234_ALERT_4_C Large Hirshfeld Difference C36       --C43      .       0.17 Ang.
PLAT234_ALERT_4_C Large Hirshfeld Difference C51       --C52      .       0.24 Ang.
PLAT234_ALERT_4_C Large Hirshfeld Difference C53       --C54      .       0.22 Ang.
PLAT234_ALERT_4_C Large Hirshfeld Difference C58       --C59      .       0.16 Ang.
PLAT234_ALERT_4_C Large Hirshfeld Difference C62       --C63      .       0.24 Ang.
PLAT234_ALERT_4_C Large Hirshfeld Difference C64       --C67      .       0.21 Ang.
PLAT234_ALERT_4_C Large Hirshfeld Difference C71       --C72      .       0.23 Ang.
PLAT234_ALERT_4_C Large Hirshfeld Difference C74       --C75      .       0.16 Ang.
PLAT234_ALERT_4_C Large Hirshfeld Difference C76       --C77      .       0.18 Ang.
PLAT234_ALERT_4_C Large Hirshfeld Difference C77       --C78      .       0.19 Ang.
PLAT241_ALERT_2_C High   'MainMol' Ueq as Compared to Neighbors of         O1 Check
PLAT241_ALERT_2_C High   'MainMol' Ueq as Compared to Neighbors of         O3 Check
PLAT241_ALERT_2_C High   'MainMol' Ueq as Compared to Neighbors of         O5 Check
PLAT241_ALERT_2_C High   'MainMol' Ueq as Compared to Neighbors of         O7 Check
PLAT241_ALERT_2_C High   'MainMol' Ueq as Compared to Neighbors of        O14 Check
PLAT241_ALERT_2_C High   'MainMol' Ueq as Compared to Neighbors of        O15 Check
PLAT241_ALERT_2_C High   'MainMol' Ueq as Compared to Neighbors of         C6 Check
PLAT241_ALERT_2_C High   'MainMol' Ueq as Compared to Neighbors of        C24 Check
PLAT242_ALERT_2_C Low    'MainMol' Ueq as Compared to Neighbors of         S1 Check
PLAT242_ALERT_2_C Low    'MainMol' Ueq as Compared to Neighbors of         C2 Check
PLAT242_ALERT_2_C Low    'MainMol' Ueq as Compared to Neighbors of         C8 Check
PLAT242_ALERT_2_C Low    'MainMol' Ueq as Compared to Neighbors of         C9 Check
PLAT242_ALERT_2_C Low    'MainMol' Ueq as Compared to Neighbors of        C20 Check
PLAT242_ALERT_2_C Low    'MainMol' Ueq as Compared to Neighbors of        C23 Check
```

```
PLAT242_ALERT_2_C Low    'MainMol' Ueq as Compared to Neighbors of        C29 Check
PLAT242_ALERT_2_C Low    'MainMol' Ueq as Compared to Neighbors of        C30 Check
PLAT242_ALERT_2_C Low    'MainMol' Ueq as Compared to Neighbors of        C33 Check
PLAT242_ALERT_2_C Low    'MainMol' Ueq as Compared to Neighbors of        C44 Check
PLAT242_ALERT_2_C Low    'MainMol' Ueq as Compared to Neighbors of        C50 Check
PLAT242_ALERT_2_C Low    'MainMol' Ueq as Compared to Neighbors of        C51 Check
PLAT242_ALERT_2_C Low    'MainMol' Ueq as Compared to Neighbors of        C55 Check
PLAT242_ALERT_2_C Low    'MainMol' Ueq as Compared to Neighbors of        C56 Check
PLAT242_ALERT_2_C Low    'MainMol' Ueq as Compared to Neighbors of        C62 Check
PLAT242_ALERT_2_C Low    'MainMol' Ueq as Compared to Neighbors of        C67 Check
PLAT242_ALERT_2_C Low    'MainMol' Ueq as Compared to Neighbors of        C68 Check
PLAT242_ALERT_2_C Low    'MainMol' Ueq as Compared to Neighbors of        C69 Check
PLAT242_ALERT_2_C Low    'MainMol' Ueq as Compared to Neighbors of        C73 Check
PLAT260_ALERT_2_C Large Average Ueq of Residue Including        S1      0.148 Check
PLAT309_ALERT_2_C Single Bonded Oxygen (C-O > 1.3 Ang) ..........         O8 Check
PLAT334_ALERT_2_C Small Aver. Benzene C-C Dist C9       -C14             1.36 Ang.
PLAT334_ALERT_2_C Small Aver. Benzene C-C Dist C17      -C21             1.37 Ang.
PLAT334_ALERT_2_C Small Aver. Benzene C-C Dist C44      -C49             1.36 Ang.
PLAT334_ALERT_2_C Small Aver. Benzene C-C Dist C62      -C65             1.37 Ang.
PLAT334_ALERT_2_C Small Aver. Benzene C-C Dist C74      -C79             1.37 Ang.
PLAT360_ALERT_2_C Short  C(sp3)-C(sp3) Bond  C69      - C70      .       1.43 Ang.
PLAT369_ALERT_2_C Long   C(sp2)-C(sp2) Bond  C12      - C15      .       1.53 Ang.
PLAT906_ALERT_3_C Large K Value in the Analysis of Variance ......    113.875 Check
PLAT906_ALERT_3_C Large K Value in the Analysis of Variance ......      4.364 Check
PLAT906_ALERT_3_C Large K Value in the Analysis of Variance ......     14.453 Check
PLAT906_ALERT_3_C Large K Value in the Analysis of Variance ......      2.031 Check
PLAT906_ALERT_3_C Large K Value in the Analysis of Variance ......      3.534 Check
PLAT911_ALERT_3_C Missing FCF Refl Between Thmin & STh/L=    0.544         19 Report
PLAT934_ALERT_3_C Number of (Iobs-Icalc)/Sigma(W) > 10 Outliers ..          1 Check
```

Alert level G

```
PLAT002_ALERT_2_G Number of Distance or Angle Restraints on AtSite         12 Note
PLAT072_ALERT_2_G SHELXL First  Parameter in WGHT  Unusually Large       0.20 Report
PLAT176_ALERT_4_G The CIF-Embedded .res File Contains SADI Records          1 Report
PLAT187_ALERT_4_G The CIF-Embedded .res File Contains RIGU Records          3 Report
PLAT199_ALERT_1_G Reported _cell_measurement_temperature ..... (K)        293 Check
PLAT200_ALERT_1_G Reported   _diffrn_ambient_temperature ..... (K)        293 Check
PLAT230_ALERT_2_G Hirshfeld Test Diff for    S2A      --C52      .        9.0 s.u.
PLAT230_ALERT_2_G Hirshfeld Test Diff for    S2A      --C53      .        8.0 s.u.
PLAT301_ALERT_3_G Main Residue  Disorder .............(Resd  1  )          1% Note
PLAT410_ALERT_2_G Short Intra H...H Contact  H51A     ..H52D     .       1.98 Ang.
                                                      x,y,z  =      1_555 Check
PLAT410_ALERT_2_G Short Intra H...H Contact  H51A     ..H52A     .       2.14 Ang.
                                                      x,y,z  =      1_555 Check
PLAT410_ALERT_2_G Short Intra H...H Contact  H51B     ..H52C     .       1.99 Ang.
                                                      x,y,z  =      1_555 Check
PLAT410_ALERT_2_G Short Intra H...H Contact  H53D     ..H54A     .       2.14 Ang.
                                                      x,y,z  =      1_555 Check
PLAT860_ALERT_3_G Number of Least-Squares Restraints ............         153 Note
PLAT910_ALERT_3_G Missing # of FCF Reflection(s) Below Theta(Min).          2 Note
PLAT941_ALERT_3_G Average HKL Measurement Multiplicity ..........         2.2 Low
PLAT978_ALERT_2_G Number C-C Bonds with Positive Residual Density.          0 Info
```

1 **ALERT level A** = Most likely a serious problem - resolve or explain
8 **ALERT level B** = A potentially serious problem, consider carefully

```
 62 ALERT level C = Check. Ensure it is not caused by an omission or oversight
 17 ALERT level G = General information/check it is not something unexpected

  2 ALERT type 1 CIF construction/syntax error, inconsistent or missing data
 52 ALERT type 2 Indicator that the structure model may be wrong or deficient
 15 ALERT type 3 Indicator that the structure quality may be low
 19 ALERT type 4 Improvement, methodology, query or suggestion
  0 ALERT type 5 Informative message, check
```

It is advisable to attempt to resolve as many as possible of the alerts in all categories. Often the minor alerts point to easily fixed oversights, errors and omissions in your CIF or refinement strategy, so attention to these fine details can be worthwhile. In order to resolve some of the more serious problems it may be necessary to carry out additional measurements or structure refinements. However, the purpose of your study may justify the reported deviations and the more serious of these should normally be commented upon in the discussion or experimental section of a paper or in the "special_details" fields of the CIF. checkCIF was carefully designed to identify outliers and unusual parameters, but every test has its limitations and alerts that are not important in a particular case may appear. Conversely, the absence of alerts does not guarantee there are no aspects of the results needing attention. It is up to the individual to critically assess their own results and, if necessary, seek expert advice.

Publication of your CIF in IUCr journals

A basic structural check has been run on your CIF. These basic checks will be run on all CIFs submitted for publication in IUCr journals (*Acta Crystallographica*, *Journal of Applied Crystallography*, *Journal of Synchrotron Radiation*); however, if you intend to submit to *Acta Crystallographica Section C* or *E* or *IUCrData*, you should make sure that full publication checks are run on the final version of your CIF prior to submission.

Publication of your CIF in other journals

Please refer to the *Notes for Authors* of the relevant journal for any special instructions relating to CIF submission.

Validation response form

Please find below a validation response form (VRF) that can be filled in and pasted into your CIF.

```
# start Validation Reply Form
_vrf_THETM01_exp_356
;
PROBLEM: The value of sine(theta_max)/wavelength is less than 0.550
RESPONSE: ...
;
# end Validation Reply Form
```

PLATON version of 13/07/2021; check.def file version of 13/07/2021

Datablock exp_356 - ellipsoid plot

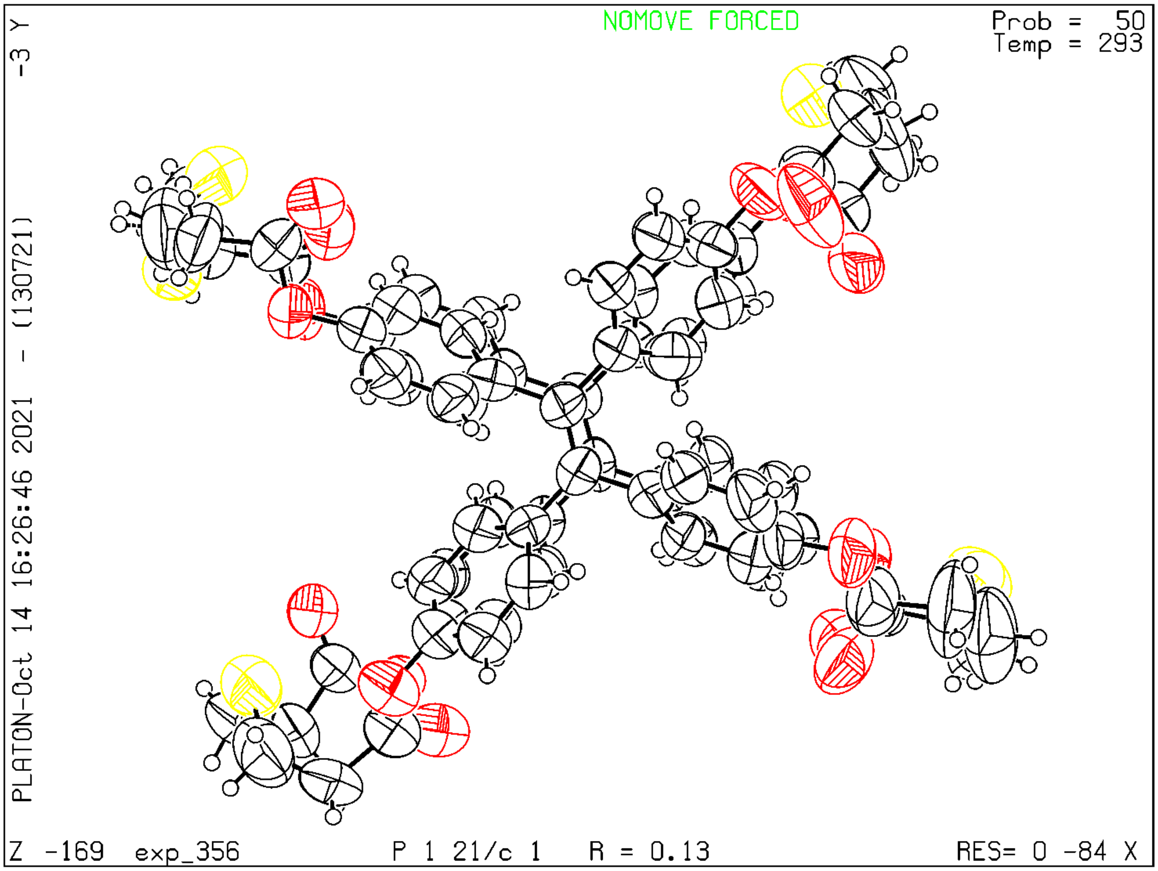

5. Photophysical properties of cages

5.1. Absorption

Solid samples of cage **A** and cage **B** were dissolved in 2 mL of THF/H_2O (2:8) mixture to obtain a final concentration of 10 µM. The molar absorption coefficient (ε) was obtained by the Lambert-Beer law:

$$A = \varepsilon c b \qquad \textbf{(1)}$$

Where A is the absorbance, c is the concentrations of cage (mol L^{-1}), and b is the thickness of the absorption layer (cm). In our case c was 1 cm. UV/Vis spectra of fluorescein 1 µM in H_2O and NaOH 0.1 M was also measured for quantum yield calculations. UV–Vis spectroscopy was performed on a Jasco-560 spectrophotometer.

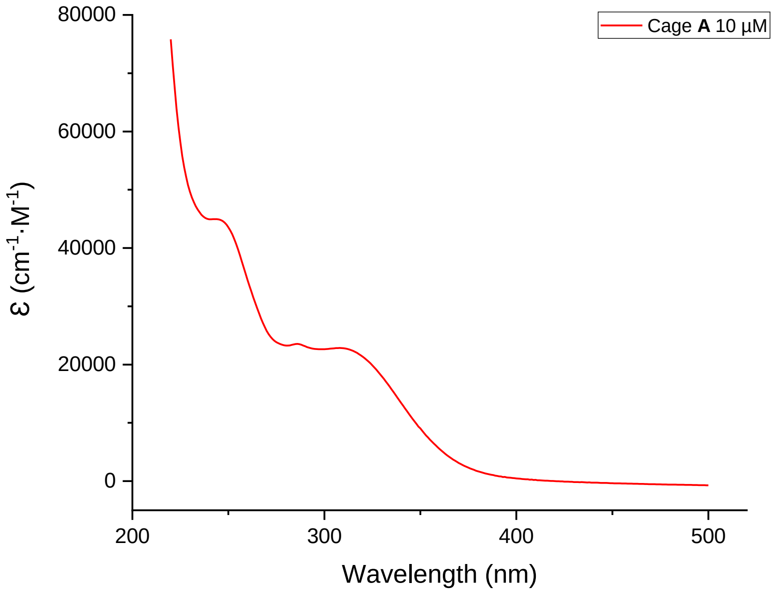

Figure S4. UV/Vis spectra of fluorescent cage **A** 10 µM in 2:8 THF/H_2O

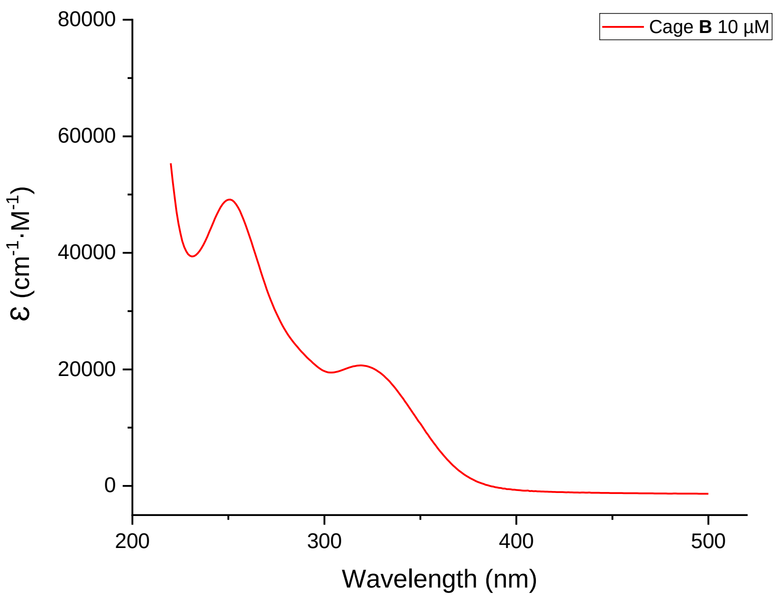

Figure S5. UV/Vis spectra of fluorescent cage **B** 10 µM in 2:8 THF/H_2O

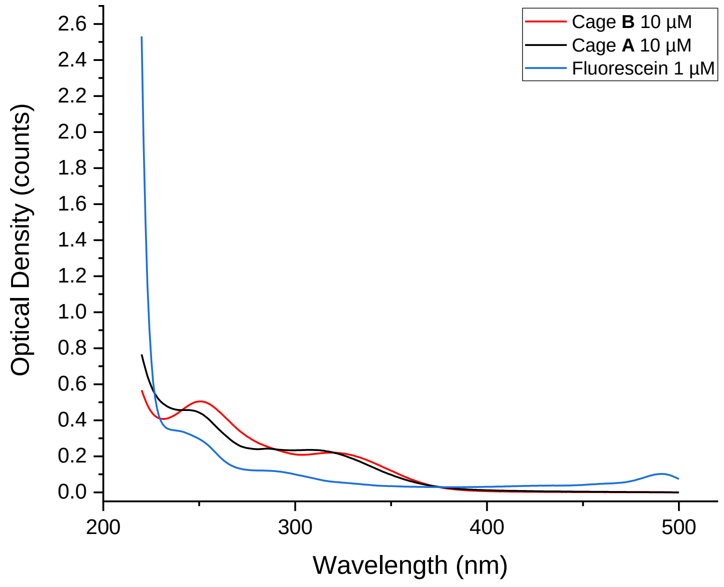

Figure S6. UV/Vis spectra of cage **A**, cage **B** and fluorescein 1 µM in H_2O and NaOH 0.1 M

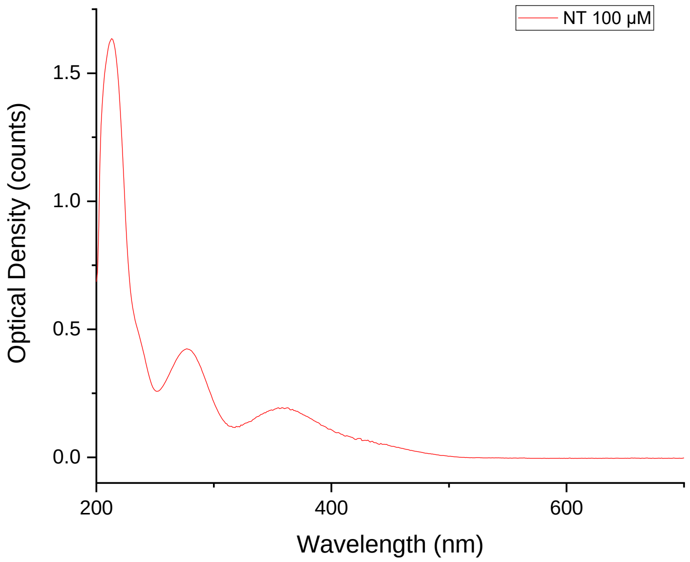

Figure S7. UV/Vis spectra of 3-nitrotyrosine (NT) 100 µM in 2:8 THF/H_2O

5.2. Emission

2 mL solutions (10 µM) of cages **A** and **B** were used to measure the fluorescence emission. Moreover, 2 mL of a solution of cage **B** at a concentration of 100 µM was also prepared. Emission spectra were recorded with the following acquisition parameters: λ_{exc}: 375 nm, step: 1 nm, integration time: 1s.

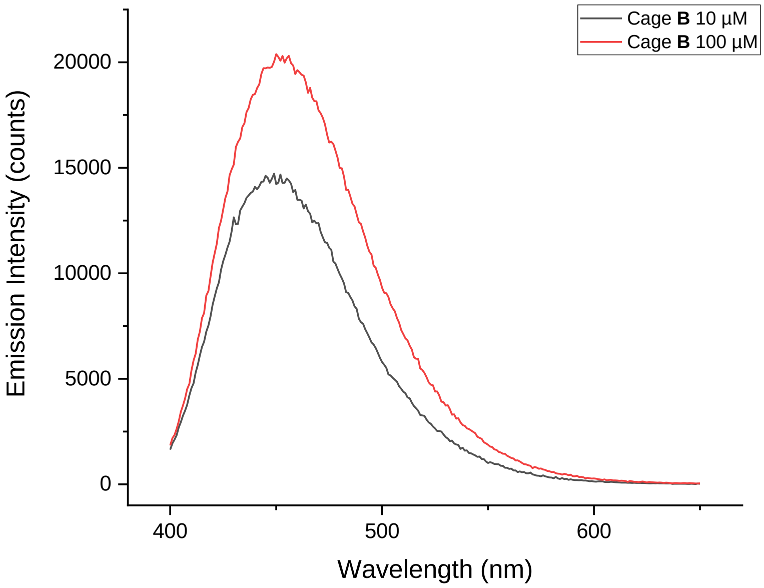

Figure S8. Fluorescence spectra of cage **B** 10 µM and 100 µM in 2:8 THF/H_2O

5.3. Quantum Yield

Solutions of cage **A** and **B** 10 µM each in 2 mL of THF/H_2O 2:8 were prepared. Quantum yields of cage **A** and **B** were calculated using fluorescein in NaOH 0,1 M as reference and the equation **2** with λ_{exc} = 375 nm, where Q is the quantum yield, I is the integrated fluorescence intensity, A is absorption at excitation wavelength and η is the refractive index of the solvent.

$$QY = QY_{ref}\left(\frac{\eta^2}{\eta_{ref}^{\ 2}}\right)\left(\frac{I}{A}\right)\left(\frac{A_{ref}}{I_{ref}}\right) \qquad \textbf{(2)}$$

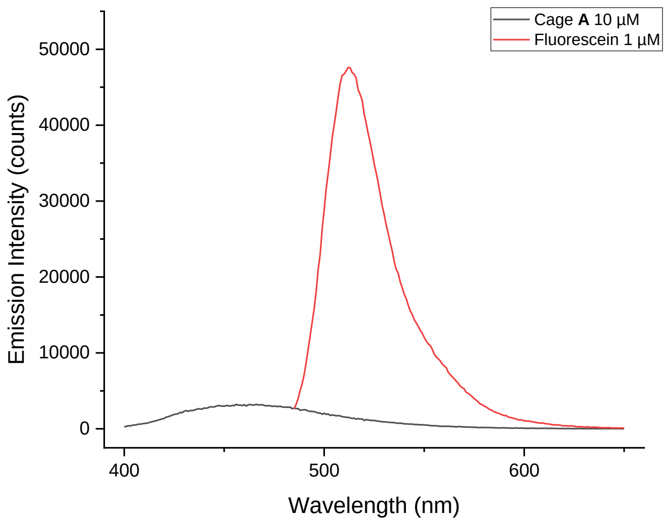

Figure S9. Fluorescence spectra of cage **A** 10 µM in 2:8 THF/H_2O and fluorescein 1 µM in H_2O and NaOH 0.1 M

**QY Cage A**	QYref	η	ηref	I	Iref	A	Aref
**0.2±0.03**	0.93±0.03	1.35	1.33	298446 ±2984	2030333 ±20303	0.023 ±0.0012	0.0322 ±0.0016

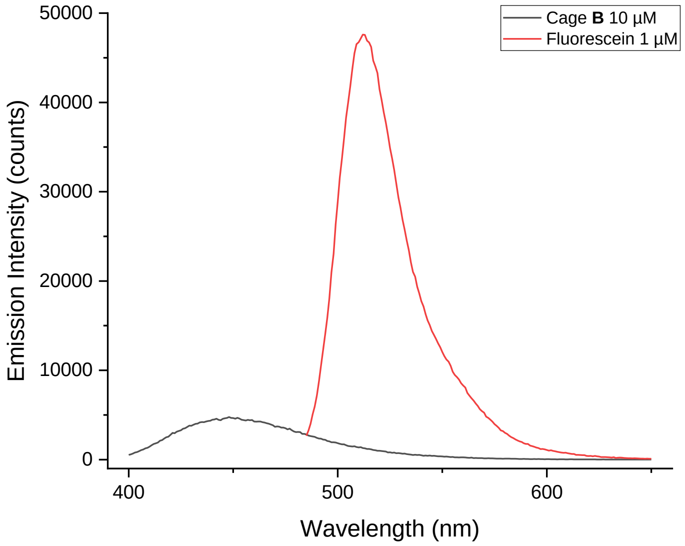

Figure S10. Fluorescence spectra of cage **B** 10 µM in 2:8 THF/H_2O and fluorescein 1 µM in H_2O and NaOH 0.1 M

**QY Cage B**	QYref	η	ηref	I	Iref	A	Aref
**0.4±0.06**	0.93±0.03	1.35	1.33	375182 ±3751	2030333 ±20303	0.0156 ±0.0008	0.0322 ±0.0016

5.4. Lifetime

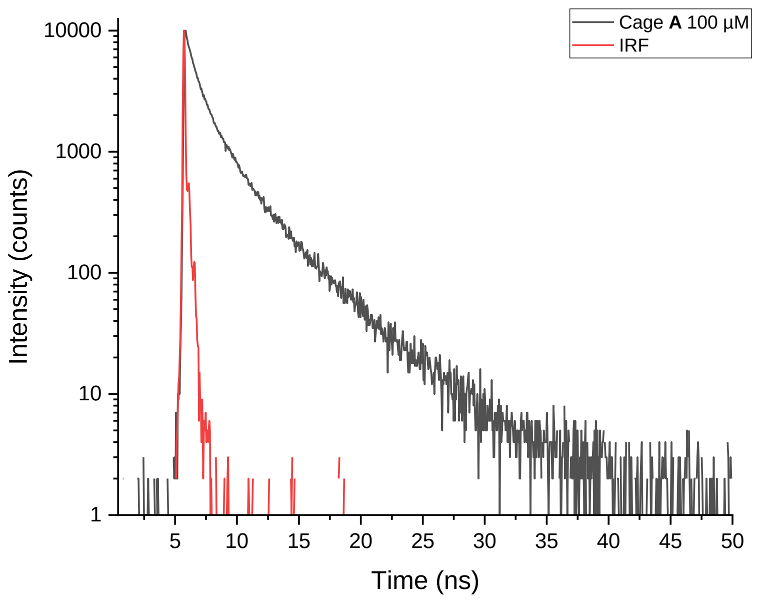

Figure S11. Fluorescence lifetime profile (excited at 375 nm) of cage **A** 100 µM in 2:8 THF/H_2O

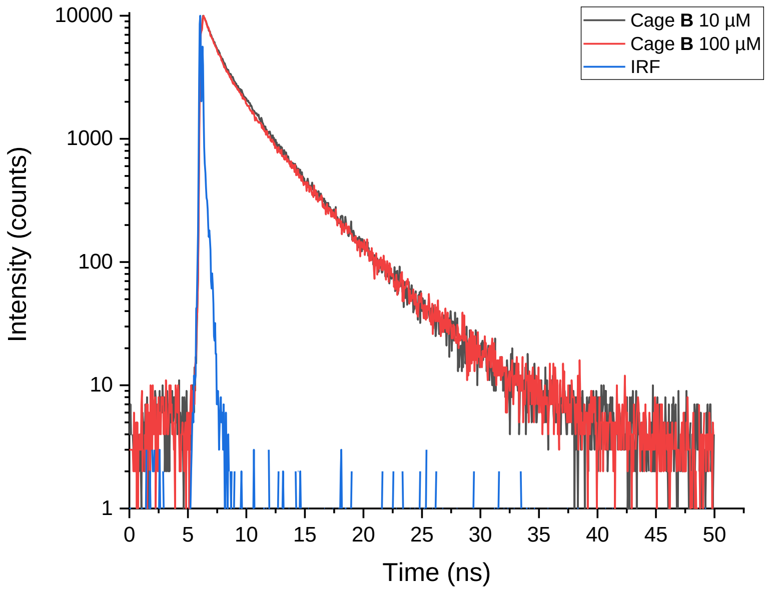

Figure S12. Fluorescence lifetime profile (excited at 375 nm) of cage **B** 10 µM and 100 µM in 2:8 THF/H_2O

The decay of the fluorescence of the cages was measured. A pulsed pump laser at 375 nm was selected for the optical excitation and the detection was tuned at the maximum of the emission

band, at around 450 nm. The fluorescence decay curve was not monoexponential. However, the decay curve could be fitted to a bi-exponential function of the type :

$$I(t) = A_1 e^{-t/\tau_1} + A_2 e^{-t/\tau_2} \quad \textbf{(3)}$$

Where, τ_1 and τ_2 are the decay constants and A_1 and A_2 represent the pre-exponential factors. The fitting was made using IRF reconvolution analysis with F900 software (Edinburgh Instruments) and leaving τ_1 , τ_2 , A_1 and A_2 as fitting parameters.

The best fitting for the decay curves of Cage **A** and **B** was obtained with the following parameters:

	A_1	τ_1 (ns)	A_2	τ_2 (ns)
Cage **A**	1 ±0.02	1 ± 0.005	0.175 ± 0.003	3.67 ± 0.016
Cage **B**	1 ±0.02	1.1 ± 0.006	0.669 ± 0.013	3.96 ± 0.02

An intensity averaged lifetime can be defined from the fitting parameters[5] as:

$$< \tau_{av} >= \frac{A_1 \tau_1^2 + A_2 \tau_2^2}{A_1 \tau_1 + A_2 \tau_2} \quad \textbf{(4)}$$

The average lifetimes of Cage **A** and **B** are 2.0 ± 0.2 ns and 3.0 ± 0.3 ns, respectively.

6. Binding of 3-nitrotyrosine to cage B determined by fluorescence quenching

Quenching was studied by increasing the concentration of the 3-nitrotyrosine (NT) quencher while keeping the concentration of cage **B** constant at 1 µM. The concentration of NT was increased from 0 µM to 25 µM in steps of 2.5 µM with a total of eleven different solutions with a final volume of 2 mL. The solvent used was 2:8 THF/H_2O. Fluorescence emission spectra were obtained in this case using the excitation wavelength at 320 nm.

The plot of F_0/F versus NT concentration allows us to obtain an association constant from the slope of the linear fit of 15990 ± 619.58 M^{-1} in 2:8 THF/H_2O.

6.1 Limit of detection based on the standard deviation of the blank

In order to obtain the σ value based on the blank, the integrated emission intensity of a blank sample (F_0) was independently measured 20 times:

1	3.4600
2	3.4810
3	3.4370
4	3.4650
5	3.4410
6	3.4190
7	3.4200

8	3.3920
9	3.4400
10	3.4660
11	3.4300
12	3.4680
13	3.4510
14	3.4110
15	3.4380
16	3.4360
17	3.4270
18	3.4220
19	3.4400
20	3.4210

Average 3.44

SD of F_0 0.02199

In order to calculate the standard deviation of the ratio F_0/F, and considering the propagation of errors, then the value obtained was $\sigma = 0.013$. Therefore, the limit of detection of cage **B** is LoD = 3µM rounding up to micromolar units.

6.2 Confirmation of the static quenching mechanism

There are two possible quenching mechanisms to explain the linear Stern-Volmer plots: dynamic quenching and static quenching. Dynamic quenching, also known as collisional quenching, occurs when the quencher diffuses to the fluorescent cage during the lifetime of its excited state. The excited cage returns to its ground state without emission of radiation due to contact with the quencher. That is, quenching occurs without any permanent change in the molecules. On the contrary, static quenching happens when a molecular complex is formed between the fluorescent cage and the quencher, and this new complex is nonfluorescent. In order to distinguish which of the two mechanisms is responsible of the cage fluorescence quenching, lifetime measurements can be performed as a function of the CD biomarker quencher.

In the case of dynamic quenching the lifetime of the excited state of the fluorophore decreases with the quencher concentration according to the following equation:

$$\frac{\tau_0}{\tau} = 1 + K_{SV}[Q] \qquad \textbf{(5)}$$

Consequently,

$$\frac{F_0}{F} = \frac{\tau_0}{\tau} \qquad \textbf{(6)}$$

Therefore, in the case of dynamic quenching a shortening of the lifetime at the same rate of the emission intensity would be expected as the 3-nitrotyrosine (NT) concentration increases.

On the other hand, if static quenching is occurring, a chemical reaction occurs, in which non-fluorescent cage-NT complexes are formed. In this case, the fluorescence detected comes from cages which have not interacted with the quencher and, consequently, their lifetime remains the same.

The decay of the emission intensity of cage **B** at a concentration of 10 µM was measured at different concentrations of NT. The lifetime remains practically constant with a value of τ = 2.40 ± 0.01 ns and negligible fluctuations. It can be concluded that quenching occurs only by static quenching.

6.3 UV-titration.

A UV-titration was performed to measure the association constant by an alternative method. Concentration of the 3-nitrotyrosine (NT) quencher was increased from 0 to 250 µM in THF/water 2:8, while keeping the concentration of cage B constant at 100 µM.

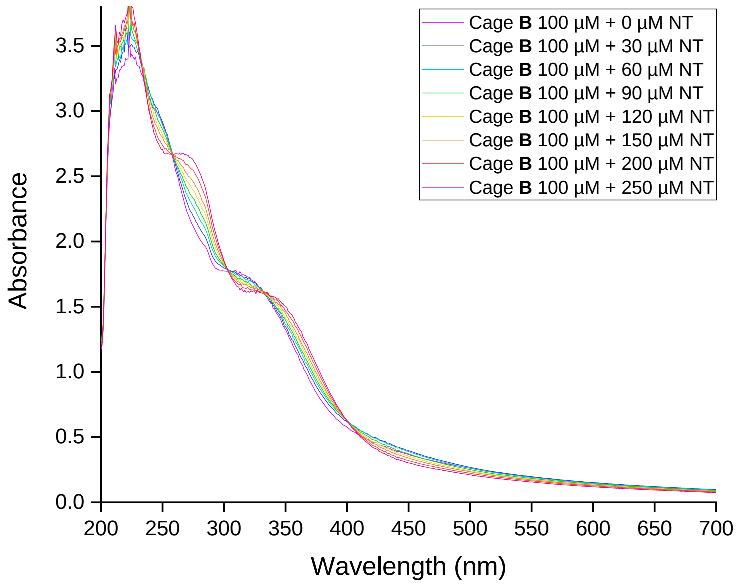

Figure S13. UV-titration of cage **B** 100 µM with NT in 2:8 THF/H_2O

Then, the Benesi-Hildebrand method was applied, and the association constant obtained was obtained by dividing the intercept by the slope of the regression, yielding $K_a = 1{\cdot}10^4$ M^{-1}

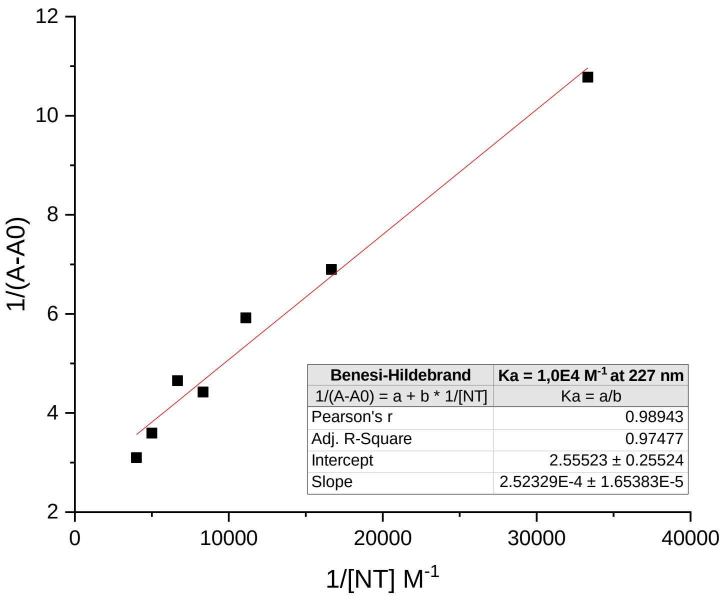

Figure S14. Benesi-Hildebrand method.

Additionally, we performed a non-linear regression by using the free software available at supramolecular.org,[6] and we obtained an even closer value of K_a =(1.45 ± 0.083)·10^4 M^{-1}

http://app.supramolecular.org/bindfit/view/9d6c8a64-5341-4a32-98a8-12dde8986b1d

7. Binding of 3-nitrotyrosine to cage B determined by fluorescence quenching in human serum

Three different human blood serums were purchased: One from Sigma-ALDRICH, one from SEQENS and another one from BIOWEST.

The complete description of each serum provided by each company can be found below:

-Human pooled serum, French origin

SEQENS

This human pooled serum is obtained from off-the-clot serum units collected in France. Units are pooled and 0.2 µm filtered prior to being bottled. Possibility of gender and blood group selection.

- Dry extract: 70 to 90 g/L
- Total proteins: 60 to 80 g/L
- Filtered through a 0.2 µm membrane
- Shelf life: 3 years

Donations used for the manufacture of this product have been collected in EU licensed donation facilities, in accordance with ethical requirements of the French Code de la santé publique.

Donor consent: donations proceed from volunteer donors, who agreed their donation may be used in any way the collection center deems appropriate.

Gratuitousness: donations have not been paid.

Anonymity: each donation is anonymous and is associated with a donation number.

Each unit has also been tested and found negative or non-reactive for: Anti-HIV 1+2 and anti-HCV antibodies, HBs antigen, HIV 1 and HCV RNA Syphilitic serology.

Complete donor traceability on file.

-Human Serum HIV tested (S4200)

BIOWEST

Collected from the source:

When searchers choose their serum, an important factor that should be taken into consideration is the source, which also emphasizes the traceability of the serum. Our system of vertical integration allows us to be certain of the origins and traceability of our human serum. The donors are volunteers. Each manufactured batch is rigorously controlled, from the collection of serum in authorized organisms, and throughout all stages of its treatment and production through to final packaging on the authorized organisms and on our premises. The serum is sourced from multiple blood types and multiple genders. The serum is off-the-clot serum, processed from human blood that has had natural coagulation. The serum is collected or imported and treated in agreement with the European regulations.

Filtration: Final Filter Size: 0.2µm x 2

Sterility: All sera are tested for the absence of aerobic and anaerobic bacteria, fungi and yeast. The sterility test is based on the European Pharmacopeia requirements.

Virus Tested: All of our human serum is tested for: - Hepatitis B antigen (HBs Ag) - Hepatitis C virus and antibodies (HCV) - HIV Type 1 virus and antibodies HIV ½ - Syphilis.

Endotoxin: All sera are tested to determine the levels of endotoxins.

BioWest carries out a chromokinetic quantitative test, according to the method D of the European Pharmacopoeia. The endotoxin reagent is standardized against the US reference endotoxin.

Haemoglobin: The haemoglobin level is measured by spectrophotometer.

Osmolality: Determined by a lowered freezing temperature. The osmometer is calibrated against standard solutions.

Cell Culture: Biological performance is assessed using cell culture medium supplemented with the serum being tested. During the test period, cultures are examined microscopically for any morphological abnormalities that may indicate toxic components in the serum.

Cell Lines Tested: MRC-5 - Human Fetal Lung HELA - Cancer Cell/Human.

Total Protein: Determined by Biuret Colorimetry.

Country of Origin: It is the country in which the serum was taken from the donor. BioWest sera are sourced from France, Germany, Poland or USA.

-Human Serum (from male AB clotted whole blood), USA origin, sterile-filtered (H6914)

SIGMA-ALDRICH

Product H6914 is prepared from whole blood that is allowed to clot, centrifuged and the serum removed. There is no additional processing required. Product H6914 will contain more growth factors since it is allowed to spontaneously clot and there will be the release of growth factors from many of the cells (WBC, platelets) present.

All donor units are collected in donor centers located in the United States, which are licensed by the FDA.

Appearance (Color) Colorless to Brown-Yellow to Brown.

Appearance (Form) Liquid pH 7.0 - 9.0 Iron (UG%) 40 - 100

Osmolality 260 - 340 Expressed in MOSM/KG H2O.

Sterility by USP Guidelines Pass.

Hemoglobin < 25 mg/dl

Mycoplasma Test None Detected.

Endotoxin Level < 10 EU/ml

Cholesterol 80 - 200 mg/dl

Triglyceride 30 - 175 mg/dl

Glucose 50 - 180 mg/dl

Sodium (Na) 100 - 160

MEQ/L Protein Content 4.0 - 9.0 %

Tested For Infectious Agents.

We prepared several stock solutions with different concentrations of cage **B** in DMSO, and 3-nitrotyrosine in PBS buffer (pH 7.4) to obtain final known concentrations of cage **B** (1, 2, 4, 8, 16, 32, 64, 128 µM) and NT (0, 4, 8, 16, 32, 64, 128 µM).

Solutions were prepared in 96-well black polystyrene plates from Thermo Scientific, 56 of which were used for each measurement. The data of each serum was distinguished by their commercial names: SEQENS, BIOWEST and SIGMA. In these 56 wells the maximum volume was always 100 µL and the 2:6:2 ratio of DMSO/buffer PBS 7,4/serum was always kept constant in each well.

In a first round of measurements, the fluorescence intensity data were taken for a plate with serum from SEQENS (SQ-1), another from SIGMA and another from BIOWEST. To reduce the variability of the data due to equipment repeatability, three measurements were made, one at time 0 min after preparation, another at 10 min and another at 20 min. In a second round of

measurements, intensity data were taken for three equal plates with SEQENS serum (SQ-2, SQ-3 and SQ-4). Again, 3 measurements were made for each plate (0, 10 and 20 min).

The assay was carried out in a FLUOStar Omega microplate reader (BMG Labtech) with an excitation filter with wavelength of 380 nm, and an emission filter with wavelength of 470 nm. Measures were made with top optic, 1 cycle and 3 flashes per well with each plate. Multichannel (eight) micropipette was used. The lecture of multiwell were made using Omega Control software and data processing was done using MARS 3.31 software. Fluorescence expected is shown in Figure S17.[7]

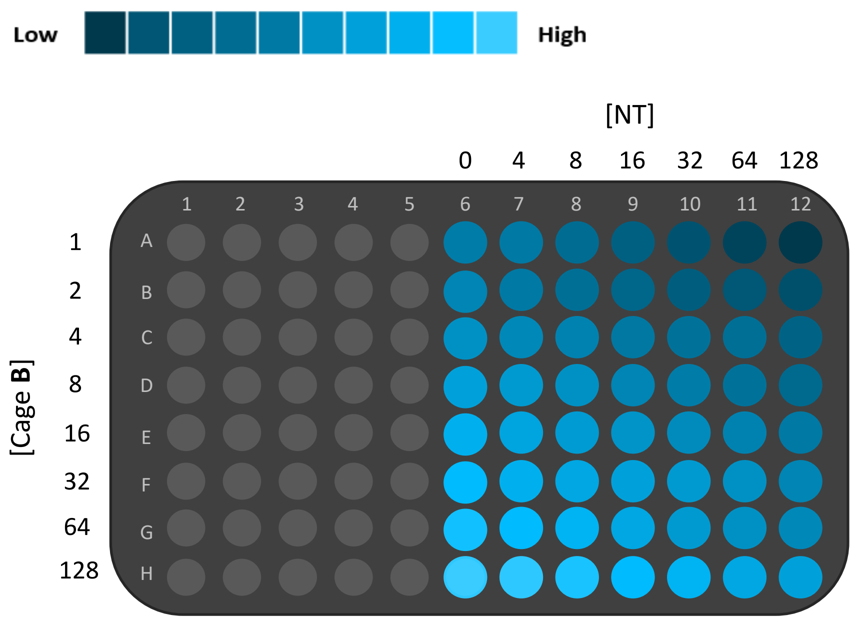

Figure S15. Fluorescence expected in the multiwell plate

The values of the intensity of fluorescence from each plate with different sera are collected in the next tables. Each table corresponds to one of the sera, and it shows the fluorescence intensities (at 0, 10 and 20 min) measured at 470 nm varying concentrations of cage **B** and NT.

SQ-1.1 Serum

[NT] µM; rows: [Cage B] µM

	0	4	8	16	32	64	128
1	9496	8768	10412	9962	8364	6434	7610
2	10802	11406	11753	11740	9094	9902	8766
4	13218	11550	11573	12573	11429	10553	9554
8	15664	16449	17488	14246	14534	13504	13594
16	24413	24629	25574	24000	22298	19317	18241
32	35188	44780	43965	40376	37900	35308	31361
64	70848	67925	71260	81580	68196	67858	59648
128	127189	130188	133809	126944	121722	123428	110809

SQ-1.2 Serum

[NT] µM; rows: [Cage B] µM

	0	4	8	16	32	64	128
1	9426	8790	9182	8680	8536	7057	7709
2	10168	11794	10302	11830	9480	9537	8728
4	13200	12092	12097	11604	11801	11082	10014
8	15592	16956	16290	15269	15024	14660	14413
16	24033	24042	25830	24877	23196	21429	19785
32	38545	45793	42637	41454	39134	36782	32680
64	69126	68744	72654	84288	69510	66884	61173
128	128148	131613	137021	129789	126562	126492	119390

SQ-1.3 Serum

[NT] µM; rows: [Cage B] µM

	0	4	8	16	32	64	128
1	9232	8806	9165	8620	8184	6973	7909
2	10214	11368	9726	12252	9564	9376	8716
4	12838	11981	11926	11750	12093	11212	10082
8	17330	16576	16621	15333	14952	14948	14713
16	24986	25369	26724	25153	22712	21409	19992
32	37769	46068	43790	42064	39598	37180	32370
64	69157	69321	72141	85206	69589	65608	62349
128	129186	129410	136018	132117	127936	126042	117660

SIGMA-1.1 Serum

[Cage B] μM \ [NT] μM	0	4	8	16	32	64	128
**1**	9881	9773	9265	9425	9129	8809	8649
**2**	10706	10909	10964	10837	10420	9904	8916
**4**	13090	13440	13104	13060	12498	11914	10490
**8**	16493	17140	17269	17773	16052	14496	14136
**16**	24497	24632	23476	24648	23668	20940	19129
**32**	38046	37274	37714	38408	36333	32624	29918
**64**	70926	62794	68424	72050	59949	56961	50269
**128**	128465	127256	134013	146982	111969	107848	93178

SIGMA-1.2 Serum

[Cage B] μM \ [NT] μM	0	4	8	16	32	64	128
**1**	9725	9562	9406	9206	8970	8464	7989
**2**	11070	10893	10872	10310	9932	9801	8738
**4**	13330	13012	13413	13222	12897	12252	10784
**8**	16857	17068	16952	17508	16926	14968	13830
**16**	25944	24261	24245	25352	24101	21625	19382
**32**	39206	37998	38998	40214	37618	34274	30429
**64**	70818	64626	68698	72265	61592	57837	50613
**128**	125326	123094	129480	140038	108516	106125	91610

SIGMA-1.3 Serum

[Cage B] μM \ [NT] μM	0	4	8	16	32	64	128
**1**	9637	9377	9309	9125	9062	8376	8208
**2**	10848	10460	10870	10770	10381	9480	8753
**4**	13474	13473	13632	13482	12849	12253	10697
**8**	17276	17181	16946	17605	16484	15280	14236
**16**	25292	25489	24493	25757	23418	22074	19680
**32**	39357	38468	39236	40976	37753	34556	31197
**64**	71534	65217	69270	72790	61730	57786	51378
**128**	126077	122725	131532	140785	110989	106521	90729

BIOWEST-1.1 Serum

[NT] µM

**[Cage B] µM**	**0**	**4**	**8**	**16**	**32**	**64**	**128**
**1**	9196	9494	9246	8952	9924	7978	8149
**2**	10378	9885	10334	10154	10268	8462	8374
**4**	12825	12056	13489	11448	14066	11158	10650
**8**	16344	16064	17418	14709	16257	13638	12108
**16**	24697	22972	23229	21780	20318	20102	18864
**32**	41116	38161	36656	36962	35562	31178	34460
**64**	74197	71556	63613	66509	66700	55910	57314
**128**	127190	136016	122624	126233	112629	106977	103588

BIOWEST-1.2 Serum

[NT] µM

**[Cage B] µM**	**0**	**4**	**8**	**16**	**32**	**64**	**128**
**1**	9208	9392	9432	8774	10186	7489	7861
**2**	10566	9844	10318	9790	10636	8218	8032
**4**	13070	12453	13697	12124	13628	10713	10720
**8**	16724	16328	17950	15256	16968	13393	12561
**16**	24469	24174	23872	22646	22065	20644	19626
**32**	41950	39688	38040	38762	42016	33325	35400
**64**	75501	74080	65849	68784	76713	58982	59109
**128**	127680	138938	126364	131810	123430	110364	109045

BIOWEST-1.3 Serum

[NT] µM

**[Cage B] µM**	**0**	**4**	**8**	**16**	**32**	**64**	**128**
**1**	8978	9270	9230	9040	10066	7528	7262
**2**	10354	9622	10322	9981	11094	8626	8070
**4**	13712	12245	13904	12509	13614	10657	10640
**8**	16784	16290	17573	14925	16112	13845	12692
**16**	25256	24656	24984	22590	23037	21081	20020
**32**	40662	40106	39113	37754	46262	34520	34733
**64**	75505	74790	67789	68576	87537	62162	60360
**128**	126752	139333	128253	133558	133510	116008	114793

SQ-2.1 Serum

[Cage B] μM \ [NT] μM	0	4	8	16	32	64	128
**1**	8964	9229	9110	9242	8753	8756	7833
**2**	10900	10561	10856	10588	10057	9872	8793
**4**	13340	13957	13080	12810	12512	12048	10952
**8**	17586	18061	16989	17150	16713	16601	15021
**16**	26872	25076	25732	25453	25054	25540	23094
**32**	50037	51604	45012	46040	41805	41913	36877
**64**	84486	78869	75029	78376	78714	73688	69848
**128**	131064	129173	136280	134956	149314	130128	135818

SQ-2.2 Serum

[Cage B] μM \ [NT] μM	0	4	8	16	32	64	128
**1**	9078	9009	9221	9172	8213	8252	7773
**2**	10188	10373	10361	10461	9861	9713	8866
**4**	12341	13314	12776	12849	12198	12193	10981
**8**	17264	17574	16653	16752	16237	16221	14934
**16**	26541	24845	25914	24985	24985	24573	22089
**32**	51997	48996	44518	45624	41837	43372	37341
**64**	81378	79541	75228	78344	77826	73678	68830
**128**	130036	129502	137426	135374	146993	129178	133085

SQ-2.3 Serum

[Cage B] μM \ [NT] μM	0	4	8	16	32	64	128
**1**	8989	8781	8972	8877	8517	8306	7780
**2**	10422	10128	10240	10577	9910	9992	8993
**4**	12512	12769	12880	12472	12218	12389	10602
**8**	16721	16896	17080	16745	16054	16750	14884
**16**	25953	25224	25730	25022	24917	24014	22774
**32**	40976	45045	40961	45624	41226	42808	36473
**64**	76514	77073	74110	78958	77757	74474	69080
**128**	130590	129162	138022	135006	147037	127188	131686

SQ-3.1 Serum

[Cage B] μM \ [NT] μM	0	4	8	16	32	64	128
**1**	9130	9620	10982	8941	8734	8830	7864
**2**	10610	10350	10298	10180	9980	9820	9278
**4**	12814	12322	14125	12281	11848	12584	11149
**8**	16949	16522	16688	16234	16497	16150	15040
**16**	25738	24494	25138	23834	24004	24401	21618
**32**	39990	40234	40925	41124	41385	41654	36013
**64**	77682	74293	74020	74065	70784	69061	65736
**128**	129706	137597	128641	129550	126126	129024	114581

SQ-3.2 Serum

[Cage B] μM \ [NT] μM	0	4	8	16	32	64	128
**1**	8600	8620	8438	8454	8480	7765	7870
**2**	10226	10470	9998	10013	9677	9277	8724
**4**	11848	12262	12829	11909	11910	11325	10561
**8**	16365	16560	16480	16745	15782	15630	14933
**16**	24781	24897	24621	24037	23681	23897	21916
**32**	40266	40358	41594	41298	39634	39496	35566
**64**	77448	74106	73950	73196	68184	67717	65569
**128**	130385	136516	131173	129932	125674	123529	113990

SQ-3.3 Serum

[Cage B] μM \ [NT] μM	0	4	8	16	32	64	128
**1**	8336	8364	8449	8106	7896	8026	7476
**2**	9753	9952	9933	9821	9457	9289	8757
**4**	11817	12261	12358	12150	11654	11694	10877
**8**	16456	16512	16554	16161	16646	15590	15160
**16**	24849	24973	25062	24606	24428	24454	22336
**32**	42142	42116	41560	41232	40405	39390	36212
**64**	78074	74508	74168	73098	69472	68566	66700
**128**	129618	135708	131308	130601	125829	124252	114354

SQ-4.1 Serum

[NT] µM

[Cage B] µM	0	4	8	16	32	64	128
**1**	9061	8508	9234	8629	7940	8216	7502
**2**	9706	9498	9612	9490	9248	9142	8396
**4**	11326	11861	12261	11340	11125	11453	10424
**8**	16538	16112	15617	15006	14522	14733	13396
**16**	23564	22866	24457	21854	21745	21800	20158
**32**	39582	38157	39325	41737	35793	36009	34436
**64**	71394	74042	70469	69245	63825	69830	61826
**128**	129041	115960	121152	121054	123570	121592	105637

SQ-4.2 Serum

[NT] µM

[Cage B] µM	0	4	8	16	32	64	128
**1**	8422	8429	8785	8840	8242	8097	7561
**2**	9400	9470	9785	9418	9281	8825	8649
**4**	11872	11924	12262	11634	11281	10885	10522
**8**	17060	16702	15658	15576	15302	15714	14082
**16**	23760	23528	25368	23145	22458	21786	20169
**32**	40736	39326	39348	41129	36793	35952	36476
**64**	75002	71789	73562	73617	69614	72708	63981
**128**	128873	133178	129233	129268	127370	124305	108160

SQ-4.3 Serum

[NT] µM

[Cage B] µM	0	4	8	16	32	64	128
**1**	8540	8437	8722	8356	8324	7850	7620
**2**	9473	9265	9481	9320	9160	9125	8906
**4**	11785	12017	11874	11692	11525	11336	10552
**8**	16545	16924	16036	15852	15769	15089	14493
**16**	24568	24074	24717	23504	23254	22774	20920
**32**	41970	40762	41197	41314	37344	37570	34898
**64**	74885	73554	73781	74953	69900	69881	65806
**128**	131248	134584	129424	129821	127437	122180	112576

Analysis were performed using Origin 2018 program. T-student test, for intercept and slope, were calculated at a significance level of 5% (α=0.05). The relationship between the different assays was carried out by calculating the correlation coefficient (Pearson's r). It showed a high coefficient of determination in all cases and the T-values determined that both, the slopes and intercepts, are different from zero. The Stern-Volmer plot of F_0/F versus NT concentration allows us to obtain an association constant in 2:6:2 DMSO/Buffer PBS 7,4/serum from the slope of the linear fits.

Regressions were made with the average of all the dates from the sera at same concentration of NT and cage **B**. Error bars correspond to the standard deviation.

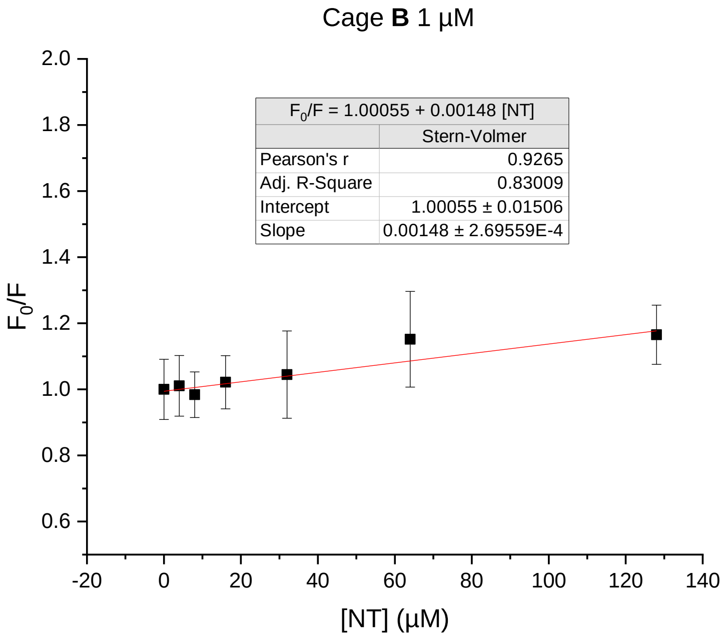

Figure S16. Regression analysis of 1 µM of cage **B** in presence of increasing amounts of NT

	Value	Standard Error	t-Value	Prob>|t|
**Intercept**	1.00055	0.01506	66.45207	1.46119E-8
**Slope**	0.00148	2.69559E-4	5.50572	0.0027

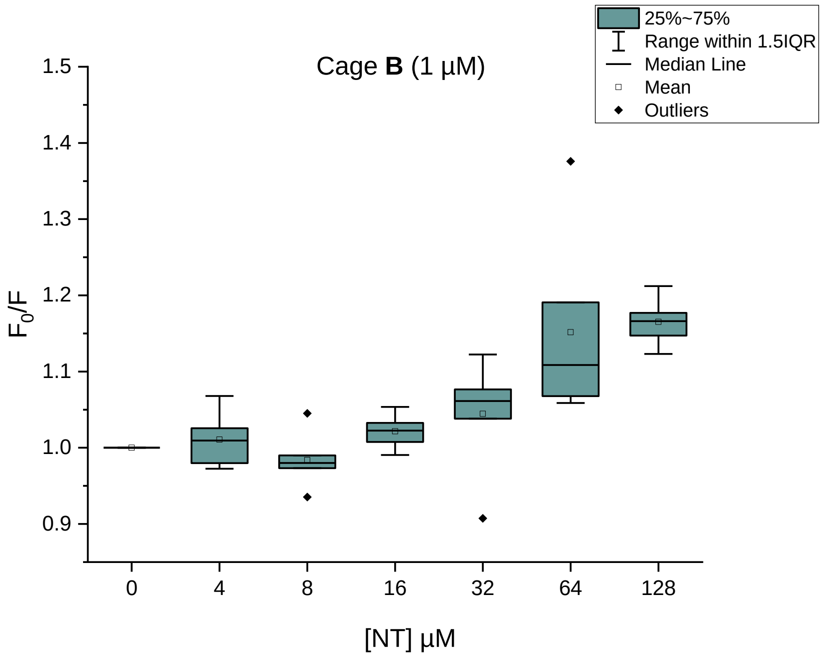

Figure S17. Box graph of 1 µM of cage **B** in presence of increasing amounts of NT

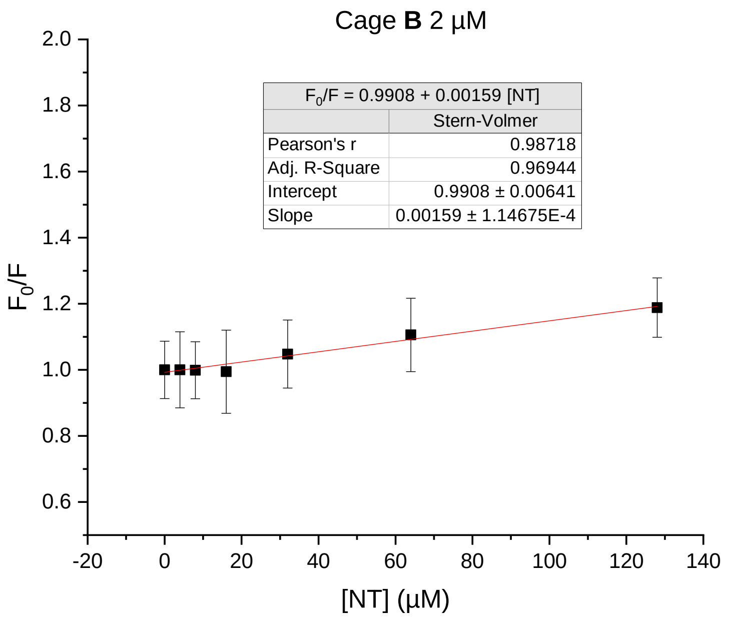

Figure S18. Regression analysis of 2 µM of cage **B** in presence of increasing amounts of NT

	Value	Standard Error	t-Value	Prob>|t|
**Intercept**	0.9908	0.00641	154.68148	2.14249E-10
**Slope**	0.00159	1.14675E-4	13.83127	3.54791E-5

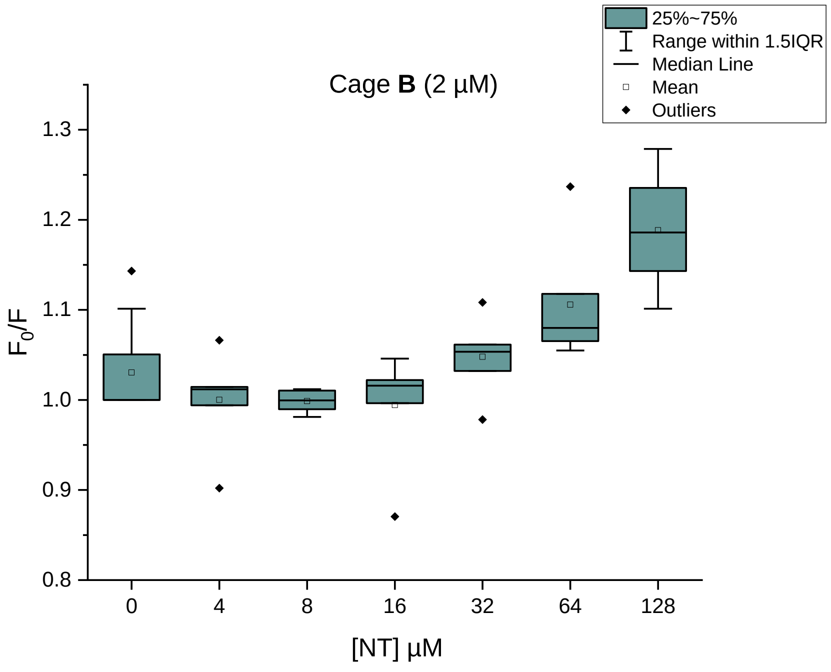

Figure S19. Box graph of 2 µM of cage **B** in presence of increasing amounts of NT

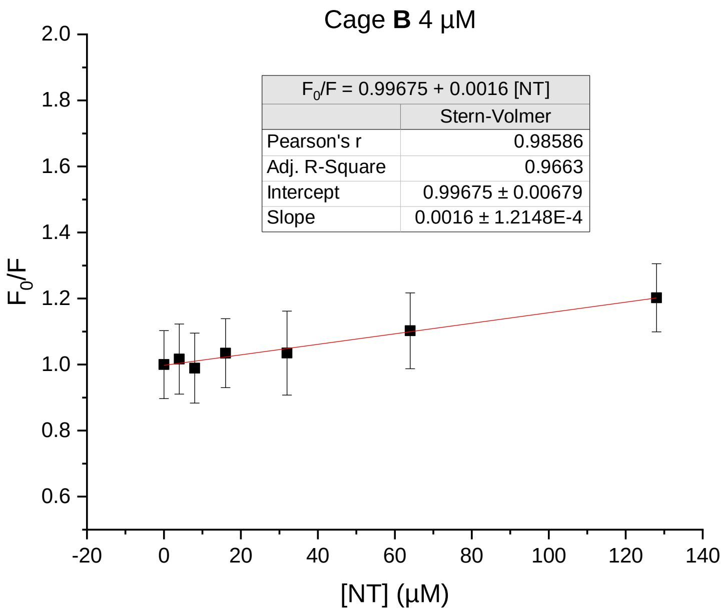

Figure S20. Regression analysis of 4 µM of cage **B** in presence of increasing amounts of NT

	Value	Standard Error	t-Value	Prob>|t|
**Intercept**	0.99675	0.00679	146.89455	2.7737E-10
**Slope**	0.0016	1.2148E-4	13.15428	4.53362E-5

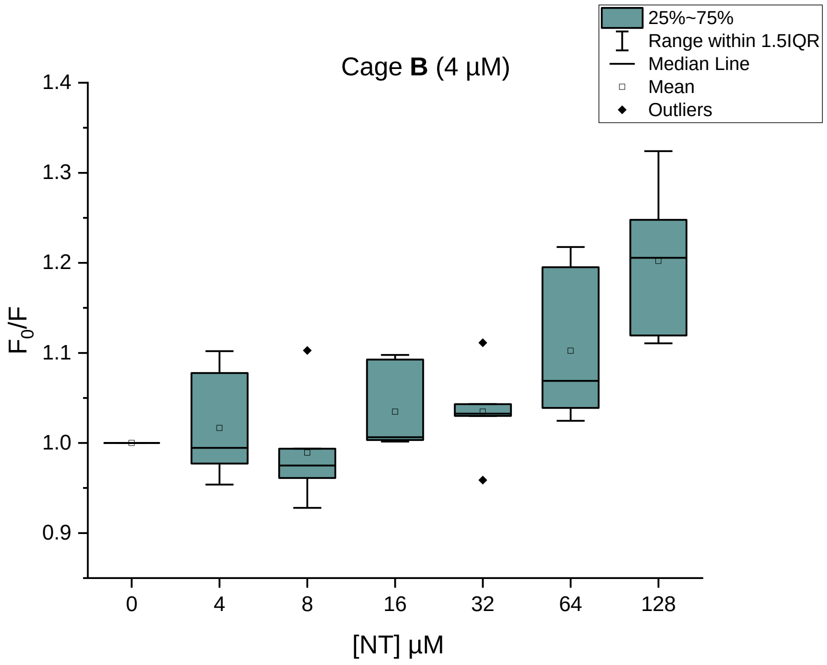

Figure S21. Box graph of 4 µM of cage **B** in presence of increasing amounts of NT

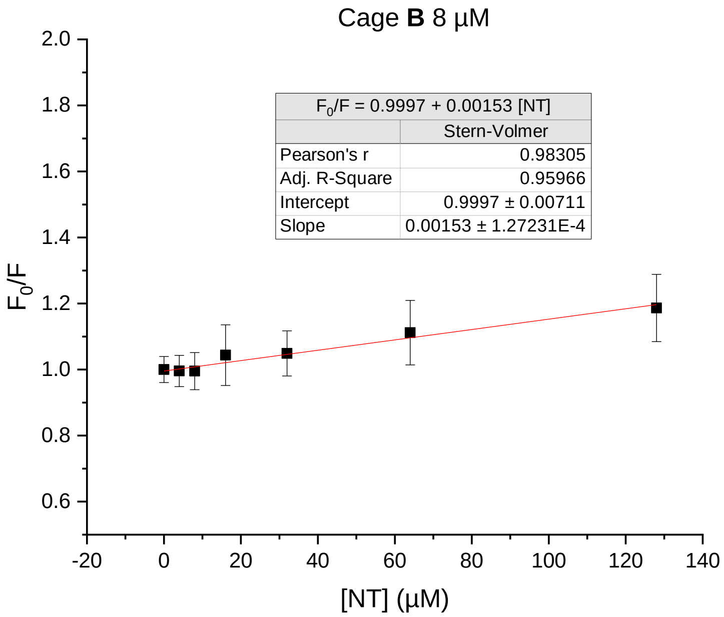

Figure S22. Regression analysis of 8 µM of cage **B** in presence of increasing amounts of NT

	Value	Standard Error	t-Value	Prob>|t|
**Intercept**	0.9997	0.00711	140.66899	3.44411E-10
**Slope**	0.00153	1.27231E-4	11.98862	7.12222E-5

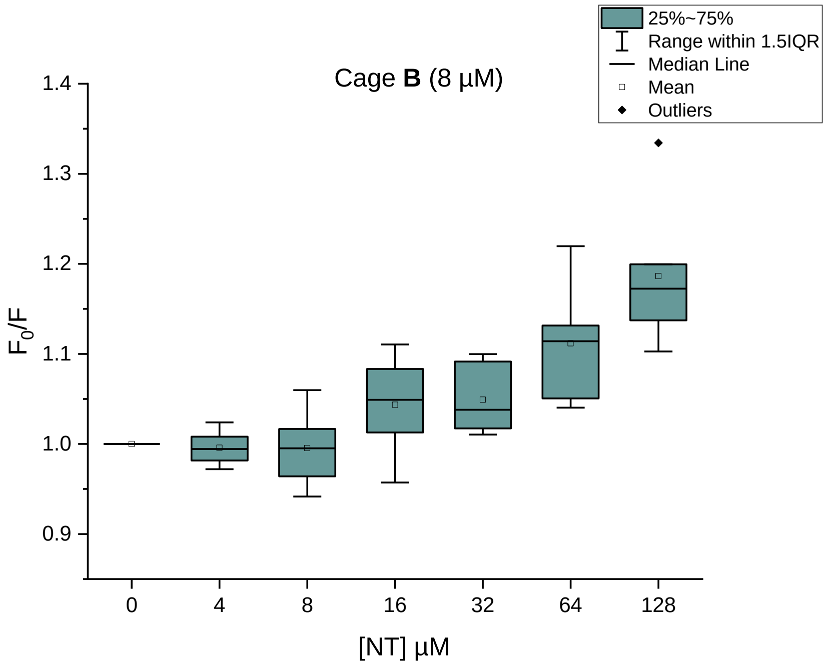

Figure S23. Box graph of 8 µM of cage **B** in presence of increasing amounts of NT

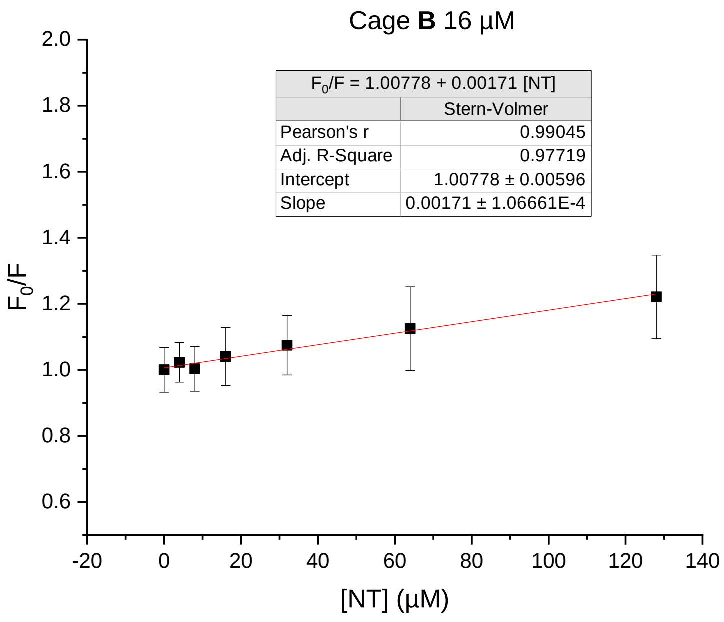

Figure S24. Regression analysis of 16 µM of cage **B** in presence of increasing amounts of NT

	Value	Standard Error	t-Value	Prob>|t|
**Intercept**	1.00778	0.00596	169.15441	1.37001E-10
**Slope**	0.00171	1.06661E-4	16.06518	1.70221E-5

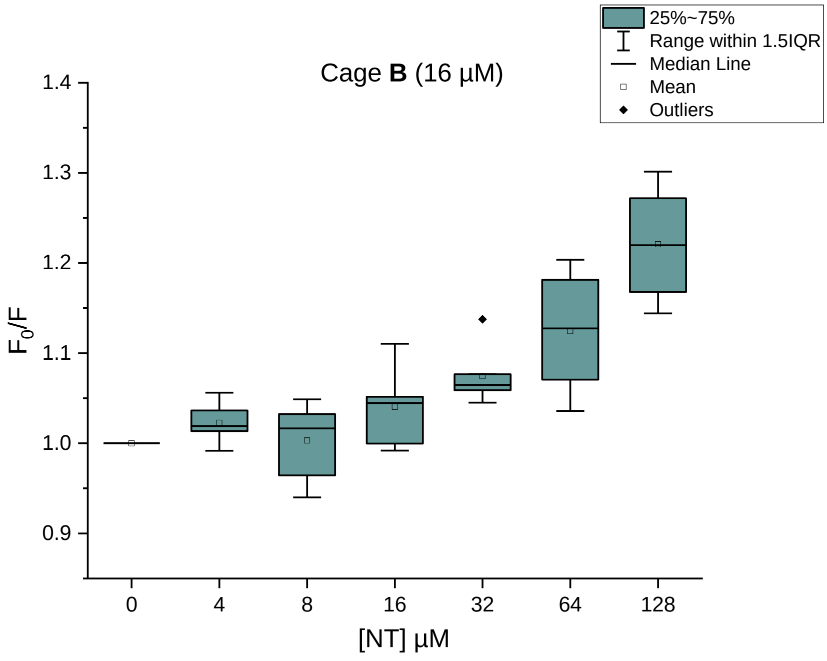

Figure S25. Box graph of 16 µM of cage **B** in presence of increasing amounts of NT

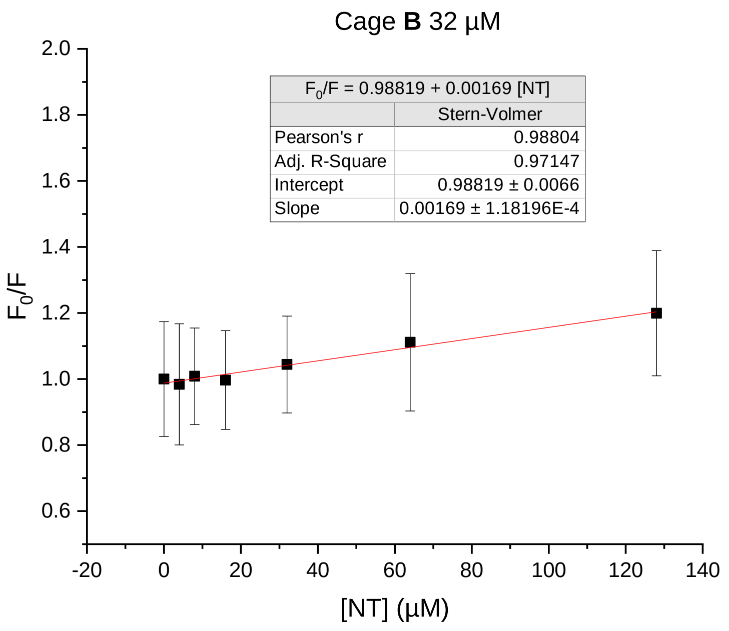

Figure S26. Regression analysis of 32 µM of cage **B** in presence of increasing amounts of NT

	Value	Standard Error	t-Value	Prob>|t|
**Intercept**	0.98819	0.0066	149.67877	2.5252E-10
**Slope**	0.00169	1.18196E-4	14.32887	2.98431E-5

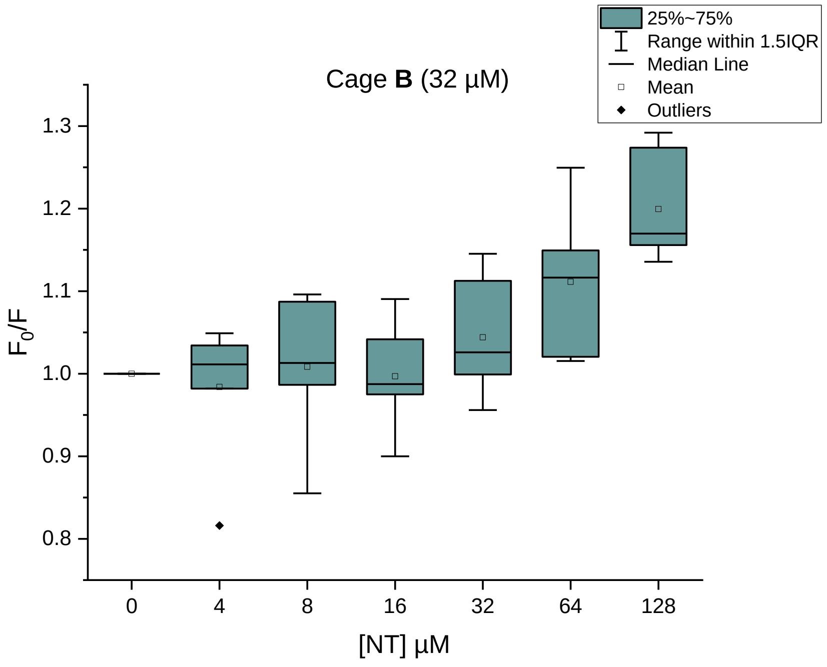

Figure S27. Box graph of 32 µM of cage **B** in presence of increasing amounts of NT

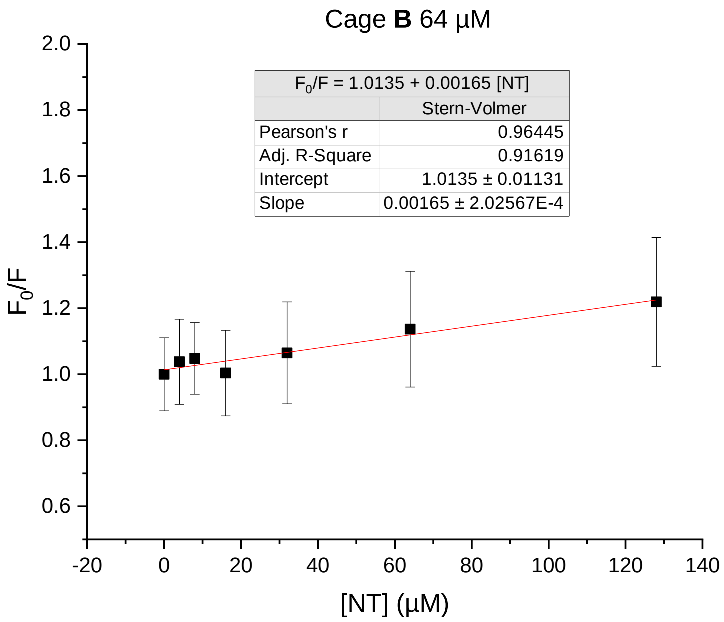

Figure S28. Regression analysis of 64 µM of cage **B** in presence of increasing amounts of NT

	Value	Standard Error	t-Value	Prob>|t|
**Intercept**	1.0135	0.01131	89.57293	3.28731E-9
**Slope**	0.00165	2.02567E-4	8.16044	4.49038E-4

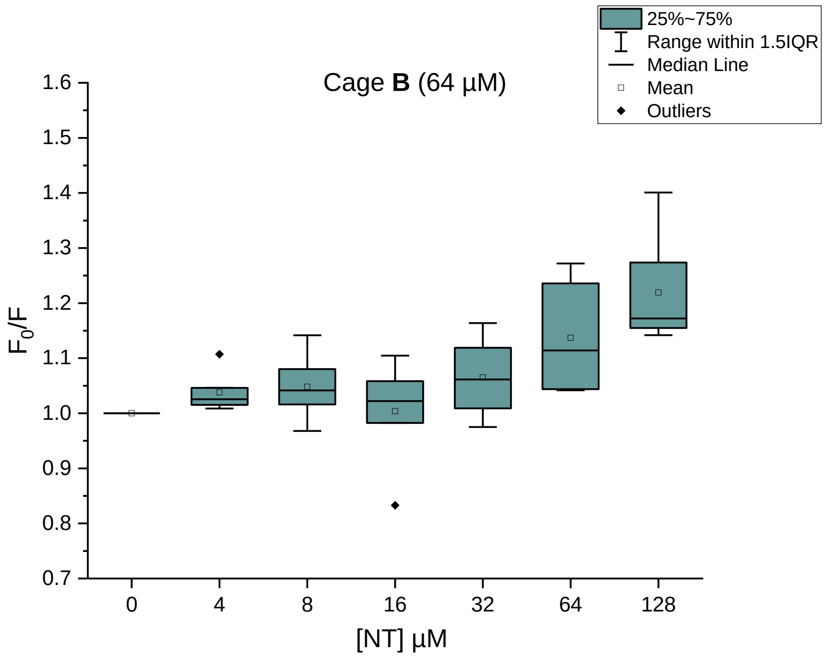

Figure S29. Box graph of 64 µM of cage **B** in presence of increasing amounts of NT

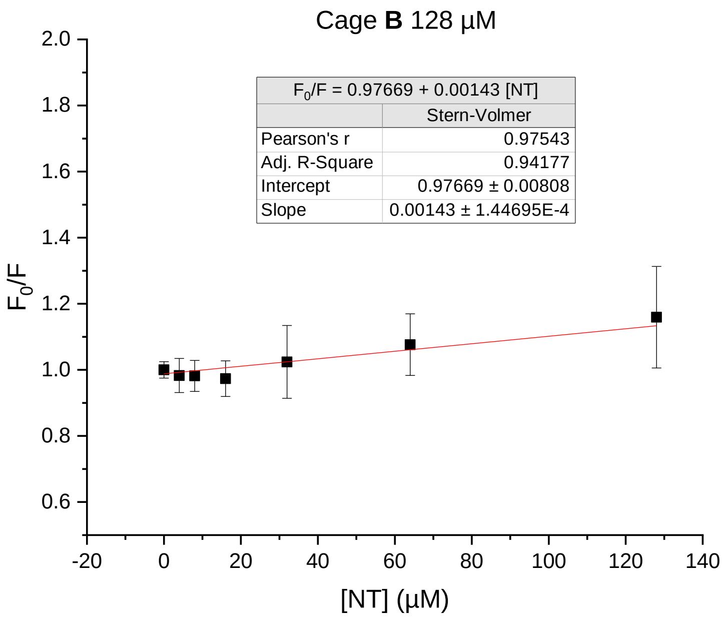

Figure S30. Regression analysis of 128 µM of cage **B** in presence of increasing amounts of NT

	Value	Standard Error	t-Value	Prob>|t|
**Intercept**	0.97669	0.00808	120.84461	7.35951E-10
**Slope**	0.00143	1.44695E-4	9.90132	1.7927E-4

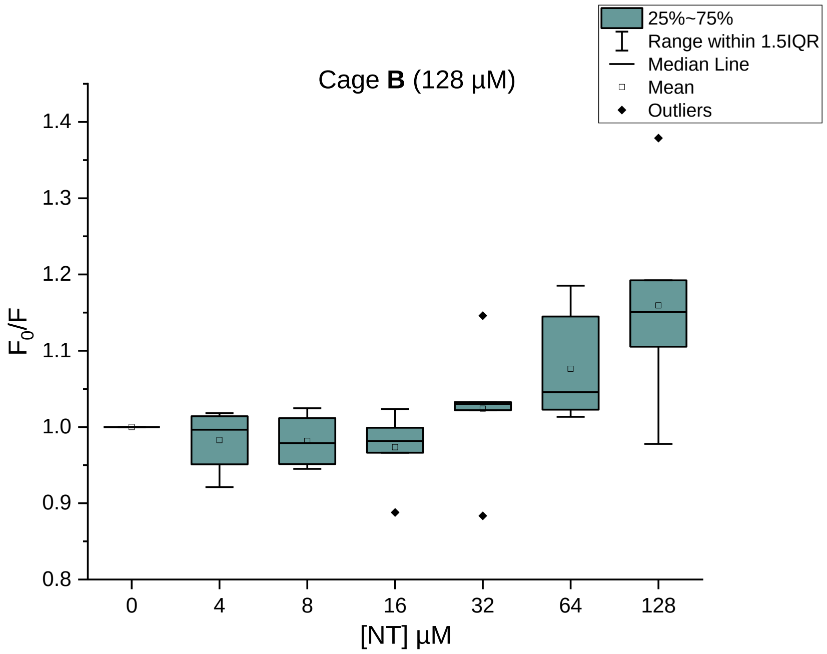

Figure S31. Box graph of 128 µM of cage **B** in presence of increasing amounts of NT

7.1 Determination of limit of detection in human serum

Limit of detection (LoD) was calculated based on a method reported in previous literature.[8] It is defined as the minimum analyte concentration that provides a reliable signal for the applied analytical method. It was calculated for the experiments carried out in diluted human serum, particularly when cage **B** concentration was 16 µM. Changes in F_0/F at 16 µM of cage **B** were plotted versus NT as it can be seen in Figure 7 in the manuscript.

Limit of detection was calculated with the following equation:

$$\mathrm{LoD} = \frac{3.3\sigma}{b}$$

Where σ is the standard error of the linear regression (0.01204) and b is the slope ($1.71 \cdot 10^{-3}$). The detection limit is LoD = $2,3 \times 10^{-5}$ mol L^{-1}.

The LoD was also calculated from the mean and standard deviation of the replicate blank readings, $F_{blank} \pm s_{blank}$, which were obtained from the first column of the tables on pages S25-S30. Then, following the well-known equation[9]:

$F_{LOD} = F_{blank} - 3.3s_{blank}$

We obtained the minimum detectable signal (F_{LOD}). The formula is a subtraction because of the quenching of the fluorescence. Then we converted such value into the concentration limit of detection using the appropriate regression line.

Depending on the data used (different sera, different concentrations of cage…) we obtained a LoD varying from 18.75 to 40.8 µM which are in the same range than that obtained by the abovementioned methodology.

8. Binding of cage B with 1,3,5-trinitrotoluene (TNT) in Ethyl Acetate.

Supramolecular binding of cage **B** with a nitroaromatic guest in a pure organic solvent was also studied, in order to evaluate the relevance of the hydrophobic effect in the association. We chose TNT as guest. Even when it is not 3-nitrotyrosine (NT), it is relatively similar and it could serve as a model to study the binding in organic solvent. As it can be seen in Figures S35, the association constant (equivalent to K_{SV}) of cage **B** with TNT is K_a = 405 M^{-1}, almost two orders of magnitude lower than the constant of cage **B** with NT in THF/water (2:8).

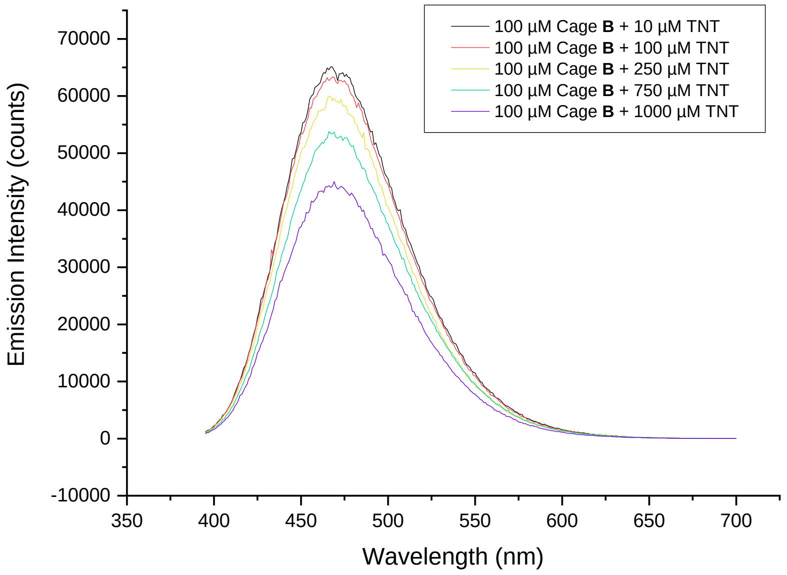

Figure S32. Fluorescence quenching of cage **B** (100 µM) at different TNT concentrations in ethyl acetate.

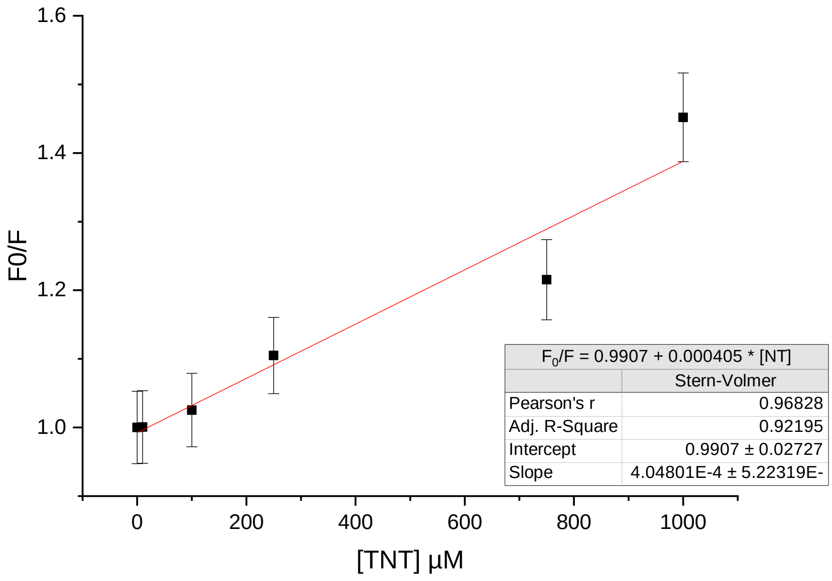

Figure S33. Stern-Volmer plot of cage **B** fluorescence quenching with TNT in ethyl acetate.

9. Selectivity measurements

Different common bioanalytes were tested with cage B to check if they were able to quench the fluorescence of the sensor. In all cases a concentration of 25 µM was used. No quenching was observed for any of the bioanalytes.

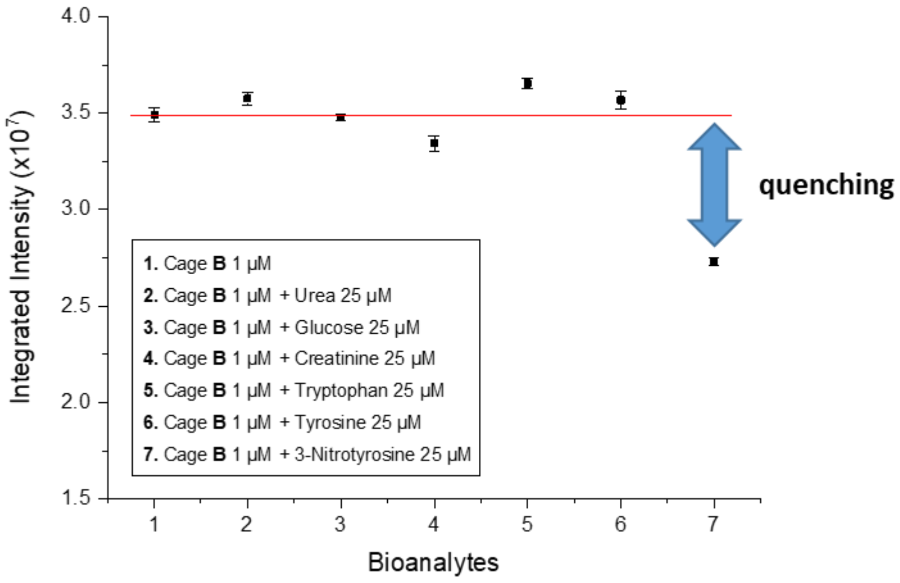

Figure S34. Comparative quenching ability of several bioanalytes.

Finally, to validate the method, a solution of NT in serum with a known final concentration after dilution (64 µM) was made. That sample was analyzed with our technique in a FLUOStar Omega microplate reader (BMG Labtech) with an excitation filter with wavelength of 380 nm, and an emission filter with wavelength of 470 nm:

A concentration of 16 µM of cage **B** was employed. We took F_0 as the average value of all the F_0 values obtained in all the previous experiments (18 measurements), F_0 = 25011.8 ± 846. Then the fluorescence of the mixture was measured, and the value obtained for F_0/F (1.1149) was inserted in the calibration curve for 16 µM of cage **B** (F_0/F = 1.00778 + 0.00171[NT]), yielding a concentration of nitrotyrosine [NT] = 63 µM

Fortunately, comparing with a previously reported method gave similar results.[10]

10. NMR spectra

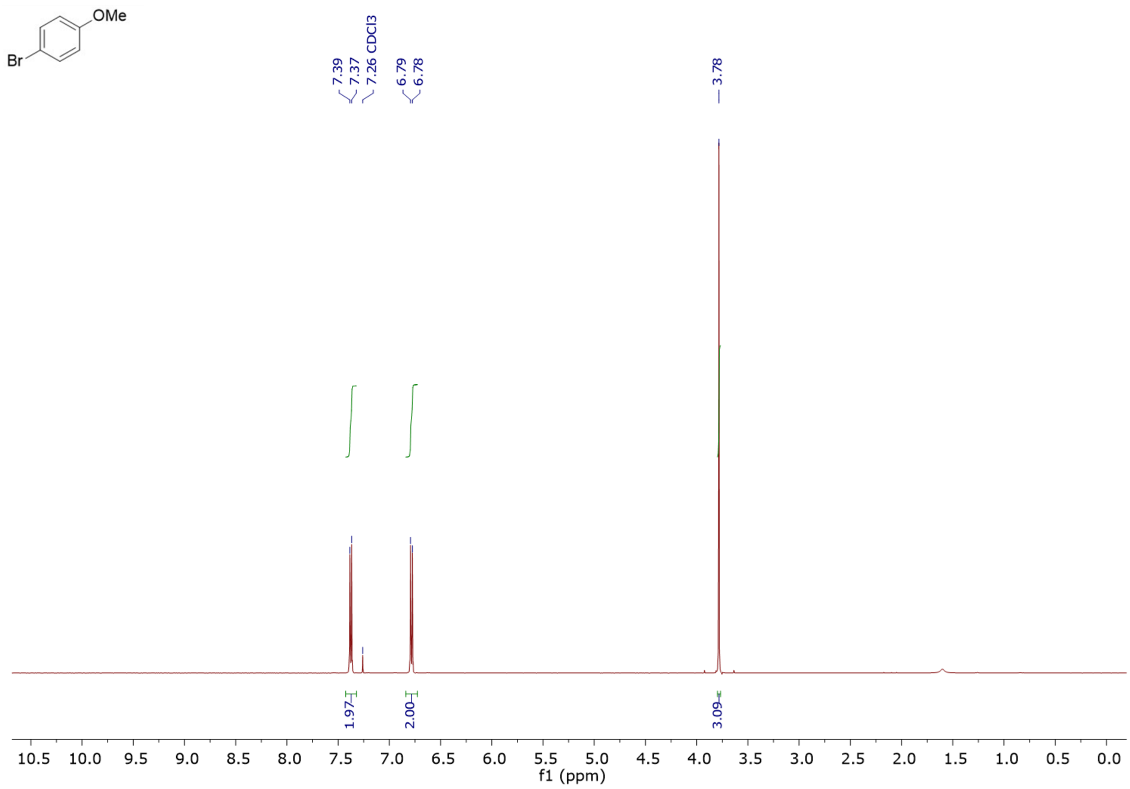

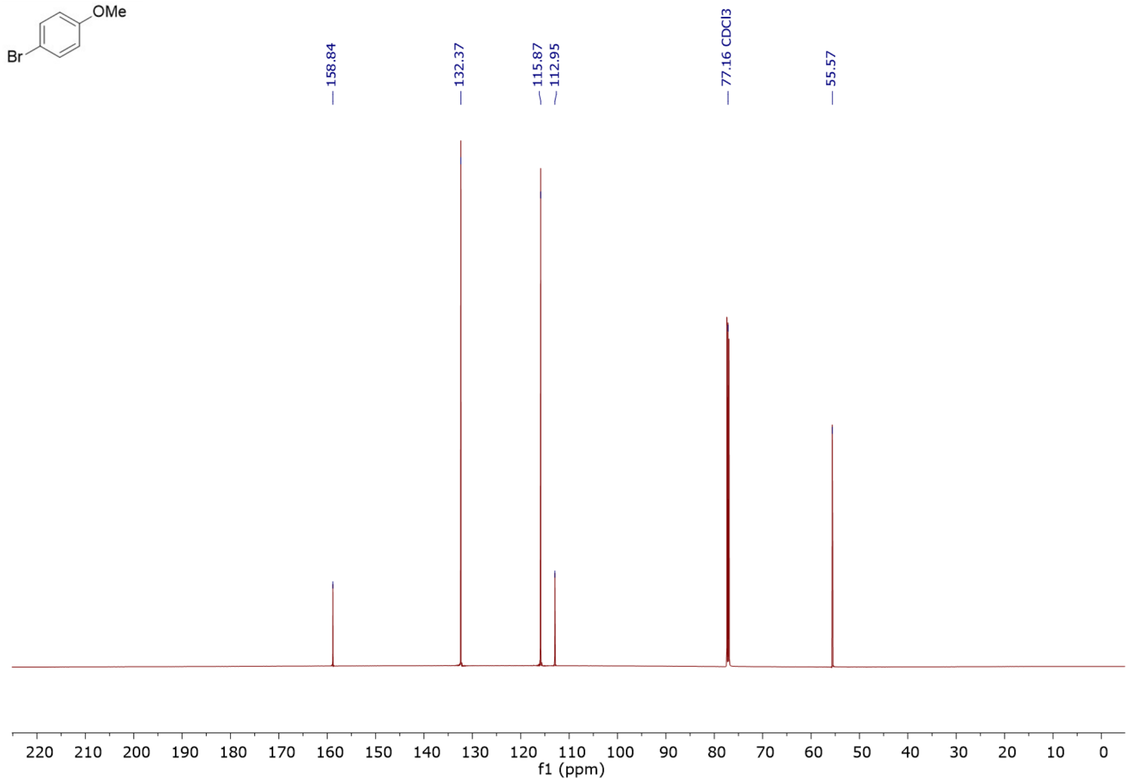
OMe
Br
158.84
132.37
115.87
112.95
77.16 CDCl3
55.57
220 210 200 190 180 170 160 150 140 130 120 110 100 90 80 70 60 50 40 30 20 10 0
f1 (ppm)

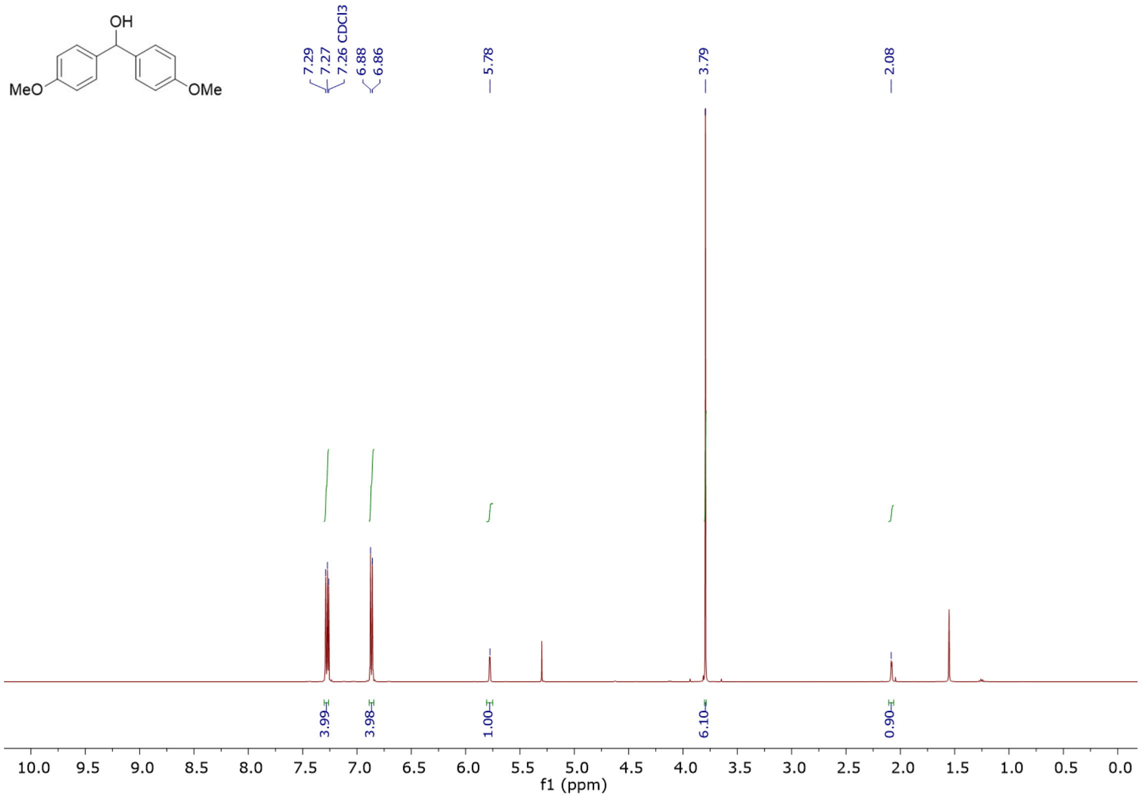
OH
MeO
OMe
7.29
7.27
7.26 CDCl3
6.88
6.86
5.78
3.79
2.08
3.99
3.98
1.00
6.10
0.90
10.0 9.5 9.0 8.5 8.0 7.5 7.0 6.5 6.0 5.5 5.0 4.5 4.0 3.5 3.0 2.5 2.0 1.5 1.0 0.5 0.0
f1 (ppm)

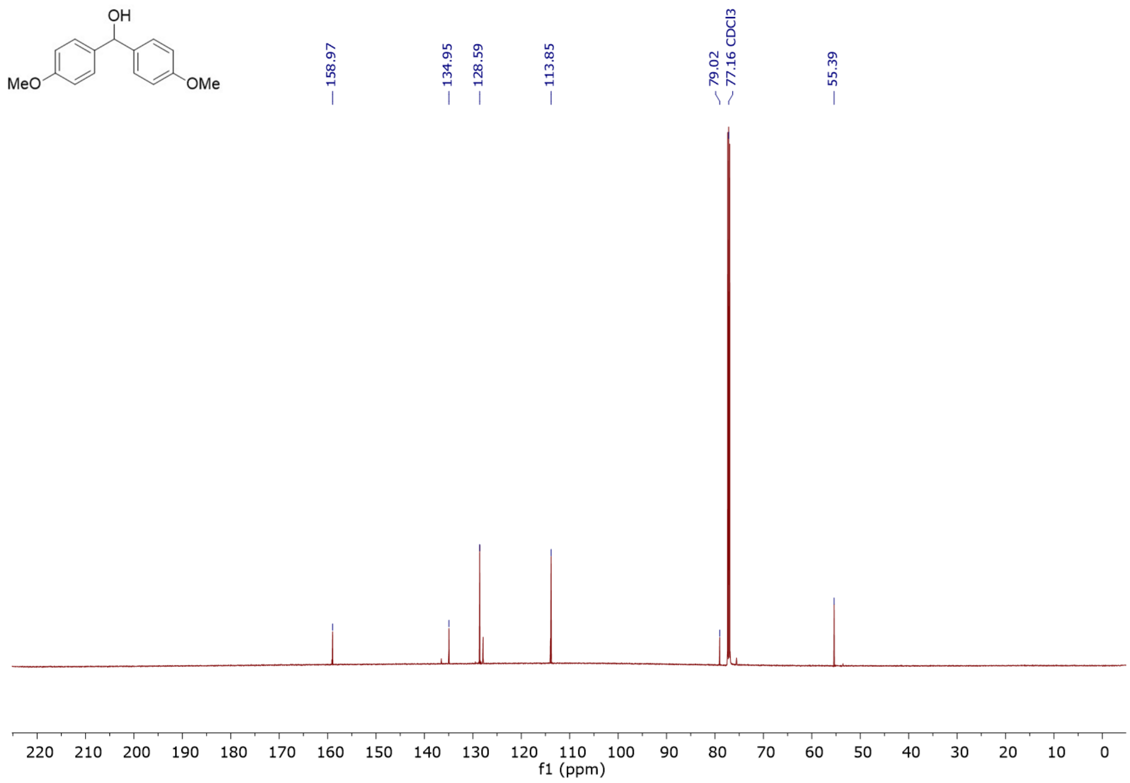
OH
MeO
OMe
158.97
134.95
128.59
113.85
79.02
77.16 CDCl3
55.39
220 210 200 190 180 170 160 150 140 130 120 110 100 90 80 70 60 50 40 30 20 10 0
f1 (ppm)

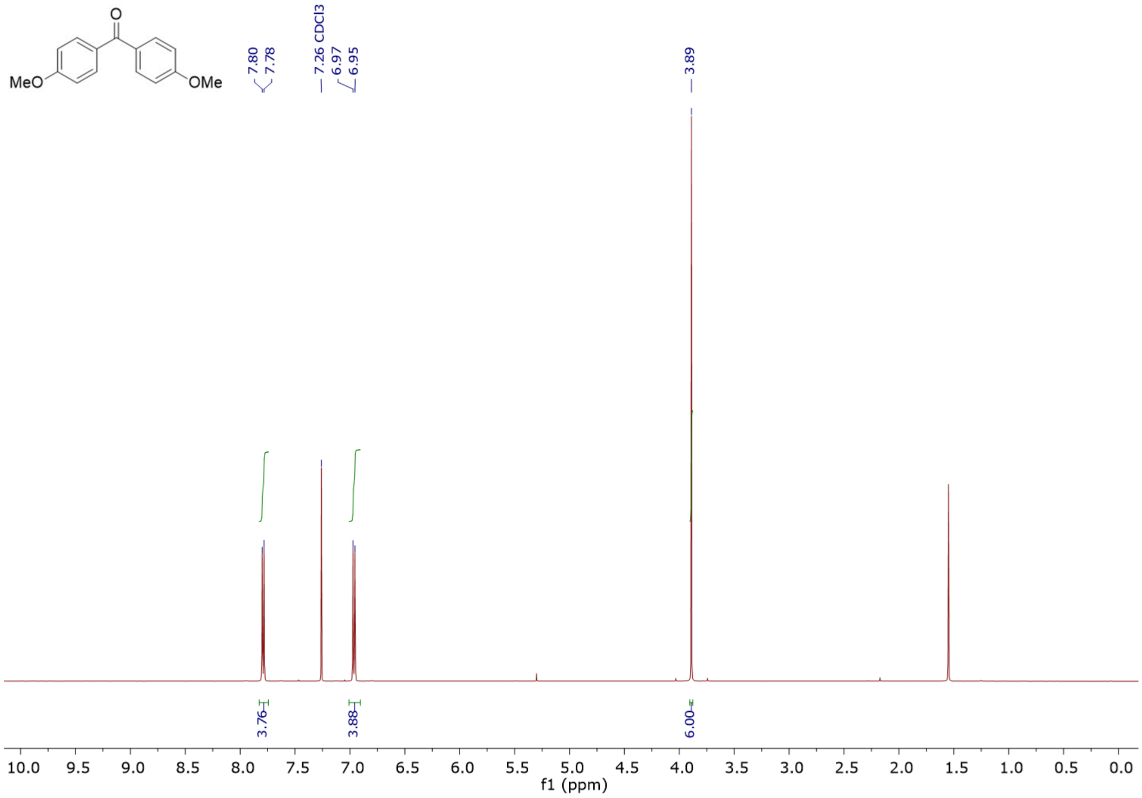
O
MeO
OMe
7.80
7.78
7.26 CDCl3
6.97
6.95
3.89
3.76
3.88
6.00
10.0 9.5 9.0 8.5 8.0 7.5 7.0 6.5 6.0 5.5 5.0 4.5 4.0 3.5 3.0 2.5 2.0 1.5 1.0 0.5 0.0
f1 (ppm)

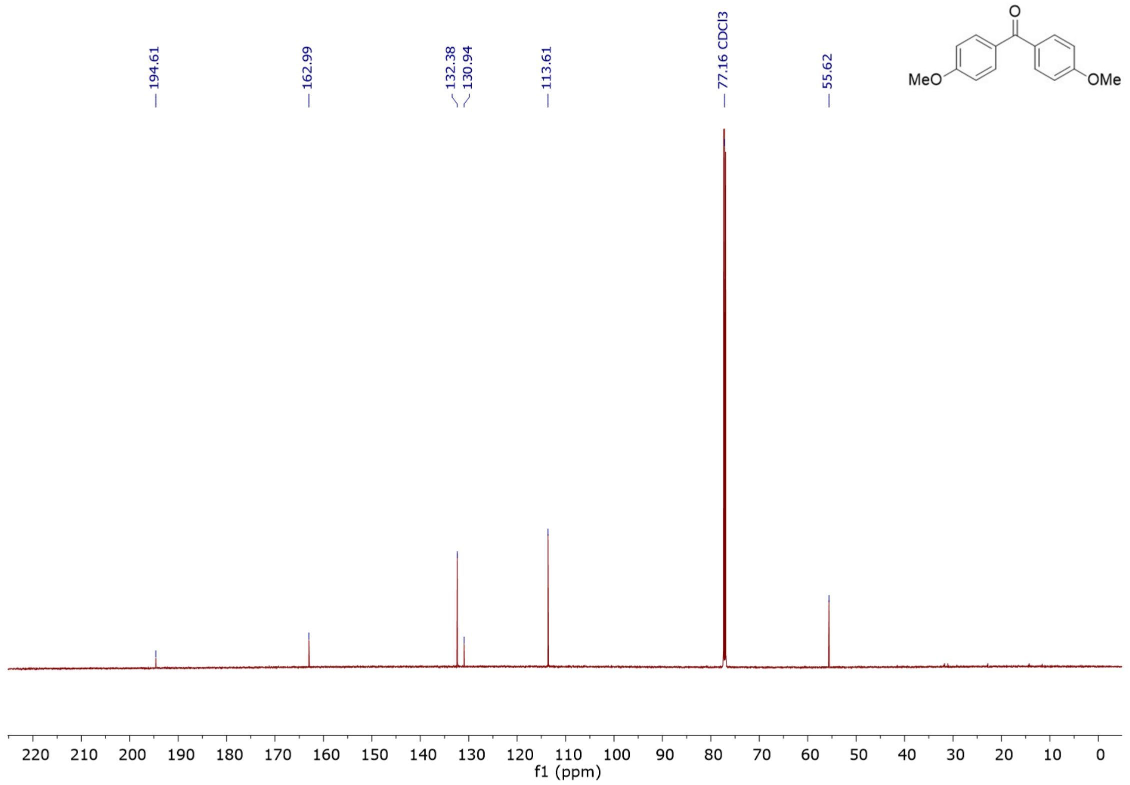
194.61
162.99
132.38
130.94
113.61
77.16 CDCl3
55.62
MeO
OMe
O
220 210 200 190 180 170 160 150 140 130 120 110 100 90 80 70 60 50 40 30 20 10 0
f1 (ppm)

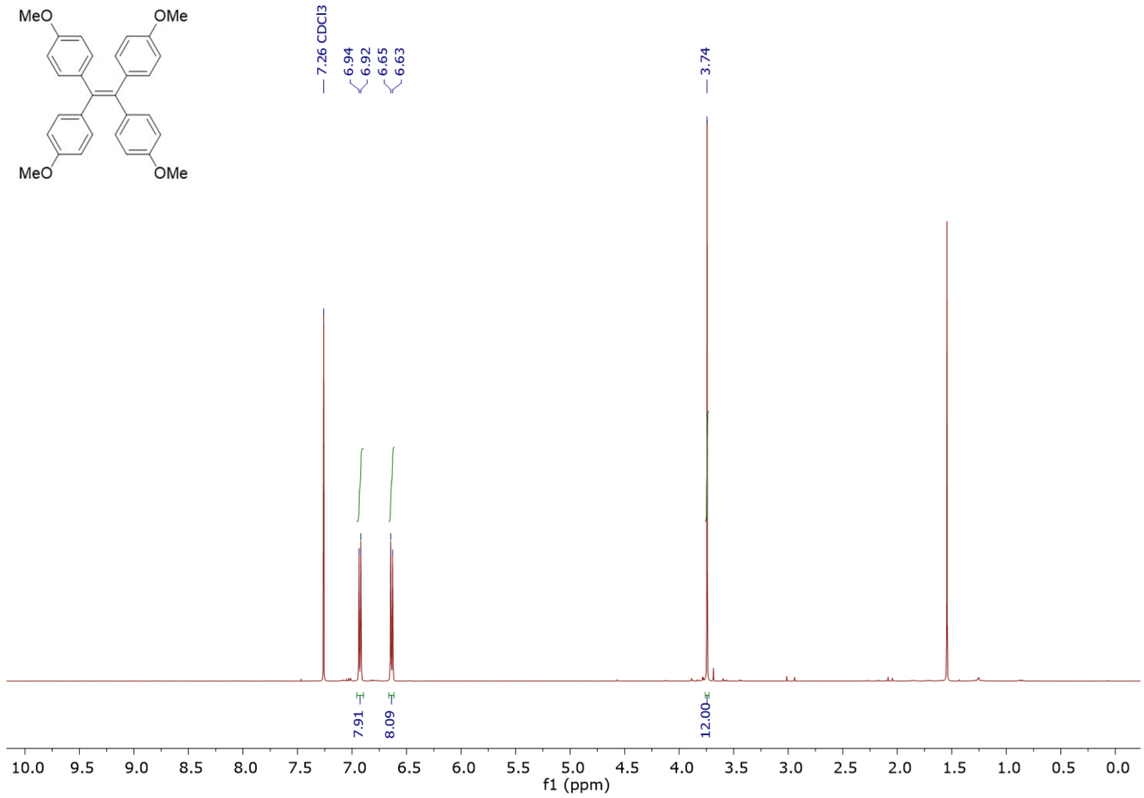
MeO
OMe
MeO
OMe
7.26 CDCl3
6.94
6.92
6.65
6.63
3.74
7.91
8.09
12.00
10.0 9.5 9.0 8.5 8.0 7.5 7.0 6.5 6.0 5.5 5.0 4.5 4.0 3.5 3.0 2.5 2.0 1.5 1.0 0.5 0.0
f1 (ppm)

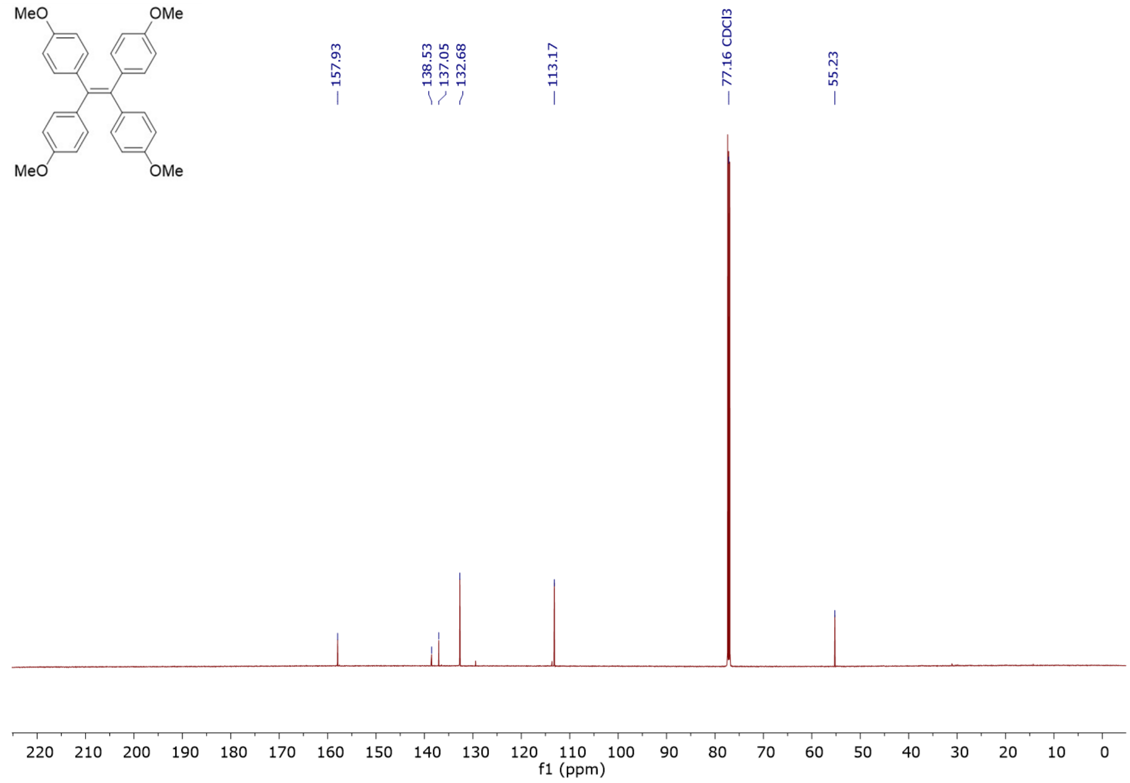
MeO
OMe
MeO
OMe
157.93
138.53
137.05
132.68
113.17
77.16 CDCl3
55.23
220 210 200 190 180 170 160 150 140 130 120 110 100 90 80 70 60 50 40 30 20 10 0
f1 (ppm)

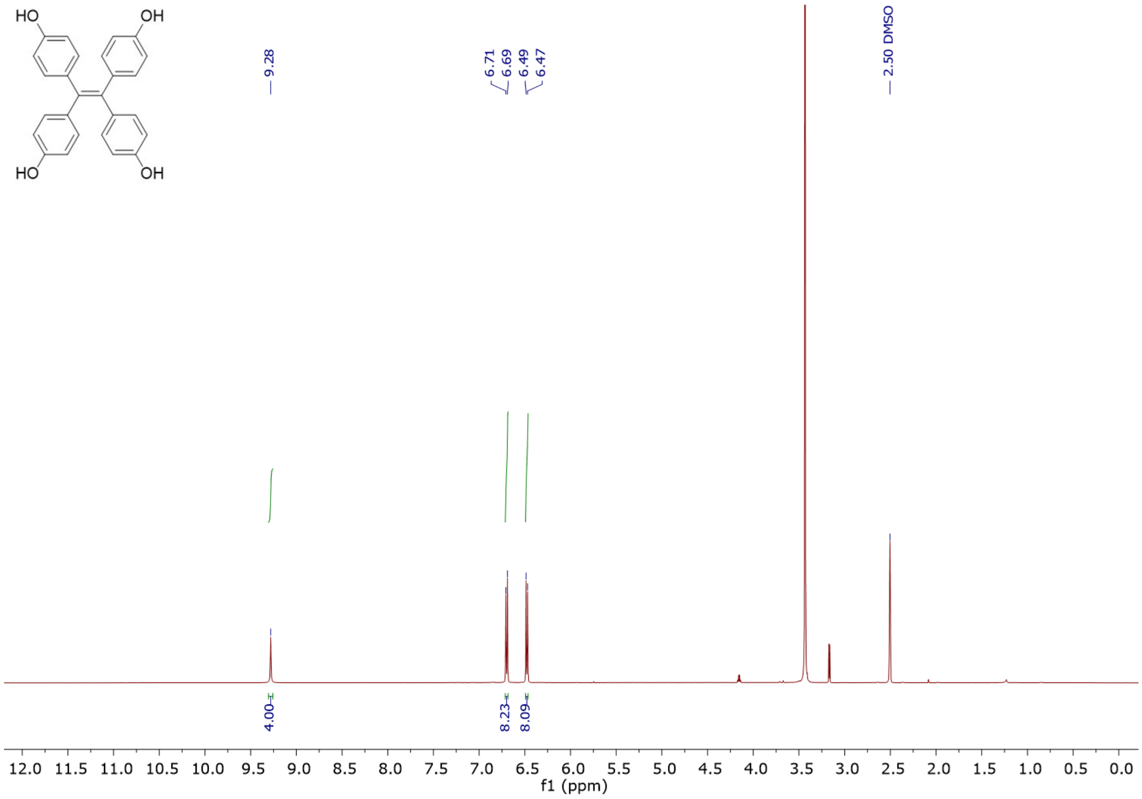
HO
OH
HO
OH
9.28
6.71
6.69
6.49
6.47
2.50 DMSO
4.00
8.23
8.09
12.0 11.5 11.0 10.5 10.0 9.5 9.0 8.5 8.0 7.5 7.0 6.5 6.0 5.5 5.0 4.5 4.0 3.5 3.0 2.5 2.0 1.5 1.0 0.5 0.0
f1 (ppm)

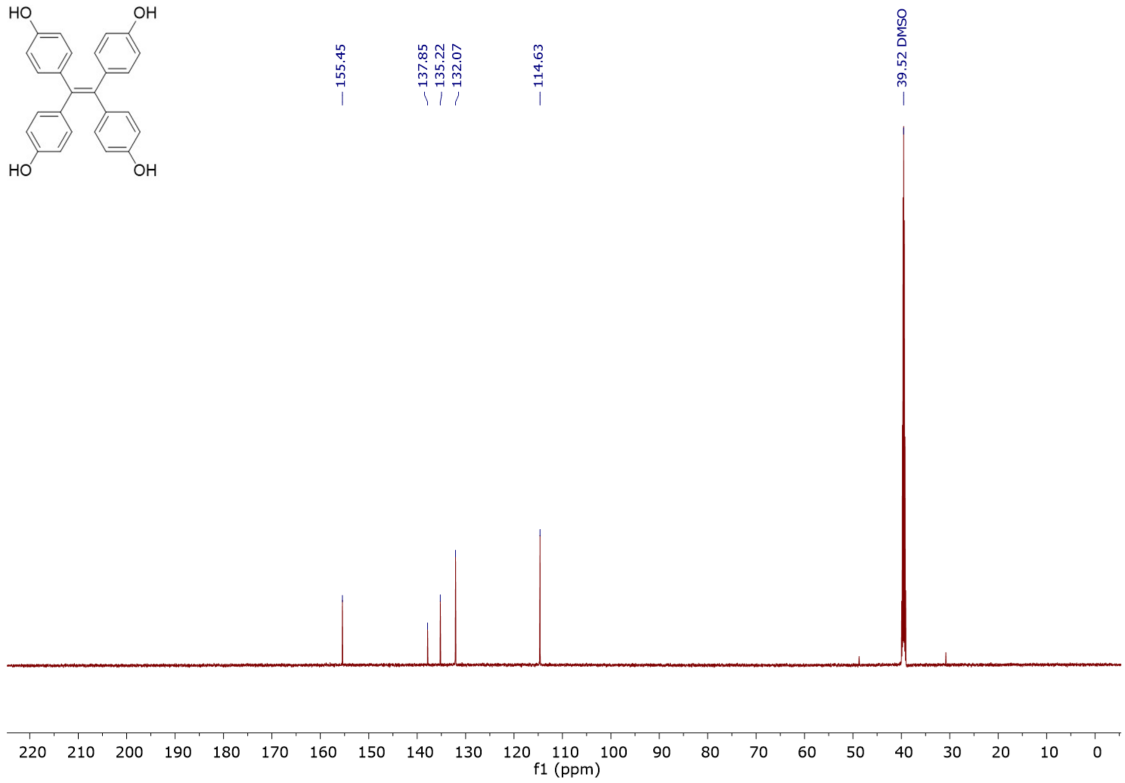
HO
OH
HO
OH
155.45
137.85
135.22
132.07
114.63
39.52 DMSO
220 210 200 190 180 170 160 150 140 130 120 110 100 90 80 70 60 50 40 30 20 10 0
f1 (ppm)

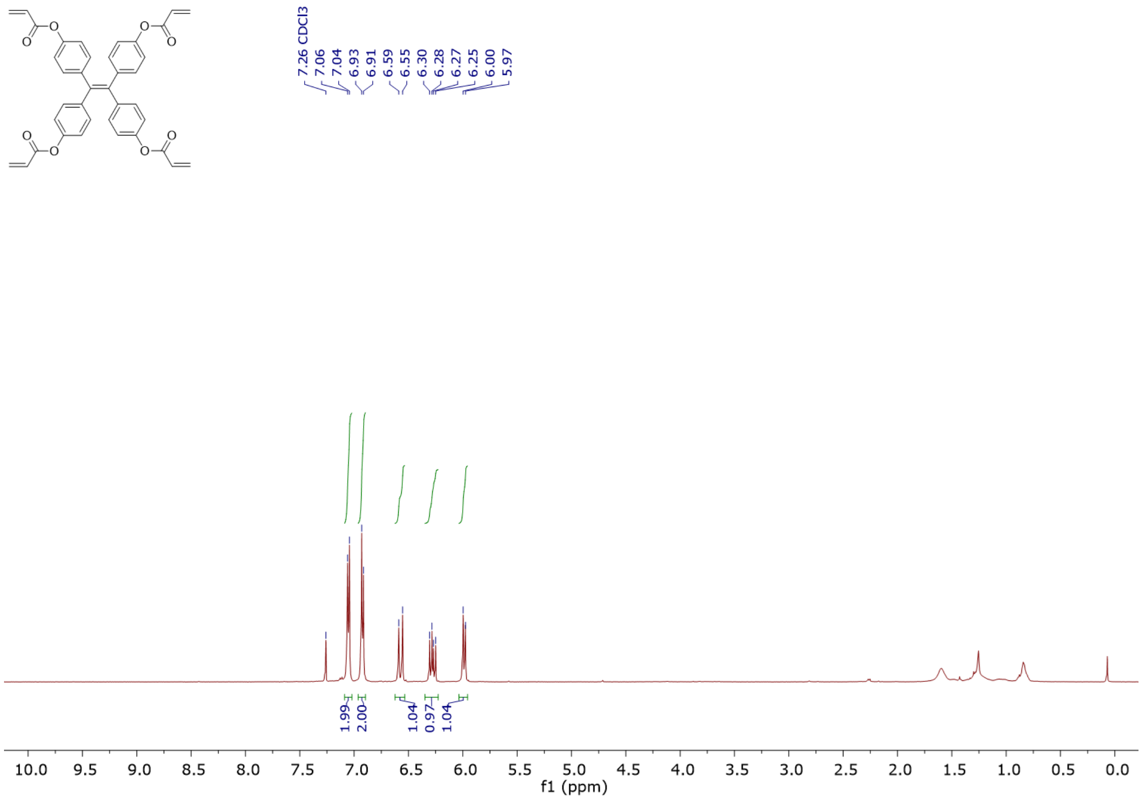
7.26 CDCl3
7.06
7.04
6.93
6.91
6.59
6.55
6.30
6.28
6.27
6.25
6.00
5.97
1.99
2.00
1.04
0.97
1.04
10.0 9.5 9.0 8.5 8.0 7.5 7.0 6.5 6.0 5.5 5.0 4.5 4.0 3.5 3.0 2.5 2.0 1.5 1.0 0.5 0.0
f1 (ppm)

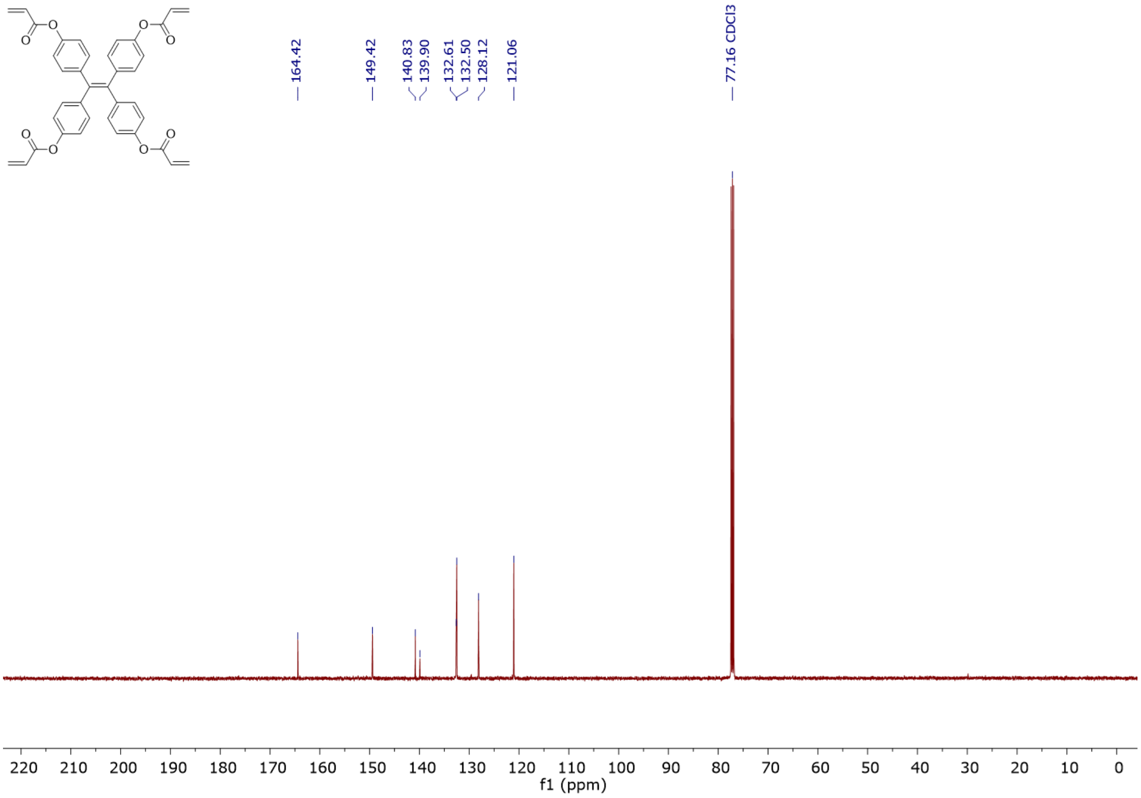
164.42
149.42
140.83
139.90
132.61
132.50
128.12
121.06
77.16 CDCl3
220 210 200 190 180 170 160 150 140 130 120 110 100 90 80 70 60 50 40 30 20 10 0
f1 (ppm)

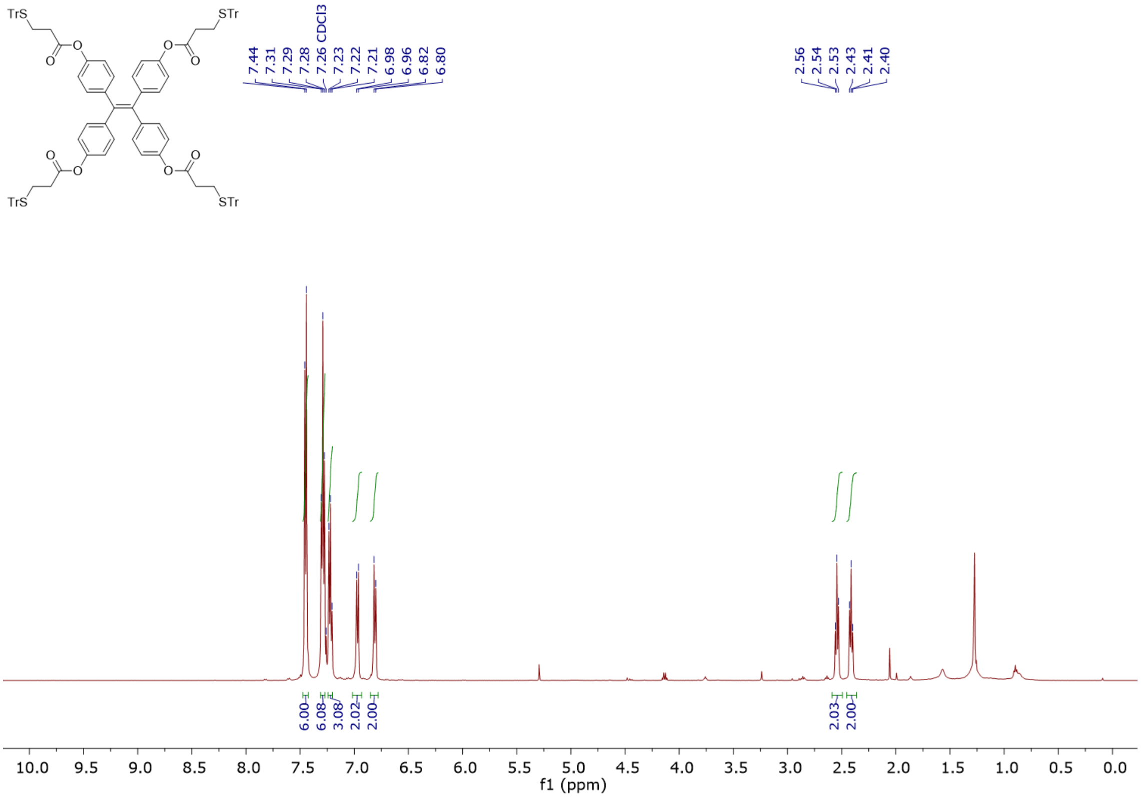
TrS
STr
TrS
STr
7.44
7.31
7.29
7.28
7.26 CDCl3
7.23
7.22
7.21
6.98
6.96
6.82
6.80
2.56
2.54
2.53
2.43
2.41
2.40
6.00
6.08
3.08
2.02
2.00
2.03
2.00
10.0 9.5 9.0 8.5 8.0 7.5 7.0 6.5 6.0 5.5 5.0 4.5 4.0 3.5 3.0 2.5 2.0 1.5 1.0 0.5 0.0
f1 (ppm)

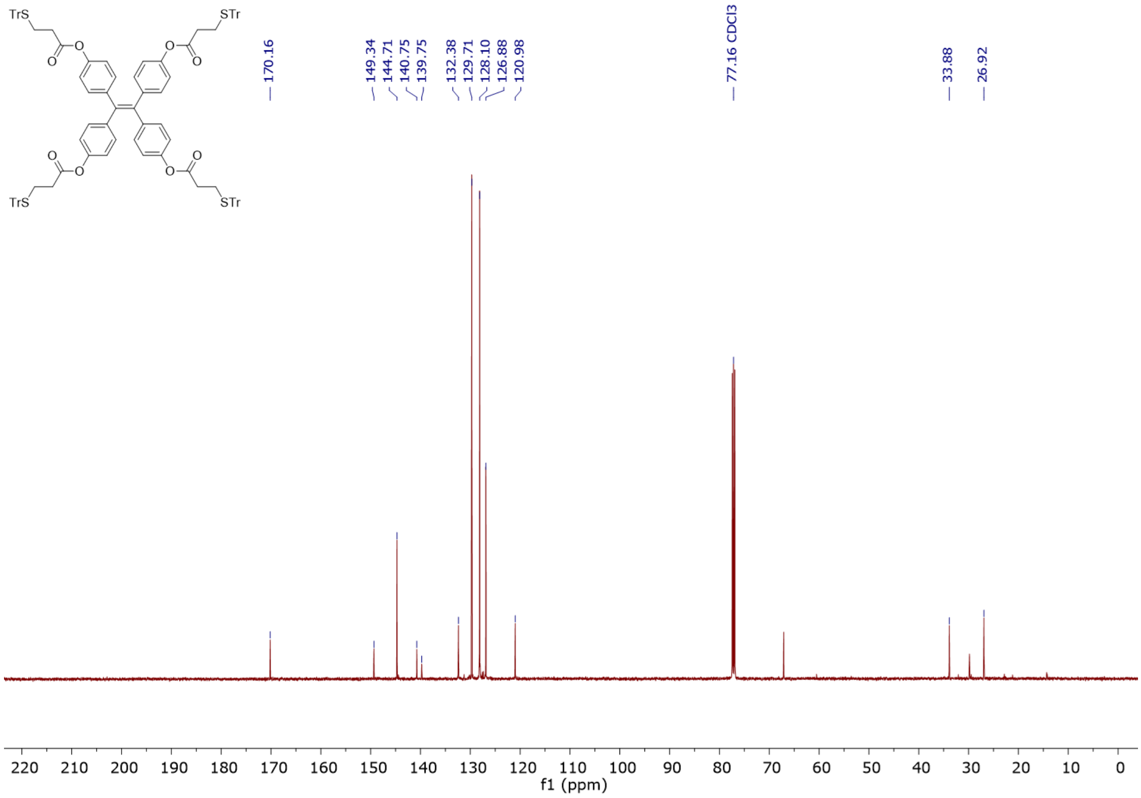
TrS
STr
O
TrS
STr
170.16
149.34
144.71
140.75
139.75
132.38
129.71
128.10
126.88
120.98
77.16 CDCl3
33.88
26.92
220 210 200 190 180 170 160 150 140 130 120 110 100 90 80 70 60 50 40 30 20 10 0
f1 (ppm)

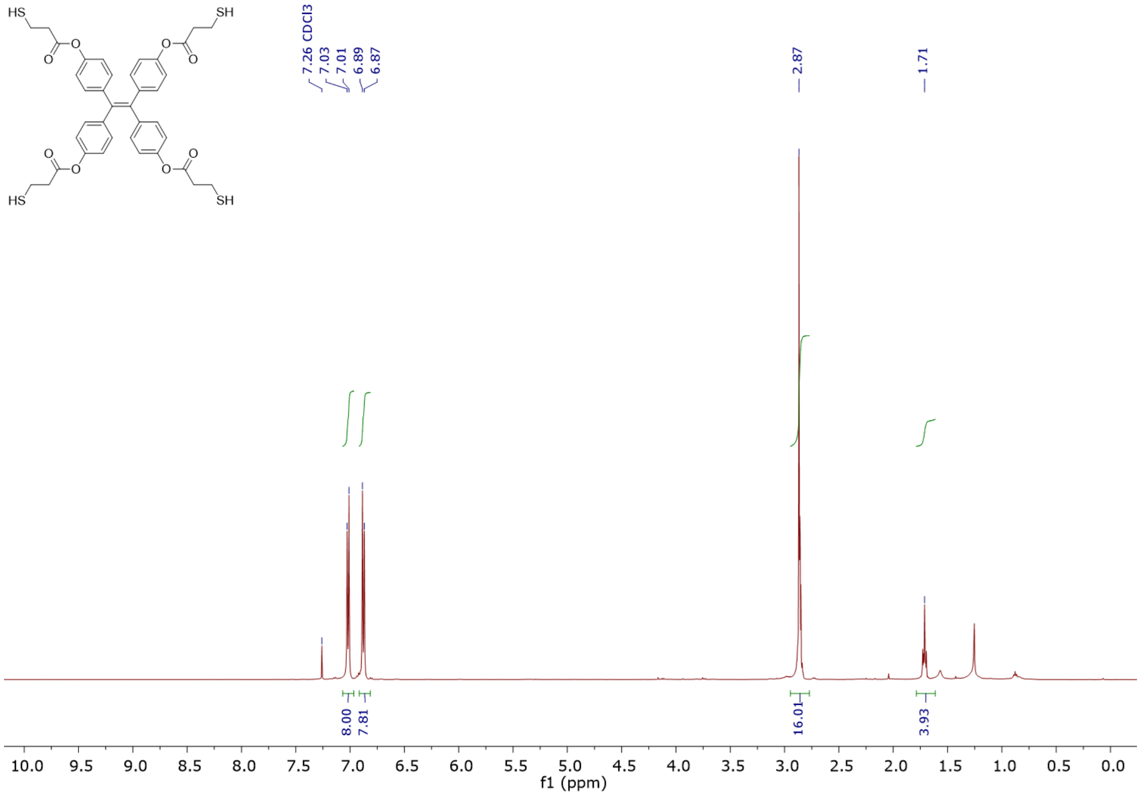
HS
SH
O
HS
SH
7.26 CDCl3
7.03
7.01
6.89
6.87
2.87
1.71
8.00
7.81
16.01
3.93
10.0 9.5 9.0 8.5 8.0 7.5 7.0 6.5 6.0 5.5 5.0 4.5 4.0 3.5 3.0 2.5 2.0 1.5 1.0 0.5 0.0
f1 (ppm)

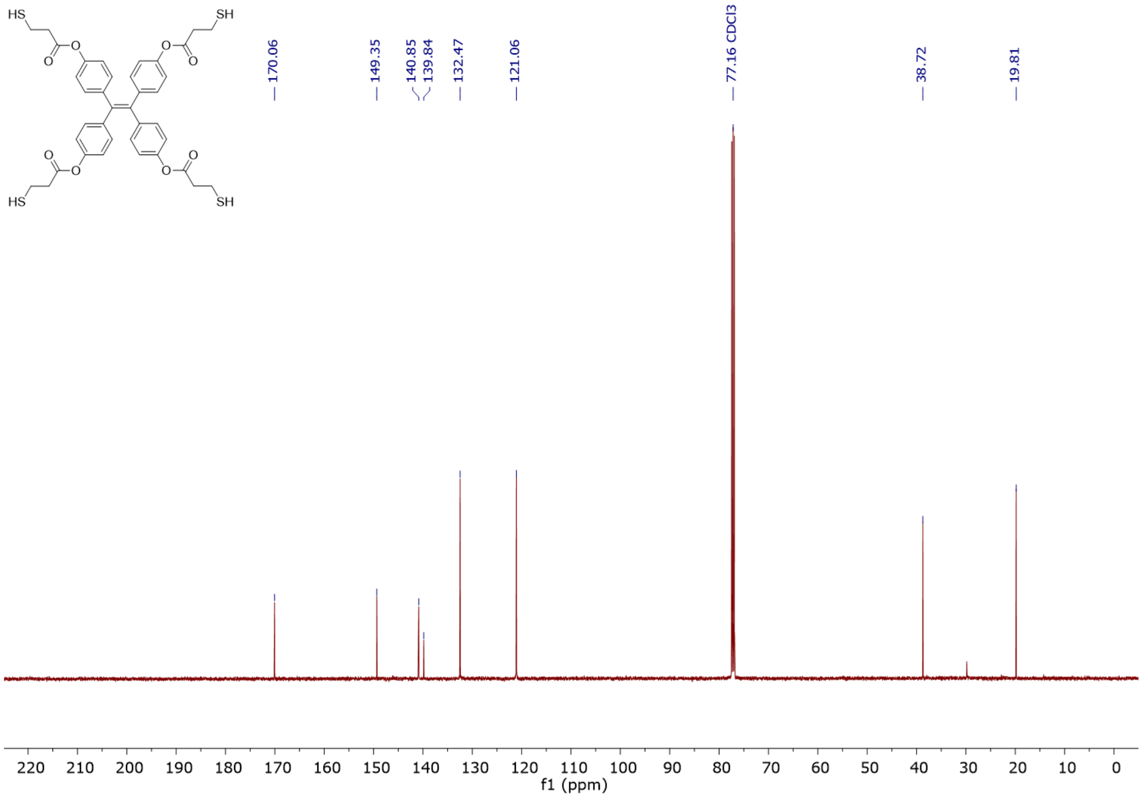
HS
SH
O
170.06
149.35
140.85
139.84
132.47
121.06
77.16 CDCl3
38.72
19.81
220 210 200 190 180 170 160 150 140 130 120 110 100 90 80 70 60 50 40 30 20 10 0
f1 (ppm)

Cage A

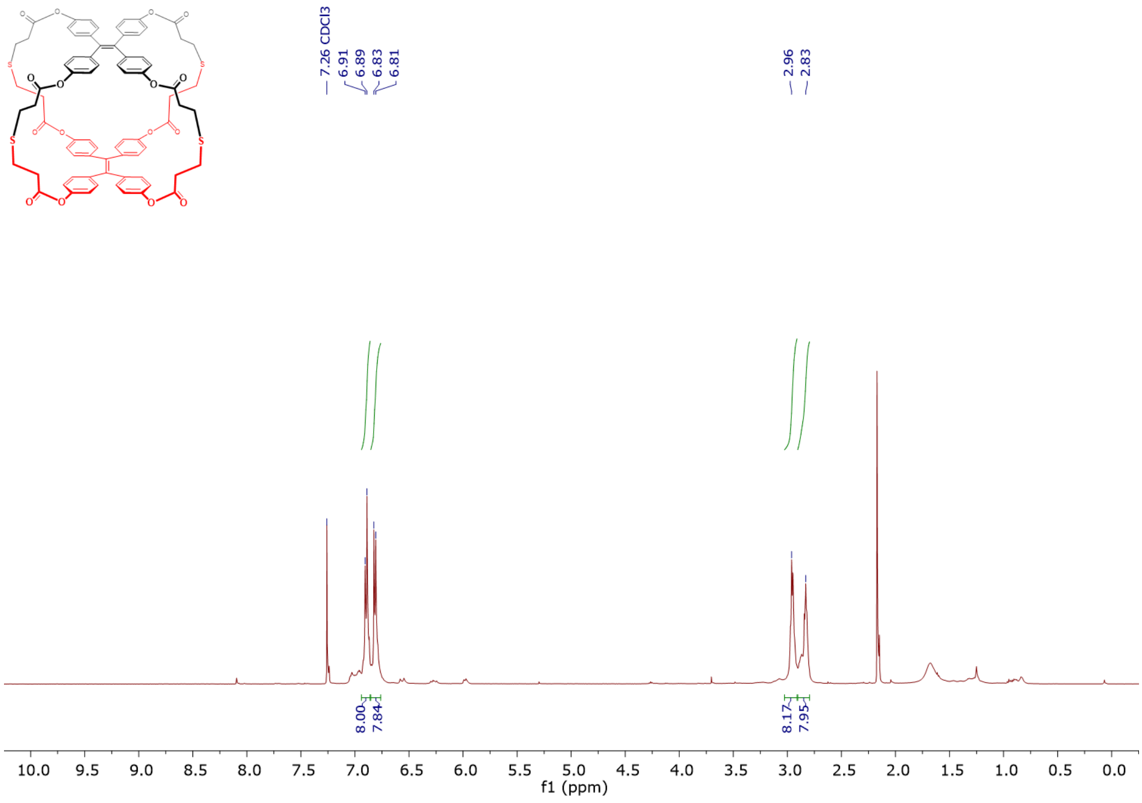
7.26 CDCl3
6.91
6.89
6.83
6.81
2.96
2.83
8.00
7.84
8.17
7.95
10.0 9.5 9.0 8.5 8.0 7.5 7.0 6.5 6.0 5.5 5.0 4.5 4.0 3.5 3.0 2.5 2.0 1.5 1.0 0.5 0.0
f1 (ppm)

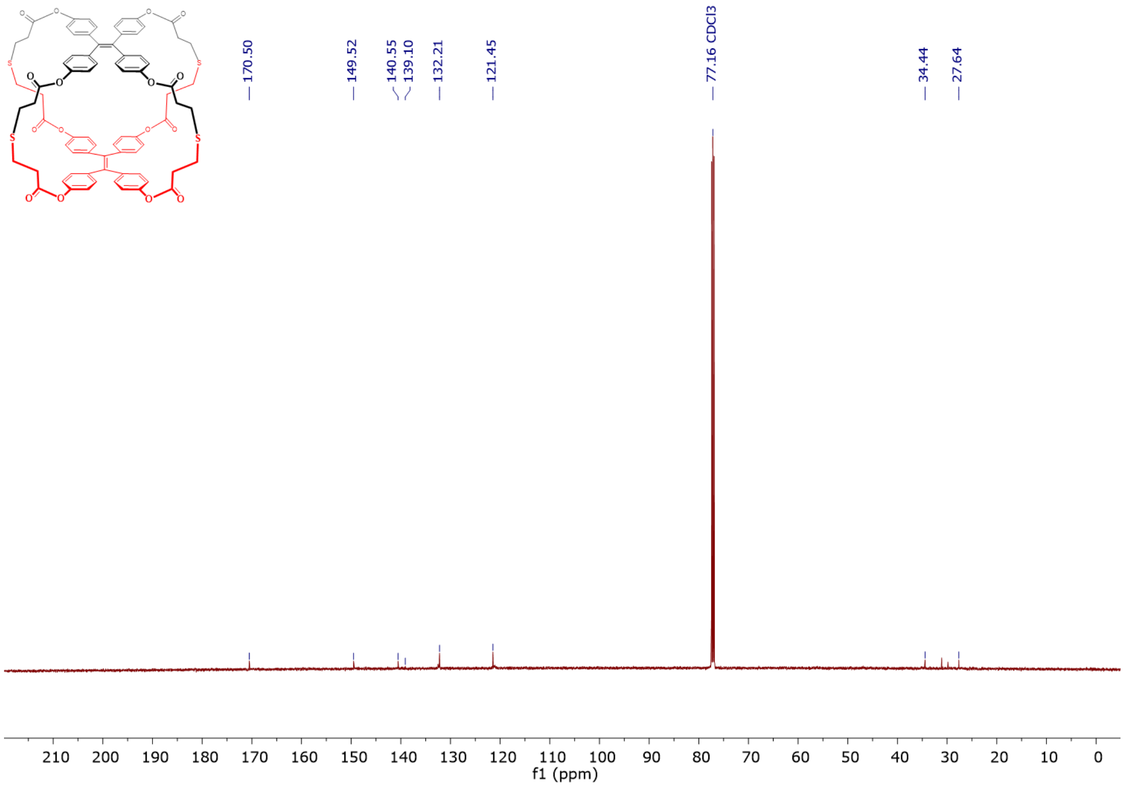

Elemental Composition Report **Page 1**

Multiple Mass Analysis: 5 mass(es) processed
Tolerance = 5.0 PPM / DBE: min = -1.5, max = 50.0
Element prediction: Off
Number of isotope peaks used for i-FIT = 3

Monoisotopic Mass, Even Electron Ions
2797 formula(e) evaluated with 9 results within limits (all results (up to 1000) for each mass)
Elements Used:
C: 0-80 H: 0-140 O: 0-20 Na: 0-1 S: 0-4
Lidia
(ESI-18) (160) Lidia (LP 36-4B Cap1) 31 (1.026)

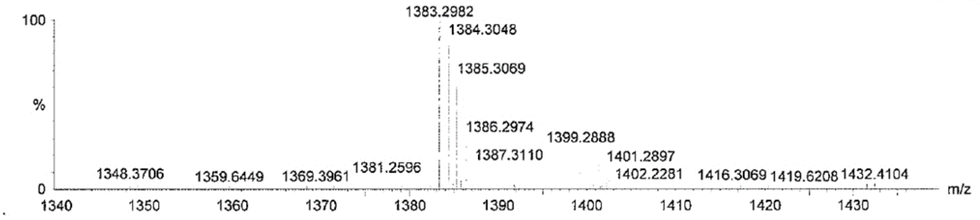

Minimum:	20.00				-1.5			
Maximum:	100.00		5.0	5.0	50.0			
Mass	RA	Calc. Mass	mDa	PPM	DBE	i-FIT	i-FIT (Norm)	Formula
1383.2982	100.00	1383.2999	-1.7	-1.2	47.5	32.2	0.6	C78 H63 O16 S4
		1383.2975	0.7	0.5	44.5	32.5	0.9	C76 H64 O16 Na S4

Cage B

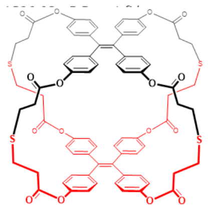

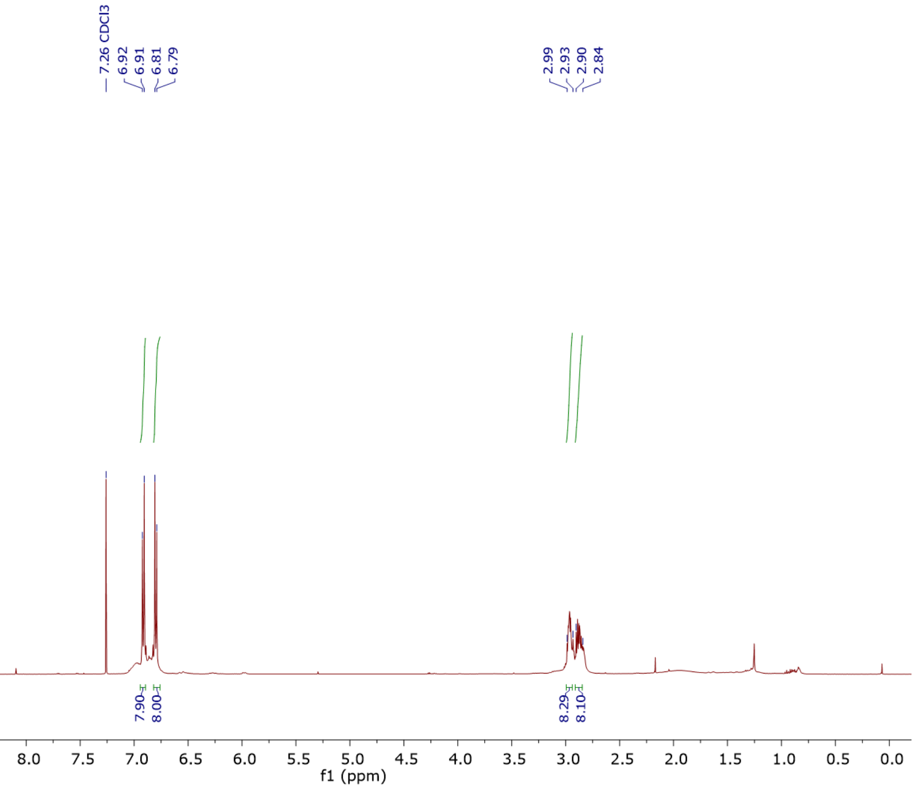

7.26 CDCl3
6.92
6.91
6.81
6.79
2.99
2.93
2.90
2.84
7.90
8.00
8.29
8.10
10.0 9.5 9.0 8.5 8.0 7.5 7.0 6.5 6.0 5.5 5.0 4.5 4.0 3.5 3.0 2.5 2.0 1.5 1.0 0.5 0.0
f1 (ppm)

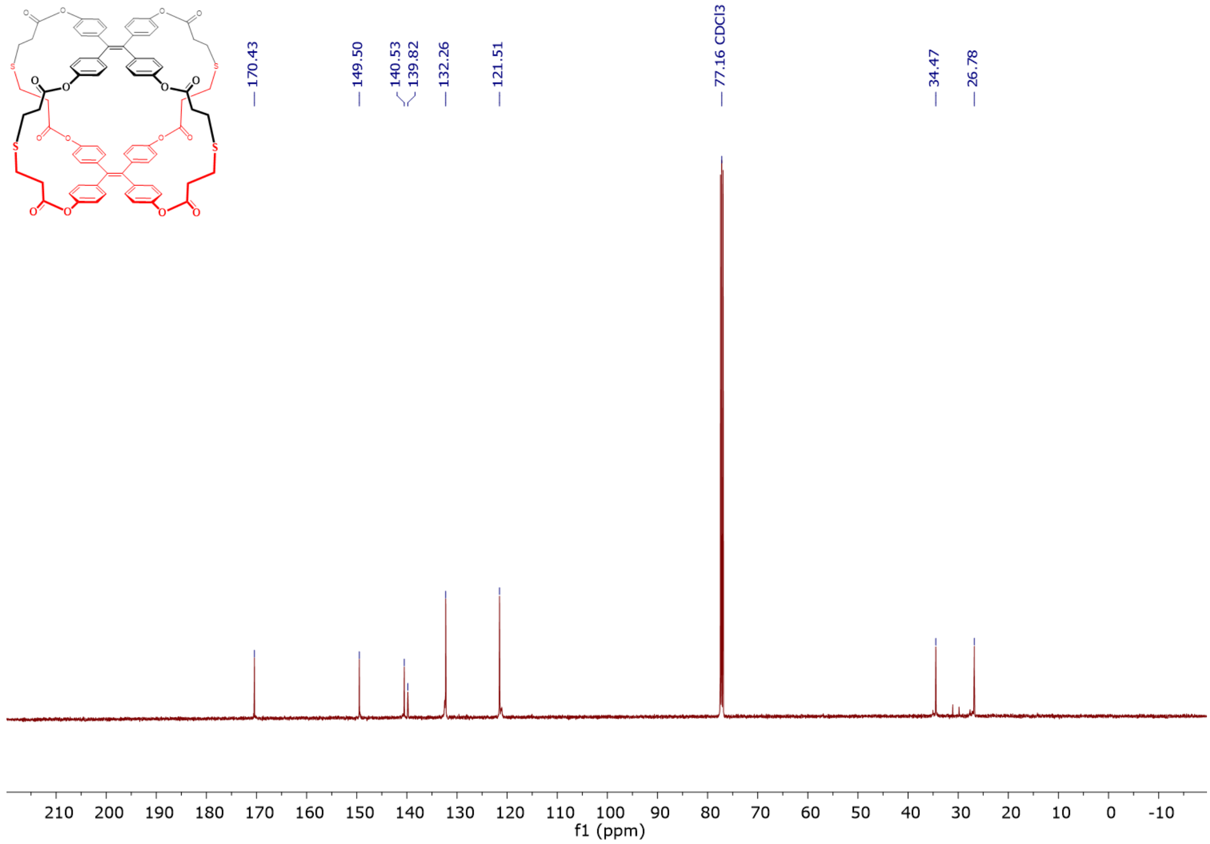

Elemental Composition Report **Page 1**

Multiple Mass Analysis: 4 mass(es) processed
Tolerance = 5.0 PPM / DBE: min = -1.5, max = 50.0
Element prediction: Off
Number of isotope peaks used for i-FIT = 3

Monoisotopic Mass, Even Electron Ions
2296 formula(e) evaluated with 8 results within limits (all results (up to 1000) for each mass)
Elements Used:
C: 0-80 H: 0-140 O: 0-20 Na: 0-1 S: 0-4
Lidia
(ESI-18) (161) Lidia (LP 36-4B Cap2) 11 (0.452)

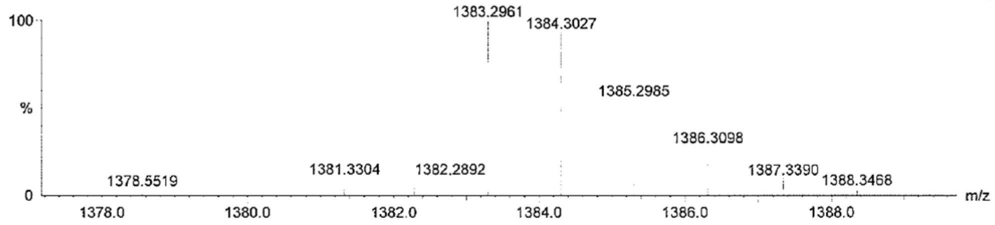

Mass	RA	Calc. Mass	mDa	PPM	DBE	i-FIT	i-FIT (Norm)	Formula
Minimum:	20.00				-1.5			
Maximum:	100.00		5.0	5.0	50.0			
1383.2961	100.00	1383.2941	2.0	1.4	49.5	10.1	0.8	C79 H60 O16 Na S3
		1383.2975	-1.4	-1.0	44.5	10.3	1.0	C76 H64 O16 Na S4

11. References

[1] Peng, A. Shi, Z. Dou, W. Cao, X. Zhang, H. *Org. Lett.* **2010**, *12*(19), 4364-4367

[2] Turan, I. Cakmak, F. Sozmen, F. *Tetrahedron Lett.* **2014**, *55*, 456-459.

[3] Zhang, M. Yin, X. Tian, T. Liang,Y. Li, W. Lan, Y. Li, J. Zhou, M. Ju, Y. Li, G. *Chem. Commun.* **2015**, *51*, 10210-10213.

[4] M. D. Perretti, L. A. Pérez-Márquez, R. García-Rodríguez, R. Carrillo, *J. Org. Chem.* **2019***, 84,* 840−850.

[5] J. R. Lakowicz, *Principles of Fluorescence Spectroscopy*, Springer, New York, 3rd edn, **2006**

[6] P. Thordarson, *Chem. Soc. Rev.* **2011**, *40*, 1305-1323.

[7] Corradini, R. Paganuzzi, C. Marchelli, R. Pagliari, S. Sforza, S. Dossena, A. Galavernaa, G. Duchateaub, A. *J. Mater. Chem.* **2005**,*15*, 2741-2746.

[8] B. Zhu, C. Gao, Y. Zhao, C. Liu, Y. Li, Q. Wei, Z. Ma, B. Du, X. Zhang, *Chem. Commun*. **2011**, *47*, 8656-8658.

[9] IUPAC. Compendium of Chemical Terminology, 2nd ed. (the "Gold Book"). Compiled by A. D. McNaught and A. Wilkinson. Blackwell Scientific Publications, Oxford (1997). Online version (2019) created by S. J. Chalk. ISBN 0-9678550-9-8. https://doi.org/10.1351/goldbook.

[10] D. Teixeira, C. Prudêncio, M. Vieira, *J. Chromatogr. B* **2017**, *1046*, 48-57.
